# RNA sequencing of peripheral blood in amyotrophic lateral sclerosis reveals distinct molecular subtypes: Considerations for biomarker discovery

**DOI:** 10.1111/nan.12943

**Published:** 2023-11-07

**Authors:** Natalie Grima, Sidong Liu, Dean Southwood, Lyndal Henden, Andrew Smith, Albert Lee, Dominic B. Rowe, Susan D'Silva, Ian P. Blair, Kelly L. Williams

**Affiliations:** ^1^ Motor Neuron Disease Research Centre Macquarie Medical School Faculty of Medicine, Health and Human Sciences Macquarie University Sydney NSW Australia; ^2^ Centre for Health Informatics Faculty of Medicine, Health and Human Sciences Macquarie University Sydney NSW Australia

**Keywords:** amyotrophic lateral sclerosis, biomarker, ferroptosis, immune response, metabolism, peripheral blood, RNA‐seq, transcriptome

## Abstract

**Aim:**

Amyotrophic lateral sclerosis (ALS) is a heterogeneous neurodegenerative disease with limited therapeutic options. A key factor limiting the development of effective therapeutics is the lack of disease biomarkers. We sought to assess whether biomarkers for diagnosis, prognosis or cohort stratification could be identified by RNA sequencing (RNA‐seq) of ALS patient peripheral blood.

**Methods:**

Whole blood RNA‐seq data were generated for 96 Australian sporadic ALS (sALS) cases and 48 healthy controls (NCBI GEO accession GSE234297). Differences in sALS–control gene expression, transcript usage and predicted leukocyte proportions were assessed, with pathway analysis used to predict the activity state of biological processes. Weighted Gene Co‐expression Network Analysis (WGCNA) and machine learning algorithms were applied to search for diagnostic and prognostic gene expression patterns. Unsupervised clustering analysis was employed to determine whether sALS patient subgroups could be detected.

**Results:**

Two hundred and forty‐five differentially expressed genes were identified in sALS patients relative to controls, with enrichment of immune, metabolic and stress‐related pathways. sALS patients also demonstrated switches in transcript usage across a small set of genes. We established a classification model that distinguished sALS patients from controls with an accuracy of 78% (sensitivity: 79%, specificity: 75%) using the expression of 20 genes. Clustering analysis identified four patient subgroups with gene expression signatures and immune cell proportions reflective of distinct peripheral effects.

**Conclusions:**

Our findings suggest that peripheral blood RNA‐seq can identify diagnostic biomarkers and distinguish molecular subtypes of sALS patients however, its prognostic value requires further investigation.

## Graphical abstract

Peripheral blood RNA‐seq from 96 sporadic ALS cases and 48 controls were examined for diagnostic, prognostic and cohort stratification biomarkers. Biomarkers distinguishing patients with ALS from controls were identified including activation of ferroptosis and immune‐related pathways, and differential transcript usage. Machine learning algorithms classified patients with ALS from controls with 78% accuracy yet were unable to accurately predict survival. Four subgroups of patients with ALS with distinct gene expression profiles and immune cell proportions were identified by unsupervised clustering analysis, highlighting heterogeneity of the patients.

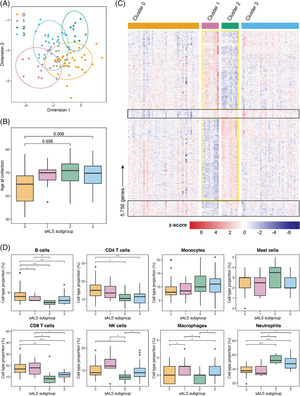

**Key points**

- Peripheral blood RNA‐seq data from 96 sporadic ALS (sALS) cases and 48 controls were examined for diagnostic, prognostic and subgroup stratification biomarkers.
- Peripheral blood RNA‐seq identified biomarkers distinguishing sALS patients from controls including activation of ferroptosis and immune‐related pathways, and differential transcript usage.
- Machine learning algorithms classified sALS patients from controls with 78% accuracy yet were unable to accurately predict survival.
- Four sALS patient subgroups with distinct gene expression profiles and immune cell proportions were identified by unsupervised clustering analysis.
- We suggest that the observed heterogeneity in sALS blood gene expression profiles complicates diagnostic and prognostic biomarker discovery and recommend that future RNA‐seq studies employ longitudinal samples and/or brain–blood matched cohorts.

## INTRODUCTION

Amyotrophic lateral sclerosis (ALS) is an adult‐onset, neurodegenerative disorder caused by the progressive loss of motor neurons in the brain and spinal cord. While peripheral blood is not a primary site of disease in ALS, its accessibility has made it a prime target in the search for disease biomarkers. Several blood‐based biomarkers have been reported for ALS including neurofilaments, immune cells (e.g., altered T‐regulatory cells), cytokines (e.g., IFN‐γ, interleukins), microRNAs (e.g., miR206) and metabolites (e.g., serum creatinine, glutamate, uric acid) (reviewed in [1]). Several of these biomarkers have also been reported in cerebrospinal fluid (CSF). While more proximal to the central nervous system (CNS), obtaining CSF is highly invasive and difficult to serially sample throughout disease progression. Individual candidate biomarkers for ALS have demonstrated variable clinical utility when used alone and there is a lack of consistent cross‐validation. The absence of robust biomarkers for patient diagnosis, prognosis, cohort stratification and monitoring therapeutic response is a key limiting factor in the development of effective therapies in ALS.

A proposed method of improving ALS biomarker performance is to combine multiple blood‐based biomarkers or to pair them with biomarkers from other modalities such as genetic risk factors, neuroimaging or electrophysiology [1]. An alternate and increasingly feasible approach is to examine more global molecular signatures by applying ‐omic technologies to ALS patient biospecimens. Transcriptomics is particularly apt for capturing a global snapshot of biological pathways under disease conditions and several studies have utilised gene expression microarrays or RNA sequencing (RNA‐seq) to profile gene expression in ALS patient whole blood [2, 3, 4] or peripheral blood mononuclear cells [5, 6, 7, 8, 9, 10]. An important consideration in ALS population studies is the high clinical and pathological heterogeneity that may complicate the detection of differences relative to control individuals. This is critical when examining high‐dimensional data, such as RNA‐seq, where large sample sizes are required to detect patient subgroups amongst highly variable data. The largest ALS‐control blood gene expression data set reported to date consists of 397 ALS patients, 645 controls and 75 ALS‐mimics assessed by microarray [3]. Subsequent re‐interrogation of this dataset reported a high‐accuracy diagnostic model, patient subgroups, and a gene panel predictive of patient survival; however, these findings remain to be validated in independent cohorts [11].

Herein, we describe a peripheral blood RNA‐seq data set consisting of 96 sporadic ALS (sALS) cases and 48 matched control participants. We performed a comprehensive analysis to identify candidate biomarkers for ALS patient diagnosis, prognosis and stratification. This identified gene‐ and, for the first time, transcript‐level expression differences between sALS patients and controls. We used machine learning to develop an accurate diagnostic model (sALS vs control) but report that whole blood gene expression was less informative for the prediction of patient phenotype including survival. Finally, we employed unsupervised clustering analysis to distinguish ALS patient subgroups with distinct gene expression signatures and predicted immune cell proportions. This RNA‐seq dataset is publicly available and can be accessed at NCBI Gene Expression Omnibus (GEO) under GSE234297.

## MATERIALS AND METHODS

### Study cohort

96 sALS patients and 48 neurologically normal control participants were acquired from the Macquarie University Neurodegenerative Disease Biobank (Table 1). sALS cases were clinically diagnosed according to El Escorial criteria [12] and were classified as sporadic based on the absence of a family history of neurodegenerative disease. All sALS patients were screened for pathogenic repeat expansions in *C9orf72* by repeat‐primed PCR and fragment analysis (method described in [13]), with >30 repeats defined as pathogenic. sALS patients with whole‐genome sequencing data available (88/96) were screened for known pathogenic ALS gene variants. De‐identified demographic, clinical and sample characteristics are presented in Table S1.

**TABLE 1:** Demographic and clinical information for the study cohort.

	sALS	Control
Donors	96	48
Sex (males)	58 (60.4%)	27 (56.3%)
Age at collection (years)	63.4 ± 13.8	60.6 ± 12.2
Age at disease onset (years)	60.3 ± 14.0	‐
Disease duration (months)	69.4 ± 51.4	‐
Site of onset		
Bulbar	21 (22.1%)	‐
Spinal	74 (77.9%)	‐
Participants with pathogenic ALS variant	1 (1.0%)	‐

Age at collection, age at disease onset and disease duration are presented as mean ± standard deviation. The site of onset was unavailable for one sALS patient. The participant with the reported ALS variant has a *C9orf72* repeat expansion.

Abbreviation: sALS, sporadic amyotrophic lateral sclerosis.

### RNA extraction and sequencing

Peripheral blood (i.e., whole blood including plasma) was collected in PAXgene Blood RNA tubes (BD Biosciences, NJ, USA). Total RNA was extracted from peripheral blood using the QIASymphony automated liquid handing robot and the PAXgene Blood RNA kit (Qiagen, Hilden, Germany) as per standard protocol. RNA integrity was measured using the Agilent RNA 6000 Nano assay on the Agilent 2100 Bioanalyzer system (Agilent Technologies, CA, USA). Samples with RNA integrity number (RIN) ≥ 5 were subjected to RNA‐seq. RNA‐seq libraries were prepared from total RNA using the TruSeq Stranded mRNA Sample Prep kit (Illumina, CA, USA). Sequencing was performed on an Illumina HiSeq 2000 platform (2 × 125bp paired‐end reads with target sequencing depth of 32 million reads/sample) generating raw sequencing reads in FASTQ format (Centre for Brain Genomics, Queensland Brain Institute, University of Queensland).

### RNA‐seq data processing

Pre‐processing quality control of raw FASTQ files was performed using Trimmomatic v0.38 to remove low‐quality reads and adaptor sequences [14] followed by a quality check using FastQC v0.11.7 (http://www.bioinformatics.babraham.ac.uk/projects/fastqc/). As GC bias was observed in the data set, Salmon v1.9.0 was used for transcript quantification due to its inbuilt means of correcting GC biases [15]. The transcriptome index was built using the NCBI *Homo sapiens* transcriptome (annotation release 109.20200522), with the corresponding genome assembly (GRCh38.p13) used as the decoy sequence. Transcripts were quantified in mapping‐based mode using the GRCh38 transcriptome and default selective alignment with –seqBias and –gcBias flags. Resultant transcript abundance estimates were converted to gene‐level counts using tximport v1.26.1 [16] and an offset matrix, incorporating Salmon‐determined biases and sequencing depth, was generated.

### Confounder identification and adjustment

All following analyses were performed in R [17] unless specified otherwise. Following filtering of low‐count genes using edgeR v3.38.4 filterByExpr function, raw gene counts were normalised and log‐transformed using the tximport‐generated offset matrix and edgeR cpm function [18]. Principal component analysis (PCA) was performed on the resulting expression matrix using prcomp. To identify potential confounders, biological and technical variables were visualised by 2D plotting of principal components (PCs). For covariates, Spearman's rank correlation coefficient was calculated for each covariate against the top 10 PCs. In addition, the limma selectModel function was used to determine the best‐fitting linear model for each expressed gene based on the minimisation of the Bayesian Information Criterion (BIC) [19]. The number of genes with a lower BIC in the tested model was used to indicate whether a given variable improved the base model containing only case–control status as a predictor. Sex and sample GC content were identified as confounders (Figure S1) and were included in all linear models (∼Sex + GC content + disease status).

### Differential gene expression analysis

Differential gene expression analysis between sALS patients and controls was performed using edgeR v3.38.4 [18]. Specifically, the edgeR robust method was used to estimate negative binomial dispersion [20] followed by quasi‐likelihood dispersion estimation and F‐test for differential expression. Genes with a Benjamini–Hochberg adjusted *p*‐value (FDR) < 0.05 were considered differentially expressed. A second differential gene expression analysis was performed in the same manner between sALS cases whose blood collection occurred early in disease duration vs late in disease duration (Figure S2). After removing non‐deceased individuals without a disease duration measurement (n = 12), the early‐ and late‐stage collection groups were defined as the 33rd and 66th percentiles, respectively (n = 28 each). Early‐stage collection captured individuals at 0.15–0.45 in disease duration while late‐stage collection captured individuals at 0.73–0.98 in disease duration (0 represents time of disease onset and 1 represents death). Early‐ and late‐stage collection groups were each compared with controls (n = 48) and directly to each other.

Differentially expressed genes were submitted to Metascape 3.5 (http://metascape.org) [21] for Gene Ontology (GO) biological processes and Kyoto Encyclopedia of Genes and Genomes (KEGG) pathway enrichment analysis. Default parameters (minimum overlap = 3, *p*‐value cut‐off = 0.01, minimum enrichment = 1.5) were applied. Differential gene expression results were also analysed with the use of QIAGEN IPA (QIAGEN Inc., https://digitalinsights.qiagen.com/IPA) [22]. Pathway analysis results were filtered for pathways with a *p*‐value of overlap <0.05 and absolute z‐score >0. Upstream analysis results were filtered for endogenous molecules with a predicted activation state.

### Differential transcript usage analysis

To identify differentially used transcripts, IsoformSwitchAnalyzeR v1.18.0 [23, 24] was applied to the Salmon transcript quantification using the DEXSeq implementation of the isoform switch test. Default parameters were used with significant isoform switches defined as FDR < 0.05 and ≥10% change in isoform usage. Genes identified to involve isoform switches were annotated with CPC2 [25], Pfam [26], SignalP [27] and NetSurfP‐2 [28].

To further consider de novo transcripts, trimmed reads were aligned to the human genome assembly using the splice‐aware aligner HISAT2 v2.1.0 [29]. Resultant BAM files were input to StringTie v2.1.3b [30] excluding the ‐e option, which assembled transcripts using annotation release 109.20200522 as reference. StringTie was then run in merge mode to generate a transcriptome annotation containing both known and de novo transcripts. Transcripts were re‐assembled using the de novo transcriptome as reference and StringTie ‐eb options, generating read coverage tables for input into IsoformSwitchAnalyzeR.

### Cell‐type deconvolution analysis

Gene‐level transcripts per million (TPM) were retrieved from Salmon transcript abundance estimates using tximport [16]. The LM22 signature matrix and source gene expression profile were downloaded from CIBERSORTx [31]. Gene symbols were updated using the HUGO Gene Nomenclature Committee multi‐symbol checker (https://www.genenames.org/tools/multi-symbol-checker/) with a final overlap of 99.4% (11,773/11,845) genes between the blood RNA‐seq and LM22 data sets. Proportions of 22 leukocytes were estimated using CIBERSORTx B‐mode batch correction and were recategorised into 12 major leukocyte types by summing the proportions of their subsets. The 12 leukocyte types used were (1) B cells (naive and memory), (2) plasma cells, (3) CD8 + T cells, (4) CD4 + T cells (naive, memory resting, memory activated, T cells follicular helper and T cells regulatory), (5) γδ T cells, (6) NK cells (resting and activated), (7) monocytes, (8) macrophages (M0, M1, M2), (9) dendritic cells (resting and activated), (10) mast cells (resting and activated), (11) eosinophils and (12) neutrophils.

### Count normalisation for unsupervised analyses

Following filtering of low count genes using edgeR filterByExpr function (12,569 genes remaining), gene counts from all 144 samples underwent either (1) variance stabilising transformation (VST) using DESeq2 vst function [32] or (2) transformation to log counts per million (CPM) using edgeR cpm function [18]. The limma removeBatchEffect function [19] was then applied to transformed counts to correct for mean GC content. Transformation 1 (VST) was used for weighted gene co‐expression network analysis (WGCNA) and clustering analysis because of improved stabilisation of the variance across expression levels (Figure S3). Both transformations (VST and CPM) were tested in machine learning algorithms. For clustering analysis, sex‐linked genes (Y chromosome and *XIST*) were also removed.

### Weighted gene co‐expression network analysis

Weighted gene co‐expression network analysis was performed using the WGCNA package in R v1.72.1 [33] using the 96 sALS cases only. The association of module eigengenes with clinical traits were calculated using Spearman correlation. For modules identified to be significantly associated with clinical traits (FDR < 0.05), GO biological processes and KEGG pathway enrichment analyses were performed using Metascape 3.5. The modulePreservation function was used to determine whether co‐expression modules identified in sALS were preserved in control expression data.

### sALS clustering

sALS normalised counts underwent dimension reduction by PCA. The number of PCs (dimensions) was set to 3–50 and the sum of explained variance ratio was calculated. The lowest number of PCs that satisfied 60% of the sum of explained variance ratio was chosen (n = 19). The Elbow Method was used to determine the optimal number of clusters in the data set for K‐means clustering. This returned an optimal number of 5 clusters; however, as one cluster contained a single sample, k = 4 was used for subsequent analysis. To identify genes defining each subgroup relative to all other subgroups, differential gene expression analysis was performed as previously described using the model ∼0 + cluster membership + Sex + GC content. To account for subgroup‐specific variability and to prevent bias towards larger subgroups [34], direct comparisons were made between each subgroup (3 comparisons per subgroup). The overlap of genes identified as significantly up‐ or down‐regulated from each comparison (FDR < 0.05) was classified as subgroup‐defining genes. For direct comparisons between clusters 0 and 3, significant differentially expressed genes were further filtered for genes with a fold‐change >1.5 or <0.67.

### Machine learning

A machine learning pipeline consisting of three major components was implemented: (1) a feature selection model to identify genes that are predictive of disease status and duration, (2) a classification model to differentiate sALS cases and controls and (3) a regression model to predict disease duration.

#### Feature selection

The Hilbert Schmidt Independence Criterion Lasso (HSIC Lasso) [35] feature selection algorithm was implemented based on the pyHISCLasso library v1.4.2 (https://pypi.org/project/pyHSICLasso/). By default, the Gaussian kernels were used to model feature distributions and the top 20 features were selected based on their feature relatedness score.

##### Classification model

To identify genes that can differentiate sALS cases from controls, we followed a previous study [36] and used the selected features to train a Random Forest model [37] for sALS and control classification. Default parameters (number of trees: 100; max depth of the tree: 5) and a classification probability ≥60% were used. The Random Forest classification model was implemented based on the scikit‐learn library v0.23.2 (https://scikit-learn.org/).

##### Regression model

Disease duration distribution was heavily skewed, with the majority (60%) of cases having disease durations <5 years (Figure 3A). To model the tailed distribution of the disease duration values, we used a Tweedie distribution [38] with a power parameter of r = 1.6. A generalised linear model based on the Tweedie distribution was then built to predict sALS patients' disease duration. The Tweedie regression models were implemented in Python based on the scikit‐learn library.

##### Performance evaluation

Leave‐One‐Out (LOO) cross‐validation was used to evaluate the effectiveness of selected features in classification and regression models. Validation of the classification model was also tested on an independent whole blood RNA‐seq data set consisting of 30 ALS cases and 30 controls [39] (Figure S4). All machine learning was conducted in Python v3.8.2 [40].

Additional methodological details can be found in Supplementary Methods.

## RESULTS

### sALS peripheral blood RNA‐seq cohort

A summary of the demographic and clinical information for the 96 sALS patients and 48 neurologically normal control participants is provided in Table 1. sALS patient and control groups were matched for sex, age at collection and technical sample features including RIN (Figure S5, Table S2). Sampling of sALS peripheral blood occurred at varying points in disease duration, ranging from 0.12 to 0.98, where 0 represents time of disease onset and 1 represents death, herein referred to as “collection point” (Figure S2). Samples had a mean read count of 29.6 million reads post trimming (range: 11.9–87.7 million) of which 93.6% were high‐quality (i.e., Q30%; range: 85.9%–97.2%). Power analysis for a two‐sample t‐test determined that this cohort has the power to detect medium to large (Cohen's d ≥ 0.5) effect sizes (Figure S6).

### Peripheral blood diagnostic biomarkers for sALS

#### Genes differentially expressed in sALS highlighted broad alterations in immune, metabolic and stress‐related pathways

**FIGURE 1:**
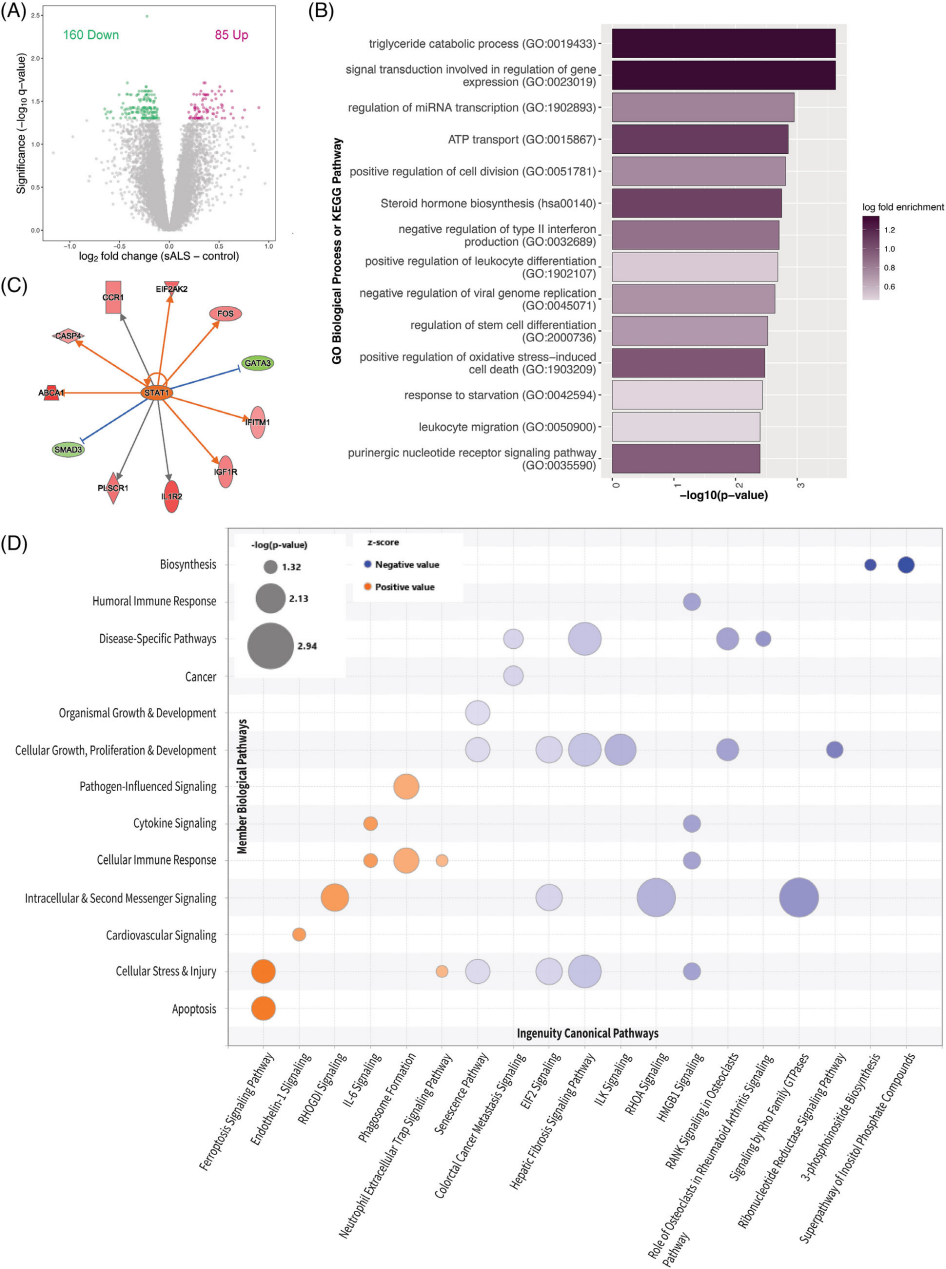
Differentially expressed genes indicated alteration of metabolic pathways, immune response and cell stress in sALS peripheral blood. (A) Volcano plot comparing sALS patients with controls. Pink and green dots represent genes that were upregulated and downregulated, respectively (FDR < 0.05) while grey dots are genes that were not differentially expressed. (B) GO biological processes and KEGG pathways enriched in the sALS–control differentially expressed genes. The most representative member (lowest *p*‐value) of each GO term cluster is displayed. Log2 fold change indicates enrichment of member genes in differentially expressed genes vs all detected genes. **(C)** Example IPA upstream analysis network for transcription factor STAT1, which was predicted to be activated in sALS. Red genes were upregulated and green genes were downregulated in sALS vs controls. Orange arrows indicate predicted activation, blue lines indicate predicted inhibition and grey arrows indicate no effect predicted. (D) Biological pathways predicted by IPA to have altered activity in sALS based on differentially expressed genes. Individual bubbles represent ingenuity canonical pathways (x‐axis), which have been categorised into their member biological pathways (y‐axis). Orange and blue bubbles represent pathways predicted to be activated or inhibited, respectively (z‐score). The bubble size represents the *p*‐value, which indicates the probability of association of differentially expressed genes with the ingenuity canonical pathway by random chance alone (right‐tailed Fisher's exact test).

Two hundred and forty‐five genes (85 upregulated, 160 downregulated) were identified to be differentially expressed between sALS cases and controls at FDR < 0.05 (Figure 1A). Of the significant genes, 221 were protein‐coding, 20 were lncRNA, three were pseudogenes and one was a V gene segment (Table S3). Large fold changes in gene expression were not observed between sALS cases and controls, with no significant differentially expressed genes demonstrating a fold change >2. The GO biological processes and KEGG pathways that were enriched among sALS differentially expressed genes broadly represented metabolic pathways, regulation of gene expression, immune response and cell stress (Figure 1B; Table S4).

Ingenuity Pathway Analysis (IPA) considers the direction (upregulation or downregulation) of differentially expressed genes and was used to predict the activation state of molecules and biological pathways. Twenty‐five molecules were predicted to have altered activity in sALS (17 activated, 8 inhibited) based on gene expression of downstream targets (Table 2). Top molecules predicted to be activated were predominantly involved in immune and inflammatory response including cytokines (interferons, tumour necrosis factor, colony stimulating factor), transcription factor STAT1 (Figure 1C) and transporter SLC15A4. Among the top inhibited molecules were transcription factor myc, immunoglobulin and Akt (protein kinase B) (Table 2). At the pathway level, 19 ingenuity canonical pathways were predicted to have altered activity in sALS, which were more broadly categorised into 13 biological pathways (Figure 1D). The ferroptosis signalling pathway, categorised under cellular stress and injury and apoptosis, was the highest‐ranking canonical pathway predicted to be activated in sALS. Again, cellular immune response demonstrated activation, including IL‐6 signalling and phagosome formation. Inhibited pathways included biosynthesis of inositol phosphates, cellular growth, proliferation and development and HMGB1 signalling. An additional 43 ingenuity canonical pathways were enriched among sALS differentially expressed genes with no predicted activation state (Table S5). Enriched pathways were largely concordant with Metascape findings including pathways involved in immune response (e.g., B cell activating factor signalling, IL‐7 signalling, CXCR4 signalling), metabolism (e.g., triacylglycerol degradation, d‐mannose degradation) and cell stress (e.g., DNA Double‐Strand Break Repair by Non‐Homologous End Joining).

**TABLE 2:** Molecules predicted by ingenuity pathway analysis (IPA) to have altered activity in sALS based on differential expression of downstream genes. Z‐score indicates predicted activation (positive value) or inhibition (negative value).

Upstream regulator	Molecule type	Predicted state	z‐score
Interferon alpha	Group	Activated	3.07
STAT1	Transcription regulator	Activated	2.77
OSM	Cytokine	Activated	2.69
Tnf (family)	Group	Activated	2.63
IFNG	Cytokine	Activated	2.4
TNFSF10	Cytokine	Activated	2.39
ID3	Transcription regulator	Activated	2.38
prostaglandin E2	Chemical ‐ endogenous	Activated	2.37
ESR1	Ligand‐dependent nuclear receptor	Activated	2.37
CSF	Group	Activated	2.24
CSF3	Cytokine	Activated	2.2
FSH	Complex	Activated	2.2
HIF1A	Transcription regulator	Activated	2.19
SLC15A4	Transporter	Activated	2.18
IFN Beta	Group	Activated	2.16
EHMT1	Transcription regulator	Activated	2
COPS5	Transcription regulator	Activated	2
STAT5a/b	Group	Inhibited	−2
RNASEH2B	Other	Inhibited	‐2
CASR	G‐protein coupled receptor	Inhibited	‐2
SCD	Enzyme	Inhibited	‐2
TLR2	Transmembrane receptor	Inhibited	‐2
Akt	Group	Inhibited	−2.19
Immunoglobulin	Complex	Inhibited	−2.41
MYC	Transcription regulator	Inhibited	−3.21

Abbreviation: sALS, sporadic amyotrophic lateral sclerosis.

#### sALS differential transcript usage was detected in a small set of genes

**FIGURE 2:**
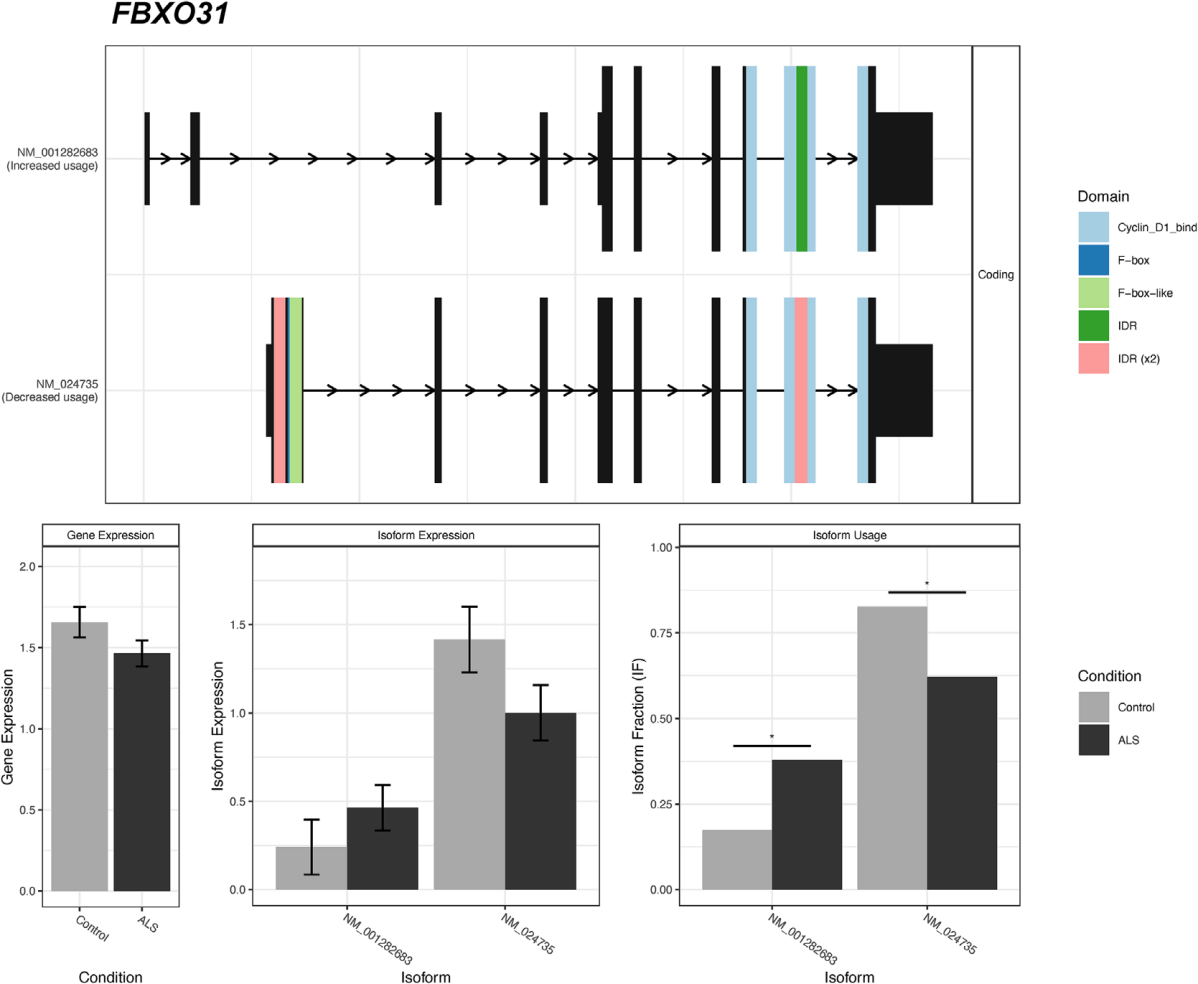
Fifteen genes were identified to have differential transcript usage between sALS patients and controls. *FBXO31* is shown as a representative example (see Figure S5 for other genes). *Top panel*: visual depiction of assessed transcripts and their functional domains. *Bottom panel*: comparison of gene expression (left), isoform expression (middle) and isoform fraction (right) between control and ALS patient groups. Significant differences are indicated by an asterisk.

Six genes were identified to have differential isoform usage between sALS patients and controls (Figure 2, Table 3). *COG2* was the only gene also identified as differentially expressed and was predicted to be downregulated in sALS (−0.26 log fold change). When the same analysis was performed on the de novo transcriptome quantification, nine independent genes were identified to have differential isoform usage between sALS patients and controls (Table 3). None of these nine genes were identified as differentially expressed at the gene level. Of the 15 genes identified to demonstrate differential transcript usage, 11 were predicted to have a functional consequence (Figure S7).

**TABLE 3:** Genes with significant differential transcript usage between sALS patients and controls as identified by IsoformSwitchAnalyzeR. Transcripts were quantified against either the known (Salmon) or de novo (StringTie) transcriptome.

Transcriptome	Gene symbol	Gene name	dIF	q value	Consequence
Known	*FBXO31*	F‐box protein 31	0.41	4.35E‐02	TRUE
Known	*COG2*	Component of oligomeric golgi complex 2	0.24	3.18E‐06	FALSE
Known	*LOC112267855*	Uncharacterised LOC112267855	0.21	6.64E‐05	FALSE
Known	*DYNC1I2*	Dynein cytoplasmic 1 intermediate chain 2	0.1	4.35E‐02	FALSE
Known	*C2orf76*	Chromosome 2 open reading frame 76	0.1	9.84E‐06	TRUE
Known	*ACYP1*	Acylphosphatase 1	0.1	2.81E‐03	TRUE
De novo	*HADH*	Hydroxyacyl‐CoA dehydrogenase	0.44	1.80E‐02	TRUE
De novo	*INTS9*	Integrator complex subunit 9	0.34	3.00E‐02	TRUE
De novo	*KCTD7*	Potassium channel tetramerisation domain containing 7	0.34	1.63E‐03	TRUE
De novo	*NAE1*	NEDD8 activating enzyme E1 subunit 1	0.25	1.44E‐02	TRUE
De novo	*SMAP1*	Small ArfGAP 1	0.14	9.07E‐03	TRUE
De novo	*DVL1*	Dishevelled segment polarity protein 1	0.14	3.93E‐02	TRUE
De novo	*MR1*	Major histocompatibility complex, class I‐related	0.14	2.52E‐02	TRUE
De novo	*TOMM40*	Translocase of outer mitochondrial membrane 40	0.11	7.58E‐03	TRUE
De novo	*PDXDC1*	Pyridoxal dependent de‐carboxylase domain containing 1	0.1	7.58E‐05	FALSE

q value, FDR corrected p‐value; Consequence, whether IsoformSwitchAnalyzeR predicted functional consequences (e.g., gain or loss of protein domains).

Abbreviation: dIF, difference in isoform fraction; sALS, sporadic amyotrophic lateral sclerosis.

#### Blood gene expression profiles classified sALS patients from controls with 78% accuracy

**FIGURE 3:**
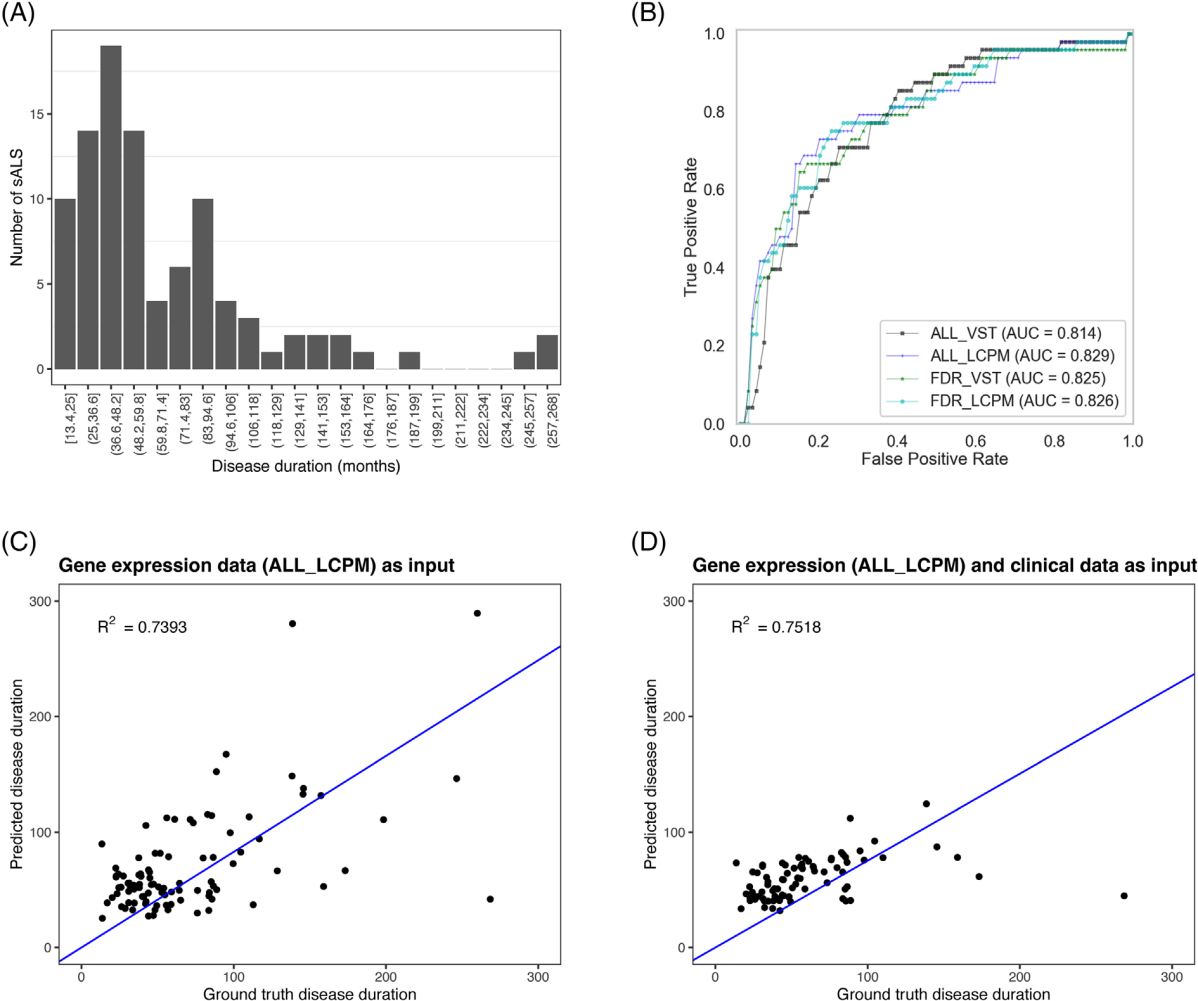
Application of machine learning to peripheral blood gene expression accurately distinguished sALS patients from controls. (A) The distribution of sALS patient disease duration was heavily skewed. (B) The ROC curves of the sALS–control classification results using the top 20 genes identified from different input feature sets. (C), (D) Scatter plots of the true disease duration vs predicted disease duration using regression models. Gene expression data without (C) or with (D) clinical data (sex, age at collection, site of onset) were used as input.

A classification model was built using four different feature sets as input: VST counts for (1) all 12,569 detected genes, (2) 245 sALS–control differentially expressed genes, or log CPM (LCPM) for (3) all 12,569 detected genes and (4) 245 sALS–control differentially expressed genes. The top 20 genes identified from feature set 3 using the LOO strategy achieved the best performance with an accuracy of 77.8% (sensitivity: 79.2%, specificity: 75%, AUC: 0.829) (Figure 3B). These genes were *RPL7L1, LOC107984421, PLEKHG4, MED13L, LETMD1, ZNF544, ELAC2, STAG3L4, FAM30A, NFYC‐AS1, RGS6, SREBF2, BICDL1, RPS10‐ NUDT3, COG2, PLEKHG5, GPN2, ERG28, PIK3IP1* and *IL1R2* (Table S6). Notably, 18/20 of these genes were identified as differentially expressed between sALS patients and controls. The best‐performing classification model was applied to normalised gene expression counts derived from an independent ALS‐control whole blood RNA‐seq data set (n = 60) [39]. This resulted in an ALS‐control classification accuracy of 63.3% (sensitivity: 60.0%, specificity: 66.7%, AUC: 64.7%).

#### Cell type deconvolution predicted increased circulating neutrophils in sALS

Cell type deconvolution of whole blood gene expression did not reveal any significant changes in leukocyte proportions between sALS patients and controls; however, sALS did demonstrate a relative increase in neutrophils (FDR = 0.052) (Figure S8).

### Peripheral blood prognostic biomarkers for sALS

#### Gene co‐expression modules were associated with sex and age

To identify whether modules of co‐expressed genes may correlate with sALS clinical features, we performed WGCNA on sALS cases (n = 96). Initial hierarchical clustering identified no clear clustering by clinical traits (Figure S9a) with downstream analysis identifying 24 modules, ranging in size from 25 to 2936 genes (Figure S9b). Correlation of module eigengenes and clinical traits identified four modules significantly associated with sex (brown4) or age at collection and age at disease onset (steelblue, darkolivegreen, cyan) (Figure S10a; Table S7). Of note, age at collection and age at disease onset were highly correlated (Pearson correlation coefficient = 0.974). No modules were identified to be significantly associated with the site of onset, disease duration or collection point in disease duration (Figure S9c).

GO biological processes and KEGG pathway enrichment analysis of module member genes identified key biological themes for all four significantly associated modules (Figure S11). The sex‐associated module (brown4, 25 genes) was most significantly enriched in genes involved in bacterial defence response. The three age‐associated modules were enriched in genes involved in morphogenesis (steelblue, 92 genes), B cell activation (darkolivegreen, 80 genes) and adaptive immune response (cyan, 778 genes). Of note, the cyan module also included enrichment for “regulation of muscle organ development” and “spinal cord development” GO terms. Given that both sex and age are not disease‐specific traits, we next examined whether the 24 identified gene co‐expression modules were conserved in the control cohort (n = 48). The module preservation test revealed that all modules were strongly preserved (Zsummary ≥10) in control gene expression data (Figure S10b), suggesting that the four modules identified to associate with sex and age are likely to be unrelated to disease status. Preservation median rank indicated that the sex‐associated brown4 module was best conserved between sALS and control data sets.

#### Predicted sALS immune cell composition was not reflective of the stage of disease progression

The ratio of neutrophils to CD16− (classical) monocytes has previously been reported to be both increased in ALS cases vs controls and to correlate with disease progression, specifically the revised amyotrophic lateral sclerosis functional rating scale (ALSFRS‐R) score [41]. While we could not specifically identify CD16− monocytes, we did observe a ∼20% increase in the mean neutrophil:monocyte ratio in sALS (Welch two‐sample *t*‐test *p*‐value = 0.0671). However, no significant association was observed between the collection point in the disease course and neutrophil:monocyte ratio (R^2^ = 0.026, *p* = 0.142). A regression analysis was similarly performed to determine whether the proportion of other cell types was associated with the collection point in disease duration. A decrease in mast cell proportion was significantly associated with collection point in disease course (Figure S12). A decrease in B cell and an increase in neutrophil proportions with collection point was observed; however, the relationships did not reach statistical significance (p = 0.053).

#### Peripheral blood gene expression profiles were not predictive of patient survival

sALS disease duration could not be accurately predicted from peripheral blood gene expression alone, with a maximum R^2^ score of 0.7393 and Pearson's correlation of 0.5670 when using feature set 3 (CPM for all 12,569 detected genes) (Figure 3C, Table S8). The mean prediction error (MAE) was 30.94 months or 56.53% deviated from the real values (MAPE). The analysis was repeated for the 84 deceased sALS cases with the addition of clinical traits (sex, age at collection, site of onset) to the regression model. Prediction of disease duration was only marginally improved, with a maximum R^2^ score of 0.7518 and Pearson's correlation of 0.4061, again using feature set 3 (MAE: 20.74 months, MAPE: 42.56%) (Figure 3D, Table S9).

### Peripheral blood sALS patient subgroup stratification biomarkers

#### Early‐ and late‐stage sALS patients were distinct subgroups when compared with control gene expression profiles

Given that the sALS cohort sample collection was distributed evenly across disease duration (Figure S2), we hypothesised that stratification of patients into those whose collection occurred early (“early‐stage sALS”) and late (“late‐stage sALS”) in the disease course may highlight discrete gene expression changes. First, each group (early‐stage and late‐stage) was compared relative to controls. Two hundred and seventy‐nine genes were identified as differentially expressed between late collection sALS and controls (Figure S13a), whereas only one differentially expressed gene was identified in early collection sALS vs controls (*C4BPA*). 28% (78/279) of differentially expressed genes identified between late collection sALS and controls were also identified in sALS–control analyses. Similar to the sALS–control analyses, GO and KEGG pathway analysis of genes differentially expressed between late collection sALS and controls, highlighted the enrichment of genes involved in immune and metabolic pathways (Figure S13b). Notably, unique stress (“response to immobilisation stress”) and muscle (“regulation of muscle system process”, “skeletal muscle organ development”) related pathways were also identified to be enriched in late collection sALS differentially expressed genes. Despite the observed differences when examined relative to controls, direct comparisons between early and late collection sALS identified no differentially expressed genes. Further, the correlation of the log2 fold changes between early and late collection sALS, each relative to controls, revealed significant positive correlations (Figure S13c), suggesting concordant gene expression changes. No significant differences in demographic, technical or clinical features were identified between controls and/or early and late collection sALS groups (Figure S14, Table S10).

#### Unsupervised clustering analysis identified patient subgroups with distinct gene expression profiles

**FIGURE 4:**
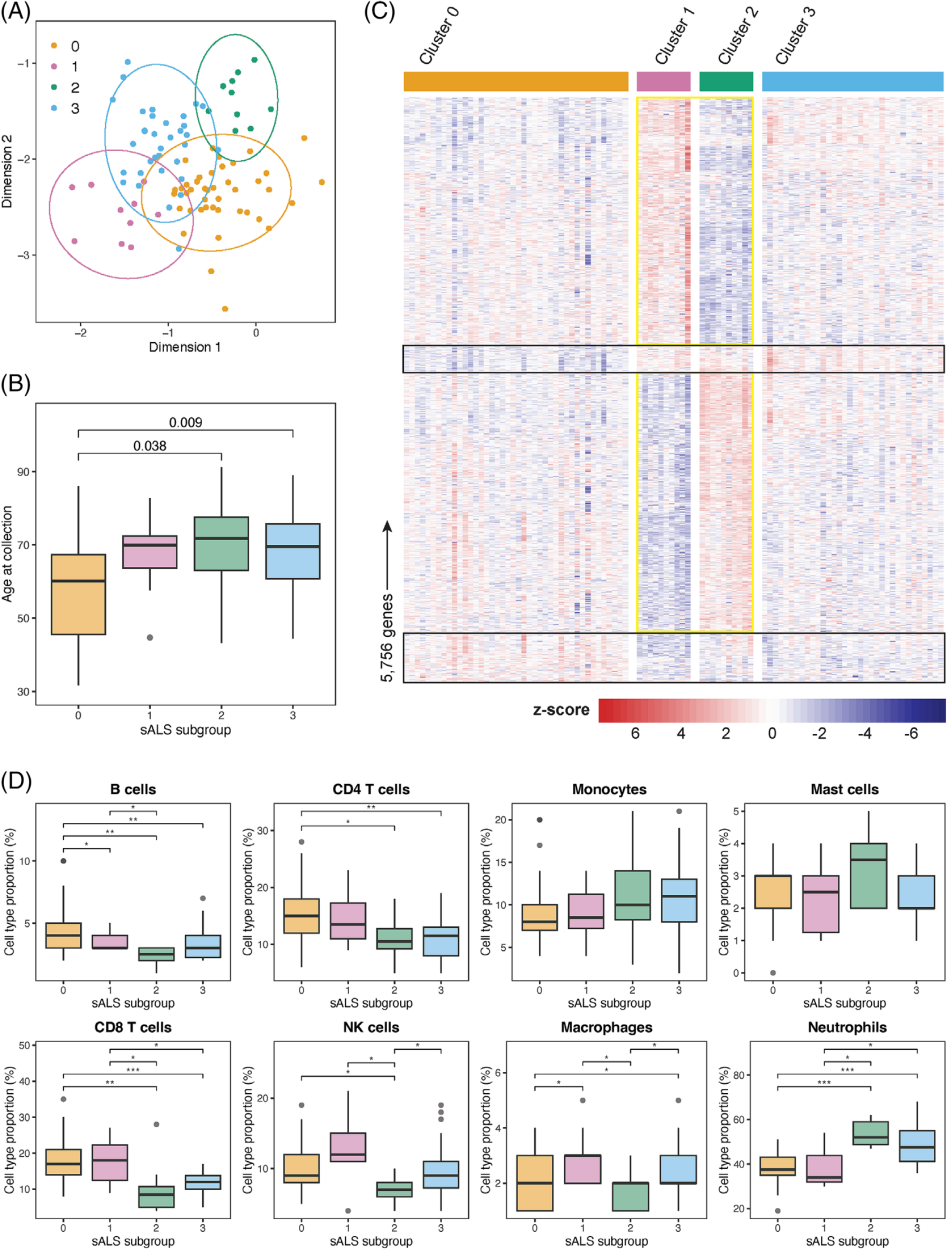
Unsupervised clustering analysis of blood gene expression data identified four sALS subgroups. (A) Cluster membership of the 96 sALS cases is visualised on a t‐SNE plot. Ellipses indicate 95% confidence intervals for each cluster. (B) Age at collection was identified to be significantly different between sALS subgroups (*p* = 0.0029, Kruskal–Wallis rank‐sum test). Significant *p*‐values (FDR < 0.05) from post hoc Wilcoxon rank‐sum exact tests are displayed. (C) Heatmap of 5756 genes identified to be significantly differentially expressed between sALS subgroups using the overlap method. Genes defining cluster 0 relative to all other clusters are highlighted by the black boxes. Genes defining clusters 1 and 2 are highlighted by the yellow boxes. Gene counts are z‐score normalised. (D) Proportions of eight major leukocytes were variable between sALS subgroups. Cell type proportions identified to be significantly different between sALS subgroups are indicated by asterisks (post hoc Wilcoxon rank‐sum exact test with Benjamini‐Hochberg correction; *p < 0.05, **p < 0.001, ***p < 0.0001). Cell types were deconvolved from bulk blood RNA‐seq using CIBERSORTx and the LM22 signature matrix. Dendritic cells, eosinophils, γδT cells and plasma cells made up <1% of the total cell proportion across subgroups so were excluded from the analysis.

**FIGURE 5:**
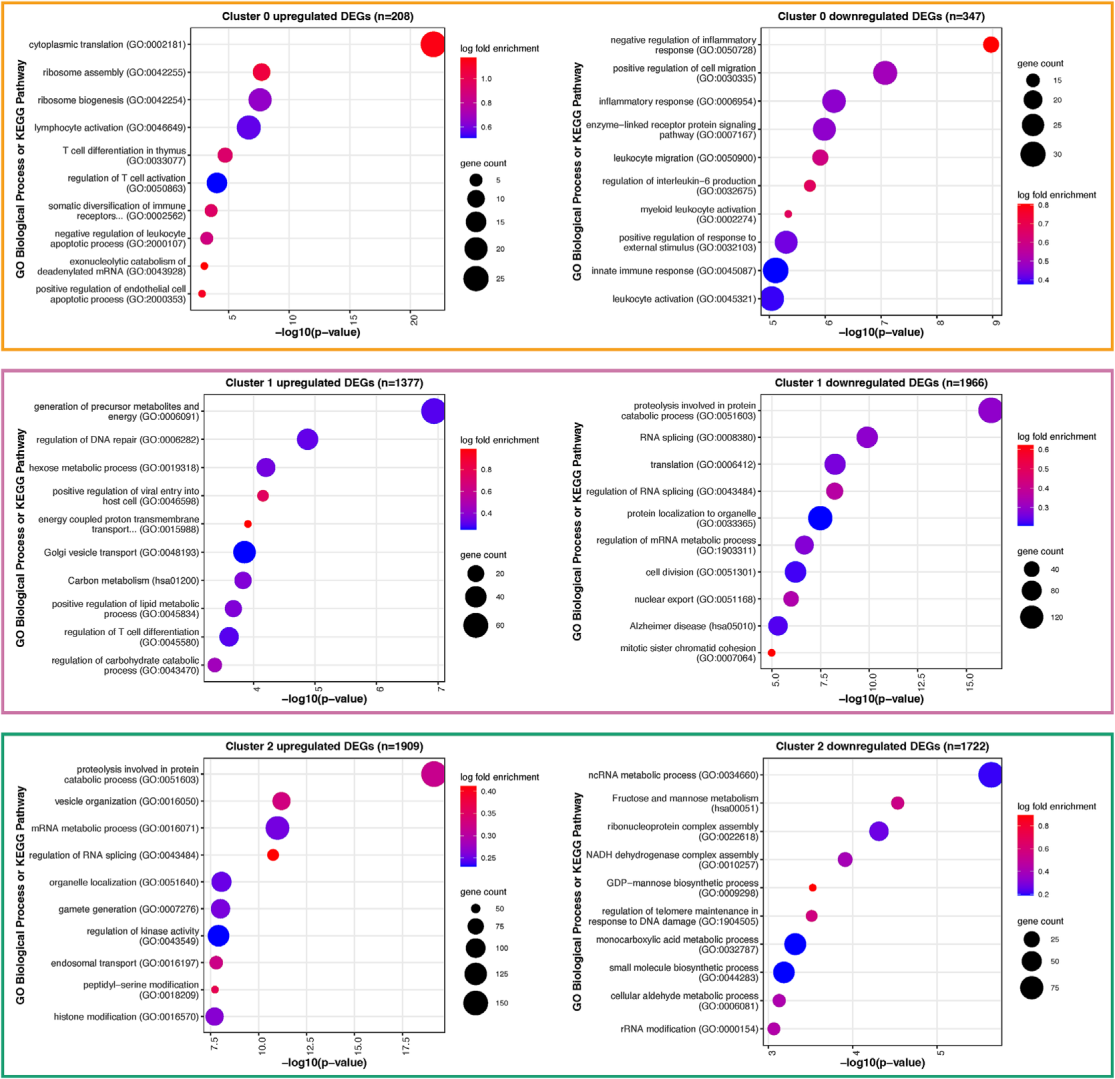
GO biological processes and KEGG pathways enriched for genes that were significantly upregulated or downregulated in sALS subgroups. The number of differentially expressed genes (DEGs) for each cluster is indicated by n value. Enrichment analysis was not performed on cluster 3 because of a low number of overlapping DEGs. The most representative member (lowest p‐value) of the top ten GO term clusters is displayed. Gene count indicates the number of DEGs in each pathway. Log2 fold change indicates enrichment of member genes in DEGs vs all detected genes. A complete list of enriched pathways can be found in Tables S13a‐f.

ALS is a phenotypically heterogeneous disease and it has previously been suggested that patient subgroups can be identified using gene expression data from both post‐mortem brain [42] and blood [11]. To determine whether patient subgroups could be identified among our 96 sALS cases, we applied an unsupervised clustering approach to normalised gene counts. K‐means clustering identified four sALS patient subgroups containing 42 (cluster 0, yellow), 34 (cluster 3, blue) or 10 (clusters 1 and 2, pink and green, respectively) individuals (Figure 4A). Age at collection and age at disease onset were the only two sample features identified to be significantly different between the four patient subgroups (Figure 4B, Figure S15, Table S11). Specifically, cluster 0 was identified to contain individuals with a younger age at collection and age at disease onset. Differential gene expression analysis between clusters identified 5756 subgroup‐defining genes (Table S12a), with the vast majority of these identified as differentially expressed in cluster 1 and cluster 2 relative to all other clusters (Figure S16). Heatmap visualisation of these genes highlighted clear gene expression signatures for cluster 1 and cluster 2, with fewer genes defining cluster 0 relative to all other clusters (Figure 4C). GO biological pathway and KEGG pathway enrichment of the subgroup defining genes identified “proteolysis involved in protein catabolic process” as the most significantly enriched pathway among cluster 1 downregulated genes and cluster 2 upregulated genes (Figure 5, Tables S13a‐f). Other pathways enriched among cluster 1 and cluster 2 defining genes included metabolic pathways, RNA splicing and regulation, cellular transport and DNA damage response, often in opposing expression directions. For cluster 0, translation and adaptive immune response were significantly enriched among upregulated genes while inflammatory and innate immune response was enriched among downregulated genes (Figure 5).

While many differentially expressed genes were identified for cluster 3 relative to each of the other three clusters, few subgroup‐defining genes were identified because of a lack of overlap between the three comparisons (Figure S16). Given that the cluster 3 expression profile was situated between the opposing clusters 1 and 2 (Figure 4A), we next considered the genes differentially expressed between the two largest patient clusters, cluster 0 and cluster 3. Heatmap visualisation of the 1355 genes (Table S12b) identified to be significantly differentially expressed between clusters 0 and 3 identified a clear expression difference between the sALS subgroups (Figure S17a). Top biological pathways enriched among genes upregulated in cluster 3 relative to cluster 0 included humoral and innate immune responses (Figure S17b; Tables S13g‐h). In contrast, genes downregulated in cluster 3 relative to cluster 0 were primarily involved in the adaptive immune response, including B and T cell activation. Further supporting this finding, leukocyte proportions predicted by cell type deconvolution identified a significant reduction in lymphocytes (B cells, CD4 + and CD8 + T cells) and an increase in select innate immune cells (macrophages and neutrophils) in cluster 3 patients compared with those in cluster 0 (Figure 4D).

## DISCUSSION

We sought to determine the utility of peripheral blood RNA‐seq for the discovery of diagnostic, prognostic and subgroup stratification biomarkers in a large cohort of sALS patients and controls. Whole blood gene expression profiles accurately distinguished sALS cases from controls and captured pathway‐level alterations that may reflect pathophysiological mechanisms and/or peripheral effects of disease. Gene expression data was less informative when attempting to derive prognostic biomarkers and further investigation of carefully curated and longitudinal cohorts is warranted. Finally, we demonstrated the presence of patient subgroups with distinct gene expression alterations, suggesting that whole blood RNA‐seq may be useful for patient stratification.

### Peripheral blood RNA‐seq distinguished sALS patients from controls

We report a classification model that distinguished sALS patients from controls with an accuracy of 78% (sensitivity: 79%, specificity: 75%) using the expression of 20 genes. In a previous study, Swindell et al. reported a support vector machine that distinguished ALS patients from controls, and disease mimics with 87% accuracy (sensitivity: 86%, specificity: 87%) using the expression of 850 genes. This may represent the upper limit in the diagnostic accuracy for models derived from whole blood gene expression, as patient heterogeneity and pathophysiological overlap with disease mimics may prevent further improvements to accuracy [43]. Higher resolution technologies, including single‐cell RNA‐seq, may provide the next elevation in diagnostic accuracy. Notably, a recent study reported an ALS–control classification model with an accuracy of 93% (sensitivity: 92%, specificity: 93%) using 60 gene markers derived from single‐cell RNA‐seq of ALS peripheral blood mononuclear cells [44]. When tested in an independent ALS–control whole blood RNA‐seq data set (n = 60), the accuracy of our classification model was reduced to 63.3%, falling short of the LOO cross‐validation performance achieved by the original training data set. Lack of standardisation in RNA preparation and sequencing methodologies between laboratories are likely contributors to varying model performance and presents a challenge to biomarker discovery as recognised by previous studies [3].

Our study identified 245 differentially expressed genes between sALS cases and controls, equivalent to 1.9% of detected genes. Previous RNA‐seq and microarray studies that examined whole blood gene expression have described less subtle alterations, reporting ≥10% of genes as differentially expressed [2, 3, 4, 11]. Of the 245 differentially expressed genes identified here, only eight upregulated genes (*CASP4, CA4, CYSTM1, FPR1, MSRB1, RGL4, ST6GALNAC2, VNN1*) and one downregulated gene (*RPL7L1*) were shown to be differentially expressed in both of the previously reported whole blood gene expression studies, including the RNA‐seq study of 6 sALS patients and 5 controls [4] and the largest whole blood sALS–control gene expression microarray study to date (397 ALS patients, 645 controls) [11]. This lack of gene‐level reproducibility was likely due to technical and analytical differences including sample collection methods, the use of different gene expression platforms and data pre‐processing approaches. Nevertheless, pathway‐level analysis of differentially expressed genes highlighted concordant alterations in biological processes including enrichment of genes involved in metabolic pathways, transcription regulation, immune response and apoptosis [3, 4, 11]. The ferroptosis signalling pathway, an iron‐dependent form of cell death recently implicated in ALS motor neuron loss [45], was the top biological pathway predicted by IPA to be activated in sALS peripheral blood. Interestingly, two blood‐based markers of ferroptosis, 4‐hydroxy‐2‐nonenal (a product of lipid peroxidation) and ferritin (an indicator of brain iron status) have been demonstrated to distinguish fast and slow disease progressors early in the disease and to associate with ALSFRS‐R decline [46]. Molecules predicted by IPA to be activated in sALS cases were notably dominated by markers of peripheral inflammation, many of which have been reported as upregulated in ALS, albeit with inconsistent findings [47]. The diagnostic and prognostic potential of inflammatory mediators requires further investigation with the lack of reliable markers to date potentially confounded by variable ALS patient immune phenotypes, a feature noted in the present study.

RNA‐seq offers the ability to examine transcript‐level alterations in gene expression. Here, we observed differential transcript usage, where the ratio of gene transcripts in an individual shifted, for 13 genes from a range of biological pathways. These included genes that encode a component of the SCF ubiquitination complex (*FBXO31)*, an enzyme involved in fatty acid oxidation (*HADH*) and a member of the *KCTD* family (*KCTD7*), mutations in which have been linked to several neurological disorders [48]. It was possible that the observed variance in transcript usage was a consequence of different immune cell composition. While no significant differences in ALS whole blood cell type proportions were identified in this study, shifts were observed that agreed with previous studies that employed direct cell type quantification [41]. Nevertheless, these and other transcript‐level alterations warrant further investigation as candidate biomarkers, as supported by the recent reports of *STMN2* [49] and *UNC13A* [50, 51] in ALS post‐mortem tissue and the alternative splicing events seen in the peripheral blood of other neurological disorders [52, 53].

### Whole blood RNA‐seq had limited capacity for ALS prognostic biomarker discovery

WGCNA has previously been applied to ALS–control peripheral blood microarray data to identify gene co‐expression modules associated with disease status [2, 54]. Our application of WGCNA did not identify any modules that were predictive of clinical traits in the sALS patients (site of onset, disease duration, sample collection point) but rather identified modules predictive of patient sex and age, all of which were preserved in the control population. We were also unable to produce an accurate regression model to predict disease duration, with the best‐performing model demonstrating a mean prediction error of almost 2 years (43% deviated from true values). Swindell et al. took an alternate approach and generated a Cox proportional hazards model using the same clinical features (sex, age, site of onset) and a 61 gene panel, reporting a concordance index of 0.74 [11]. Samples in the current study were collected at different stages throughout disease duration, which potentially confounded prognostic potential although we observed no significant differences in gene expression between patients whose collection occurred early vs late in disease. We also did not observe any clear shifts in immune cell proportions throughout the disease duration that may have been predictive of ALS patient progression. Together, our findings suggest that whole blood RNA‐seq has limited use for monitoring ALS progression; however, patient‐matched longitudinal data sets will be better suited to prognostic biomarker discovery, especially considering the observed inter‐patient variability in gene expression profiles.

### Peripheral blood gene expression profiles distinguished ALS patient subgroups

Machine learning identified four patient subgroups with distinct blood gene expression profiles in our cohort of 96 sALS patients. Two subgroups consisted of 10 patients each and demonstrated clearly opposing expression of genes involved in proteolysis, RNA splicing and regulation, cellular transport, and DNA damage response. While clinical features did not explain the observed patient subgroups, the largest subgroup of 42 patients (cluster 0) did consist of significantly younger patients. Interestingly, differences in immune response were evident between this patient subgroup and the second largest of 34 patients (cluster 1), whereby their differentially expressed genes demonstrated opposing enrichment for the innate and adaptive immune responses. Notably, Swindell et al. reported two patient subgroups with either lymphoid or myeloid gene expression signatures [11], a finding further supported by our observed differences in innate and adaptive immune cell proportions. It is unclear whether the identified patient subgroups are consistent with pathophysiological alterations or represent peripheral responses to disease. Further investigation is required to determine whether stratifying patients by the identified groups would be of clinical value. RNA‐seq of matched CNS and peripheral blood samples would be valuable in identifying clinically relevant and accessible biomarkers for patient stratification.

## CONCLUSIONS

We present a comprehensive analysis of an independent ALS–control peripheral blood RNA‐seq cohort and highlight the presence of distinct patient subgroups. The observed heterogeneity in the ALS blood gene expression profiles complicates diagnostic and prognostic biomarker discovery and future RNA‐seq studies should employ longitudinal samples and/or CNS–blood‐matched cohorts.

## AUTHOR CONTRIBUTIONS

**Natalie Grima**: Conceptualization, Formal analysis, Funding acquisition, Investigation, Methodology, Visualisation, Writing—original draft preparation, Writing—review and editing. **Sidong Liu**: Formal analysis, Methodology, Writing—review and editing. **Dean Southwood**: Formal analysis, Investigation, Writing—review and editing. **Lyndal Henden**: Formal analysis, Writing—review and editing. **Andrew Smith**: Formal analysis, Writing—review and editing. **Albert Lee**: Formal analysis, Resources, Writing—review and editing. **Dominic B. Rowe**: Resources, Writing—review and editing. **Susan D'Silva**: Data curation, Writing—review and editing. **Ian P. Blair**: Funding acquisition, Resources, Supervision, Writing—review and editing. **Kelly L. Williams**: Conceptualization, Data curation, Funding acquisition, Resources, Supervision, Writing—review and editing.

## CONFLICT OF INTEREST STATEMENT

The authors declare no competing interests.

### PEER REVIEW

The peer review history for this article is available at https://www.webofscience.com/api/gateway/wos/peer-review/10.1111/nan.12943.

### CODE AVAILABILITY STATEMENT

Code written in R is available in R Markdown workbooks in a GitLab repository: https://gitlab.com/mq-mnd/grp_williams/sals_blood_rnaseq.

## ETHICS STATEMENT

All participants provided informed written consent for research participation as approved by the human research ethics committee of Macquarie University (520211013428875).

## Supporting information


Supplementary Materials
Figure S1 Confounder identification and adjustment
Figure S2 sALS sample collection is distributed across disease duration
Figure S3 Comparison of gene count transformations for unsupervised learning
Figure S4 Preparation of the independent blood RNA‐seq data set for validation of the classification model
Figure S5 Comparison of sample features between sALS (n = 96) and control (n = 48) groups
Figure S6 Two sample t‐test power curve for 96 sALS and 48 controls
Figure S7 Visual of 15 genes identified to have differential transcript usage between sALS patients and controls
Figure S8 Proportions of 12 major leukocytes in sALS vs control peripheral blood
Figure S9 sALS patient clustering, module identification and association with clinical traits for Weighted Gene Co‐expression Network Analysis (WGCNA)
Figure S10 Weighted Gene Co‐expression Network Analysis (WGCNA) of sALS (n = 96) identified four modules associated with sex, age at collection or age at disease onset
Figure S11 GObiological processes and KEGG pathways enriched in the four co‐expression modules associated with sex (brown4) or age at collection and age at disease onset (steelblue, darkolivegreen, cyan)
Figure S12 The percentage of leukocytes in the blood of sALS patients against the stage in disease duration at which blood was collected
Figure S13 Differentially expressed genes were identified for late‐stage but not early‐stage sALS patients relative to controls
Figure S14 Comparison of sample features between controls (n = 48) and early‐stage (n = 28) or late‐stage (n = 28) sALS patients
Figure S15 Comparison of clinical features between sALS patient subgroups
Figure S16 Venn diagrams highlighting the overlap in differentially expressed genes between each sALS subgroup comparison
Figure S17 Differentially expressed genes identified between sALS cluster 0 and cluster 3 are enriched for immune related pathways
Table S2 Statistical results from comparison of sample features between sALS (n = 96) and control (n = 48) groups.
Table S6 The top 20 genes identified for the classification (sALS and controls) model using the Leave‐One‐Out (LOO) strategy
Table S8 The top 20 genes identified for prediction of disease duration regression model using the Leave‐One‐Out (LOO) strategy and gene expression data as input
Table S9 The top 20 genes identified for prediction of disease duration regression model using the Leave‐One‐Out (LOO) strategy, and gene expression and clinical data as input
Table S10 Statistical results from comparison of sample features between controls (n = 48) and early‐stage (n = 28) or late‐stage (n = 28) sALS patients
Table S11 Statistical results from comparison of sample features between four sALS subgroups identified by clustering analysis

Supplementary Tables
Table S1. Complete demographic, clinical and technical sample information for sALS (n = 96) and control (n = 48) peripheral blood RNA‐seq cohort.
Table S3. Differential gene expression results for sALS (n = 96) vs controls (n = 48).
Table S4. Metascape Gene Ontology (GO) and Kyoto Encyclopedia of Genes and Genomes (KEGG) pathway enrichment analysis result for sALS–control differentially expressed genes.
Table S5. Complete Ingenuity Pathway Analysis result for sALS–control differentially expressed genes.
Table S7. Genes belonging to four co‐expression modules significantly associated with sex or age in sALS cases as identified by WGCNA.
Table S12a. List of differentially expressed genes defining sALS subgroups, their cluster membership and direction of gene expression change.
Table S12b. List of differentially expressed genes identified in sALS cluster 3 relative to sALS cluster 0 (padj.BH < 0.05 and abs(logFC) > log2(1.5)).
Table S13a. Metascape Gene Ontology (GO) and Kyoto Encyclopedia of Genes and Genomes (KEGG) pathway enrichment analysis result for sALS subgroup differentially expressed genes.
Table S13b. Metascape Gene Ontology (GO) and Kyoto Encyclopedia of Genes and Genomes (KEGG) pathway enrichment analysis result for sALS subgroup differentially expressed genes.
Table S13c. Metascape Gene Ontology (GO) and Kyoto Encyclopedia of Genes and Genomes (KEGG) pathway enrichment analysis result for sALS subgroup differentially expressed genes.
Table S13d. Metascape Gene Ontology (GO) and Kyoto Encyclopedia of Genes and Genomes (KEGG) pathway enrichment analysis result for sALS subgroup differentially expressed genes.
Table S13e. Metascape Gene Ontology (GO) and Kyoto Encyclopedia of Genes and Genomes (KEGG) pathway enrichment analysis result for sALS subgroup differentially expressed genes.
Table S13f. Metascape Gene Ontology (GO) and Kyoto Encyclopedia of Genes and Genomes (KEGG) pathway enrichment analysis result for sALS subgroup differentially expressed genes.
Table S13g. Metascape Gene Ontology (GO) and Kyoto Encyclopedia of Genes and Genomes (KEGG) pathway enrichment analysis result for sALS subgroup differentially expressed genes.
Table S13h. Metascape Gene Ontology (GO) and Kyoto Encyclopedia of Genes and Genomes (KEGG) pathway enrichment analysis result for sALS subgroup differentially expressed genes.

## Data Availability

Raw FASTQ files, raw gene counts and corresponding offset matrix are available under NCBI Gene Expression Omnibus (GEO) accession GSE234297.
