# Supplementary material for: RNA sequencing of peripheral blood in amyotrophic lateral sclerosis reveals distinct molecular subtypes: Considerations for biomarker discovery
Source: Neuropathol Appl Neurobiol. 2023 Nov 7;49(6):e12943. doi: 10.1111/nan.12943 (PMC10946588; doi:10.1111/nan.12943)
Supplement: Supplementary file 1 — Supplementary Materials Figure S1 Confounder identification and adjustment Figure S2 sALS sample collection is distributed across disease duration Figure S3 Comparison of gene count transformations for unsupervised learning Figure S4 Preparation of the independent blood RNA‐seq data set for validation of the classification model Figure S5 Comparison of sample features between sALS (n = 96) and control (n = 48) groups Figure S6 Two sample t‐test power curve for 96 sALS and 48 controls Figure S7 Visual of 15 genes identified to have differential transcript usage between sALS patients and controls Figure S8 Proportions of 12 major leukocytes in sALS vs control peripheral blood Figure S9 sALS patient clustering, module identification and association with clinical traits for Weighted Gene Co‐expression Network Analysis (WGCNA) Figure S10 Weighted Gene Co‐expression Network Analysis (WGCNA) of sALS (n = 96) identified four modules associated with sex, age at collection or age at disease onset Figure S11 GObiological processes and KEGG pathways enriched in the four co‐expression modules associated with sex (brown4) or age at collection and age at disease onset (steelblue, darkolivegreen, cyan) Figure S12 The percentage of leukocytes in the blood of sALS patients against the stage in disease duration at which blood was collected Figure S13 Differentially expressed genes were identified for late‐stage but not early‐stage sALS patients relative to controls Figure S14 Comparison of sample features between controls (n = 48) and early‐stage (n = 28) or late‐stage (n = 28) sALS patients Figure S15 Comparison of clinical features between sALS patient subgroups Figure S16 Venn diagrams highlighting the overlap in differentially expressed genes between each sALS subgroup comparison Figure S17 Differentially expressed genes identified between sALS cluster 0 and cluster 3 are enriched for immune related pathways Table S2 Statistical results from comparison of sample features between s [file NAN-49-0-s001.pdf]

Supplementary Materials for

RNA sequencing of peripheral blood in amyotrophic lateral sclerosis reveals distinct molecular subtypes: considerations for biomarker discovery

Natalie Grima, Sidong Liu, Dean Southwood, Lyndal Henden, Andrew Smith, Albert Lee, Dominic B. Rowe, Susan D'Silva, Ian P. Blair and Kelly L. Williams

*Corresponding Author. Email: kelly.williams@mq.edu.au

This PDF file includes:

Figs. S1 to S17
Tables S2, S6, S8-S11
Supplementary Methods

Supplementary Figs.

S1 Confounder identification and adjustment . . . 3
S2 sALS sample collection is distributed across disease duration . . . 4
S3 Comparison of gene count transformations for unsupervised learning . . . 5
S4 Preparation of the independent blood RNA-seq data set for validation of the classification model . . . 6
S5 Comparison of sample features between sALS (n=96) and control (n=48) groups 7
S6 Two sample t-test power curve for 96 sALS and 48 controls . . . 8
S7 Visual of 15 genes identified to have differential transcript usage between sALS patients and controls . . . 9
S8 Proportions of 12 major leukocytes in sALS versus control peripheral blood . . 24
S9 sALS patient clustering, module identification and association with clinical traits for Weighted Gene Co-expression Network Analysis (WGCNA) . . . 25
S10 Weighted Gene Co-expression Network Analysis (WGCNA) of sALS (n=96) identified four modules associated with sex, age at collection or age at disease onset . . . 26
S11 GO biological processes and KEGG pathways enriched in the four co-expression modules associated with sex (brown4) or age at collection and age at disease onset (steelblue, darkolivegreen, cyan) . . . 27
S12 The percentage of leukocytes in the blood of sALS patients against the stage in disease duration at which blood was collected . . . 28
S13 Differentially expressed genes were identified for late-stage but not early-stage sALS patients relative to controls . . . 29
S14 Comparison of sample features between controls (n=48) and early-stage (n=28) or late-stage (n=28) sALS patients . . . 30
S15 Comparison of clinical features between sALS patient subgroups . . . 31
S16 Venn diagrams highlighting the overlap in differentially expressed genes between each sALS subgroup comparison . . . 32
S17 Differentially expressed genes identified between sALS cluster 0 and cluster 3 are enriched for immune related pathways . . . 33

Supplementary Tables

S2 Statistical results from comparison of sample features between sALS (n=96) and control (n=48) groups. . . . 34
S6 The top 20 genes identified for the classification (sALS and controls) model using the Leave-One-Out (LOO) strategy . . . 35
S8 The top 20 genes identified for prediction of disease duration regression model using the Leave-One-Out (LOO) strategy and gene expression data as input . . 36
S9 The top 20 genes identified for prediction of disease duration regression model using the Leave-One-Out (LOO) strategy, and gene expression and clinical data as input . . . 37
S10 Statistical results from comparison of sample features between controls (n=48) and early-stage (n=28) or late-stage (n=28) sALS patients . . . 38
S11 Statistical results from comparison of sample features between four sALS subgroups identified by clustering analysis . . . 39

Supplementary Methods

Supplementary Figs.

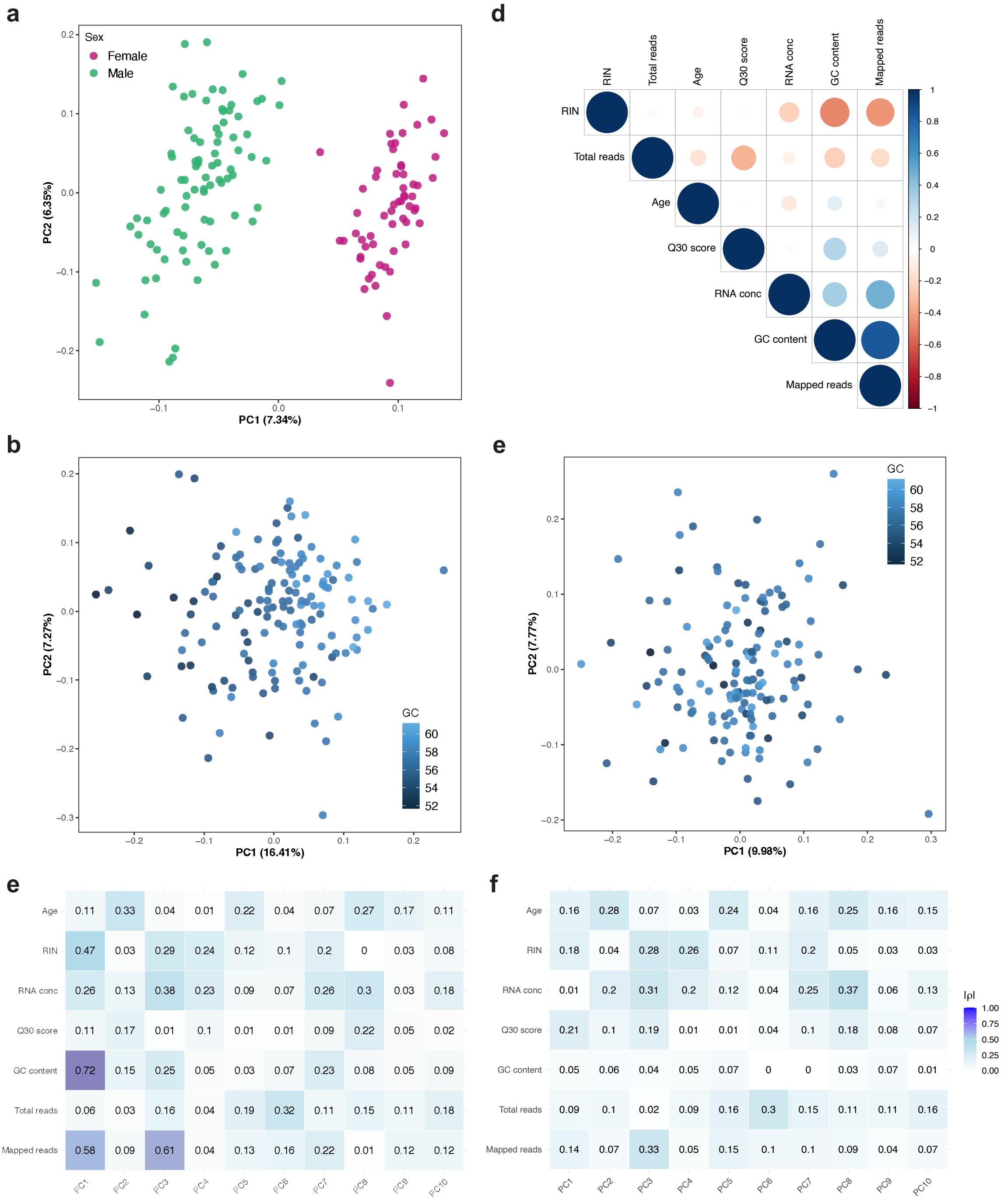

Fig. S1: Confounder identification and adjustment. a Principal component analysis (PCA) of the top 500 variable genes highlighted sex separation across PC1. PCA of all expressed genes highlighted correlation between PC1 and sample GC content shown **b** visually and **c** by Spearman's rank correlation coefficient (ρ). **d** Pearson correlation between continuous variables. Correction for GC content via limma::removeBatchEffect reduced correlations between technical variables and the top 10 PCs shown **e** visually and **f** by Spearman's rank correlation coefficient.

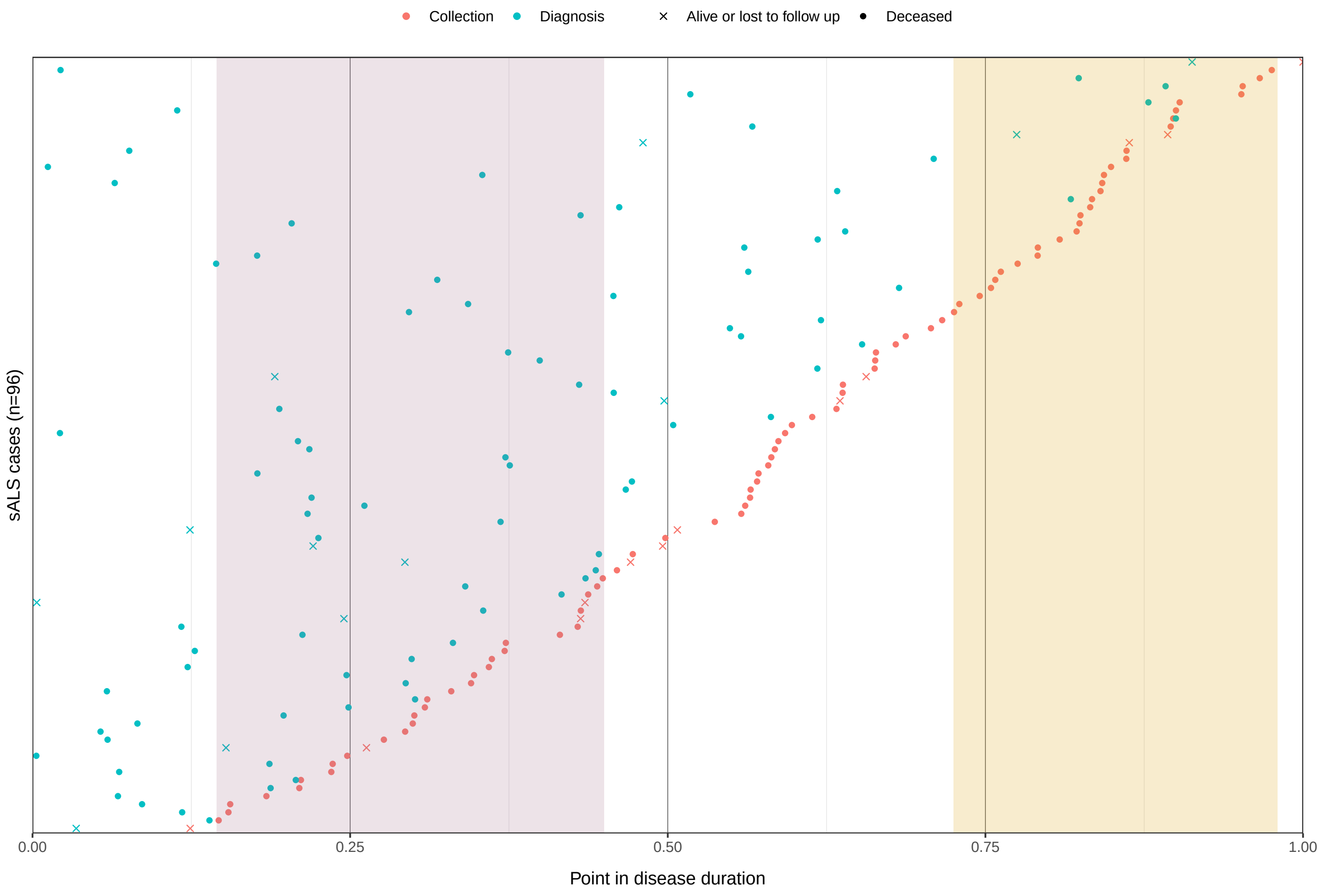

Fig. S2: sALS sample collection is distributed across disease duration. Collection (red) and diagnosis (blue) point in disease duration of 96 sALS patient peripheral blood samples. Along the x-axis, 0 represents disease onset and 1 represents death. The ratio between 0-1 is calculated as: (date of sample collection OR diagnosis – date of disease onset) / (date of death – date of disease onset). 12 patients are alive or lost to follow up (cross) and 84 patients are confirmed deceased (circle). Early- and late-stage collection groups, defined as the 33rd and 66th percentiles, are highlighted in purple and yellow, respectively.

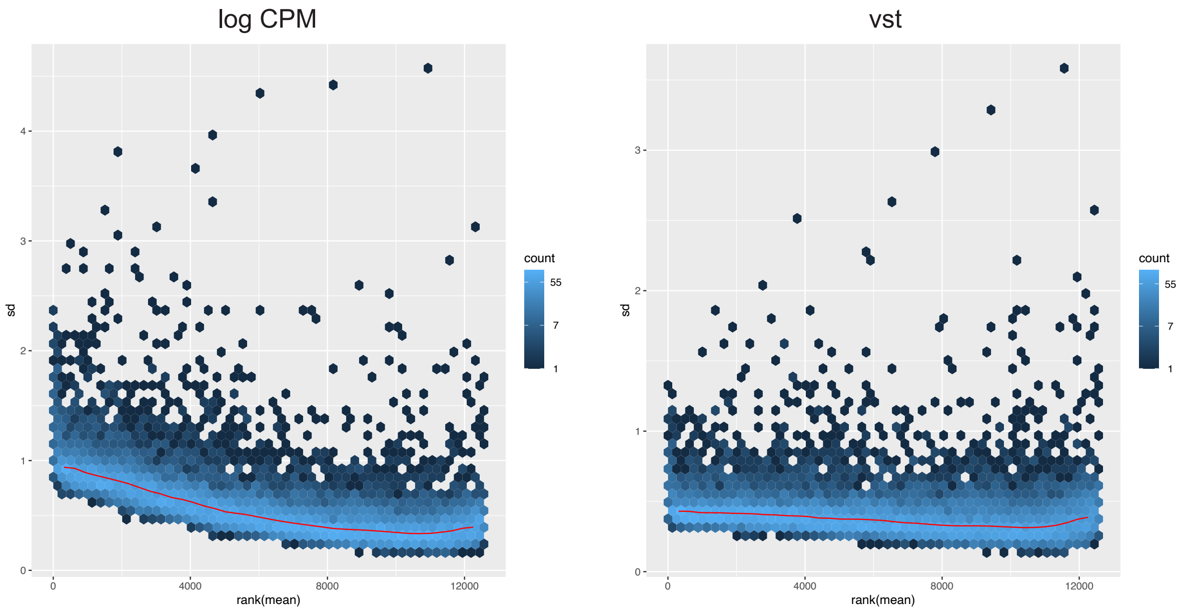

Fig. S3: Comparison of gene count transformations for unsupervised learning. CPM, log counts per million; vst, variance stabilising transformation; sd, standard deviation.

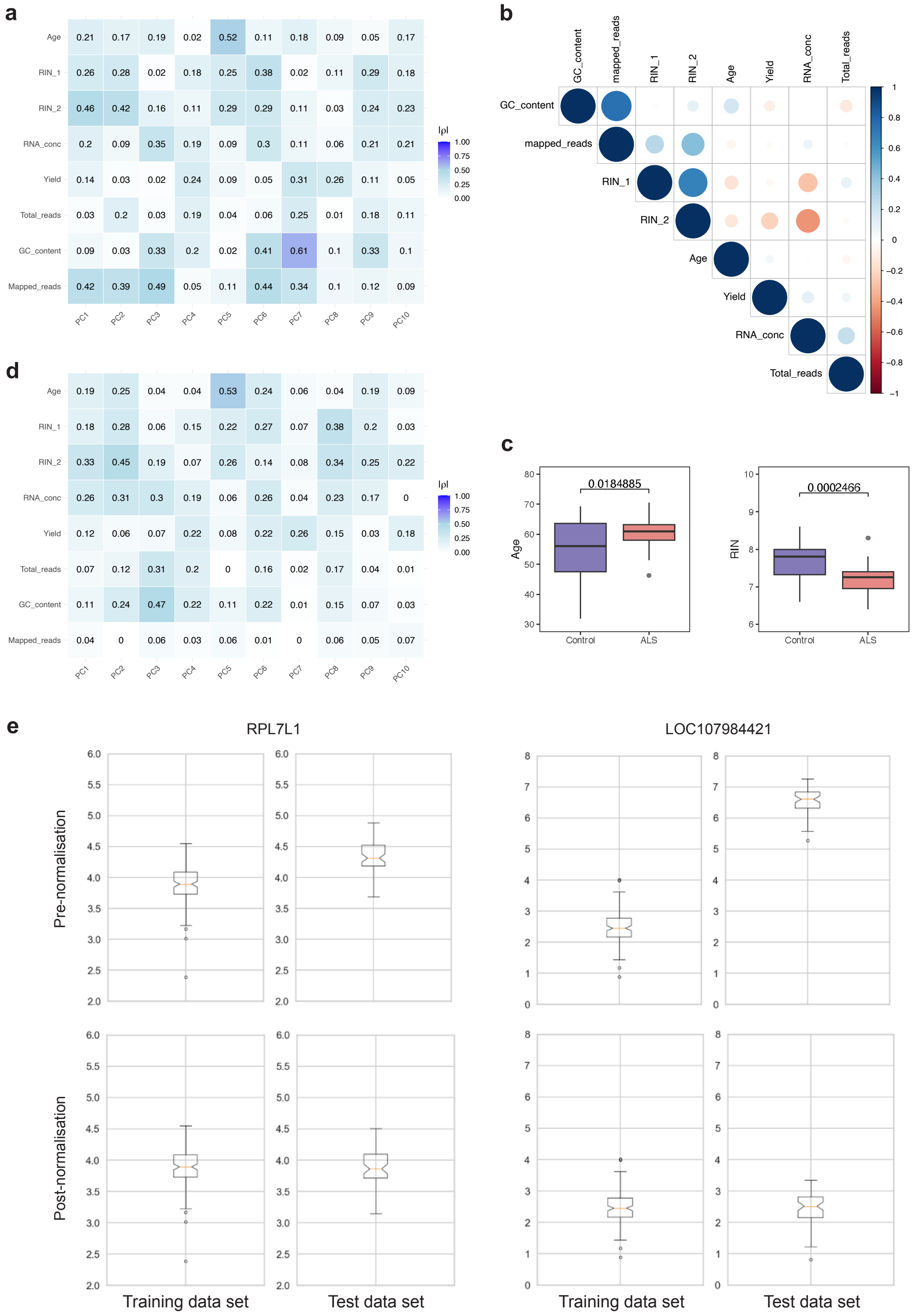

Fig. S4: Preparation of the independent blood RNA-seq data set for validation of the classification model. a Spearman's rank correlation coefficient of the top 10 PCs against available covariates. RIN_1 is RIN before shipping. **b** Pearson correlation between continuous variables. **c** Age and RIN are significantly different between ALS and control groups. P-value is from Welch Two Sample t-test. **d** Spearman's rank correlation coefficient of the top 10 PCs against available covariates after correcting for Salmon mapped reads percentage via limma::removeBatchEffect. **e** Distribution of the top two features (genes) from the classification model in the training (n=144) versus test (n=60) data sets, pre- and post-normalisation.

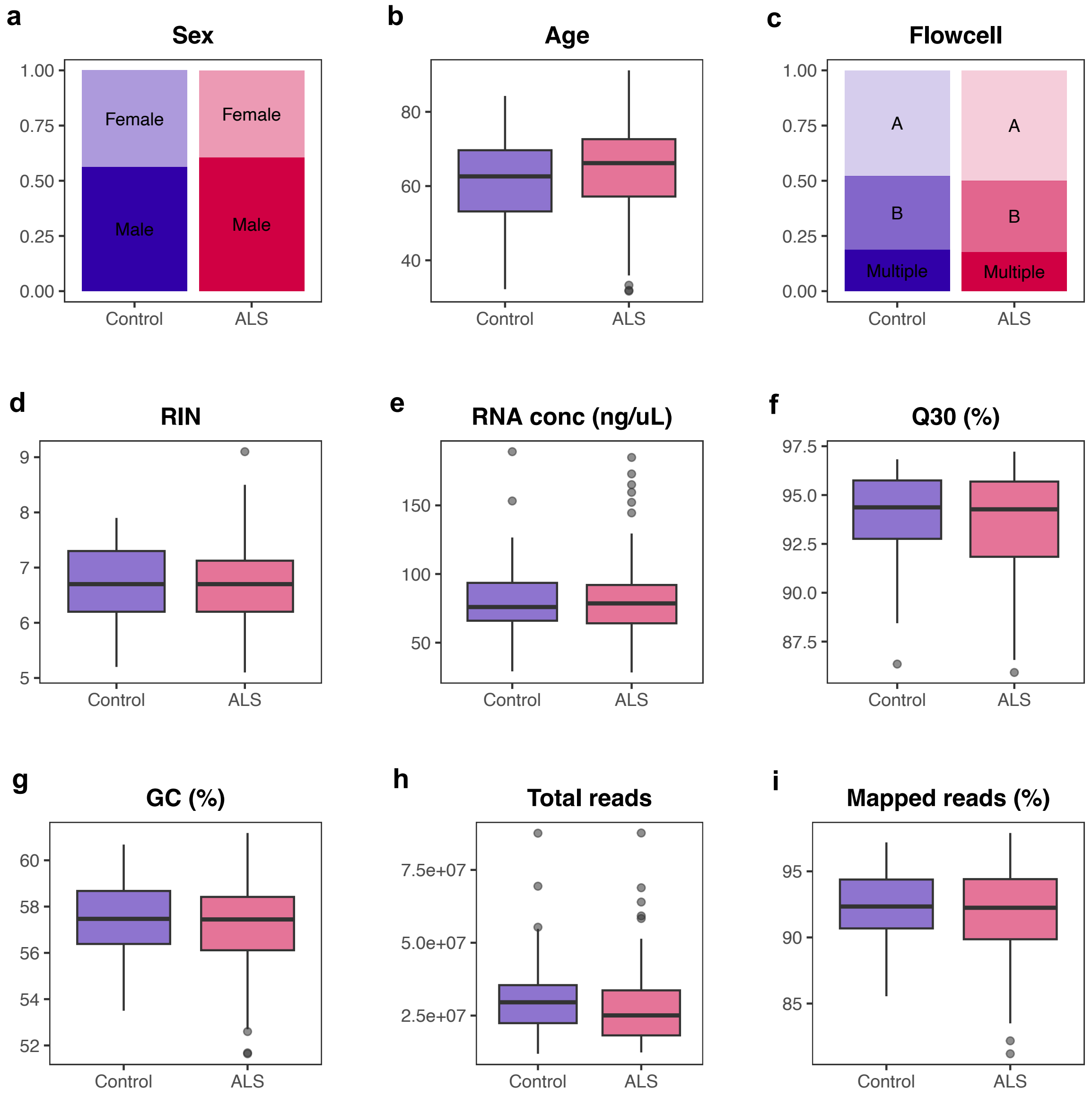

Fig. S5: Comparison of sample features between sALS (n=96) and control (n=48) groups. **a-i** No features were significantly different between control and sALS groups. Results of statistical comparisons can be found in Table S2. Flowcell refers to the specific flowcell that samples were run on for RNA sequencing: "A" and "B" each indicate a distinct flowcell while "multiple" indicates that the sample was run across both flow cells (**c**). RIN, RNA integrity number (**d**). Q30%, ratio of bases that have phred quality score greater than or equal to 30 (**f**). Sequencing metrics are all post-trimming (**f-i**).

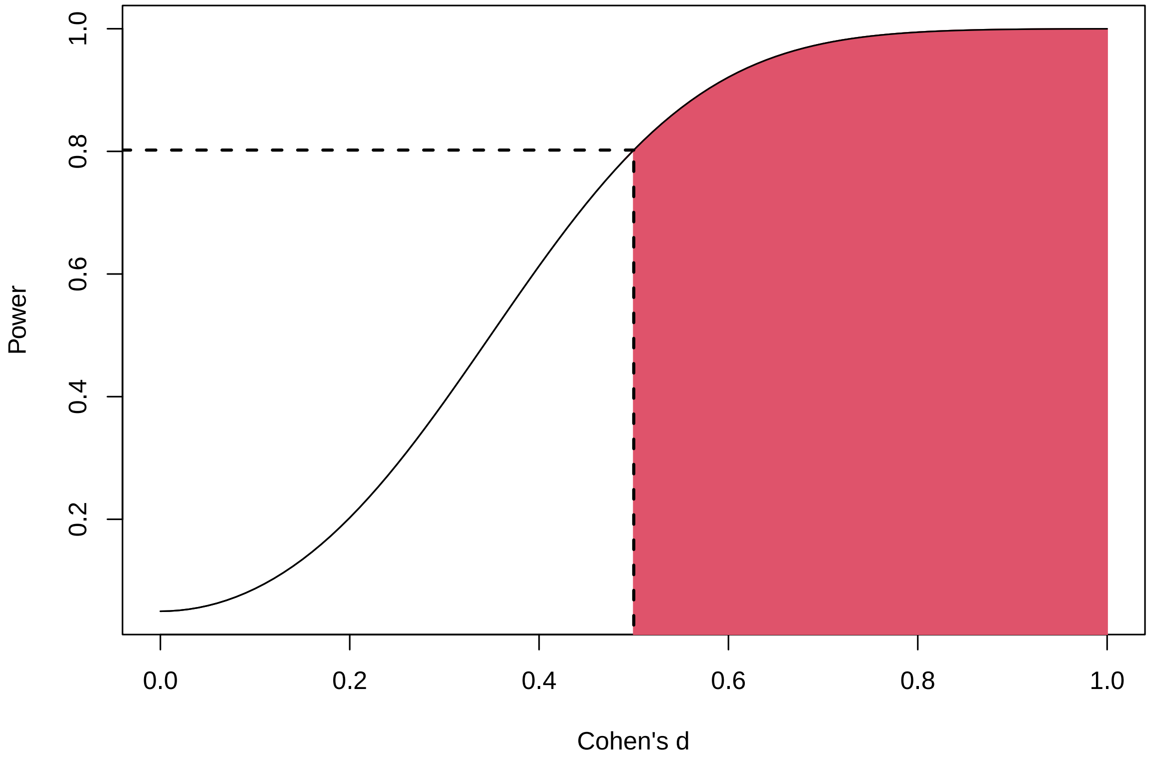

Fig. S6: Two sample t-test power curve for 96 sALS and 48 controls. At 80% power this cohort can detect medium to large (Cohen's d ≥ 0.5) effect sizes (highlighted in red).

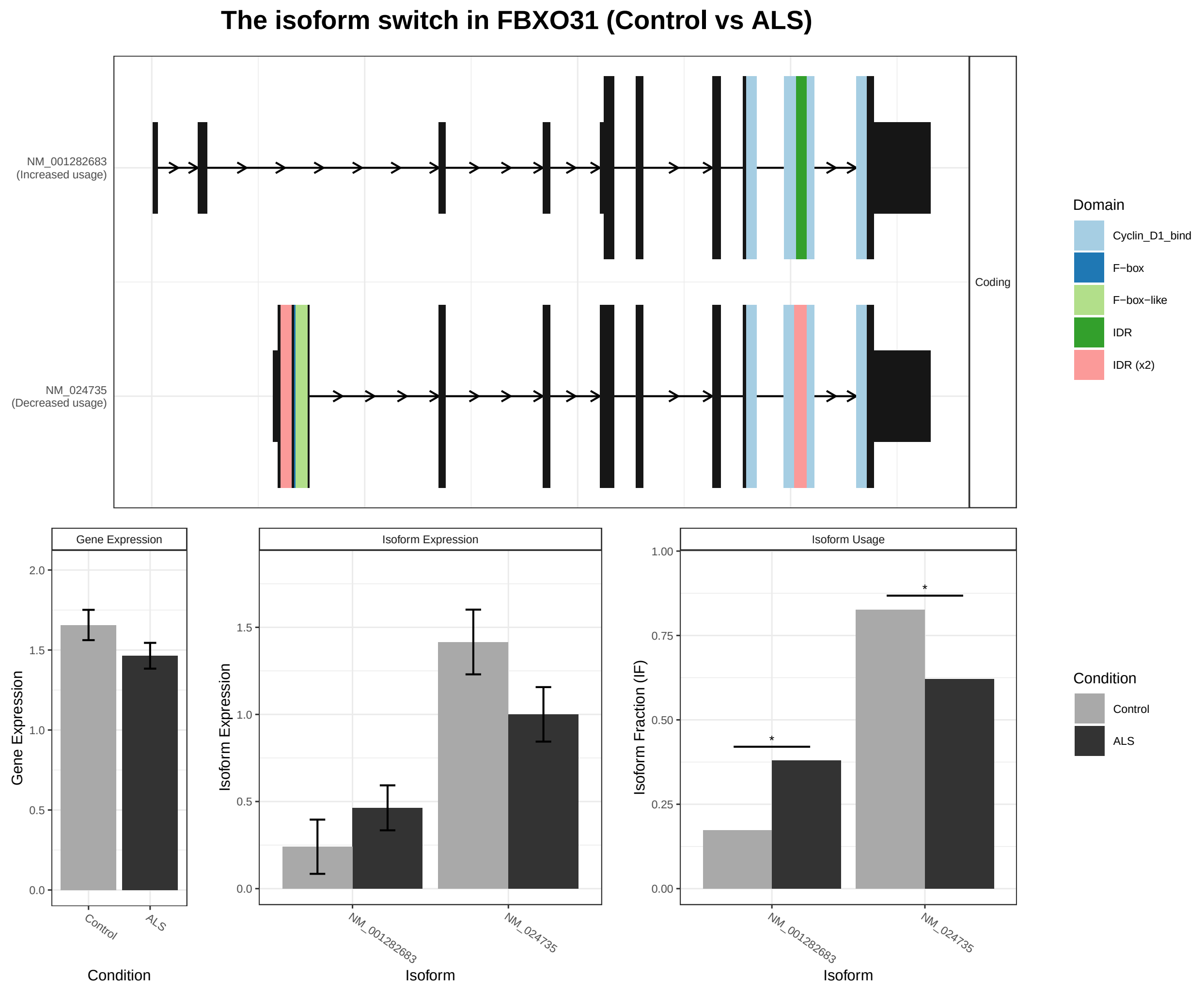

Fig. S7: Visual of 15 genes identified to have differential transcript usage between sALS patients and controls. *Top panel*: Visual depiction of assessed transcripts and their functional domains. Transcript IDs commencing with MSTRG are novel transcripts from StringTie *de novo* transcriptome assembly. *Bottom panel*: Comparison of gene expression (*left*), isoform expression (*middle*) and isoform fraction (*right*) between control and sALS groups. Significant differences are indicated by asterisk.

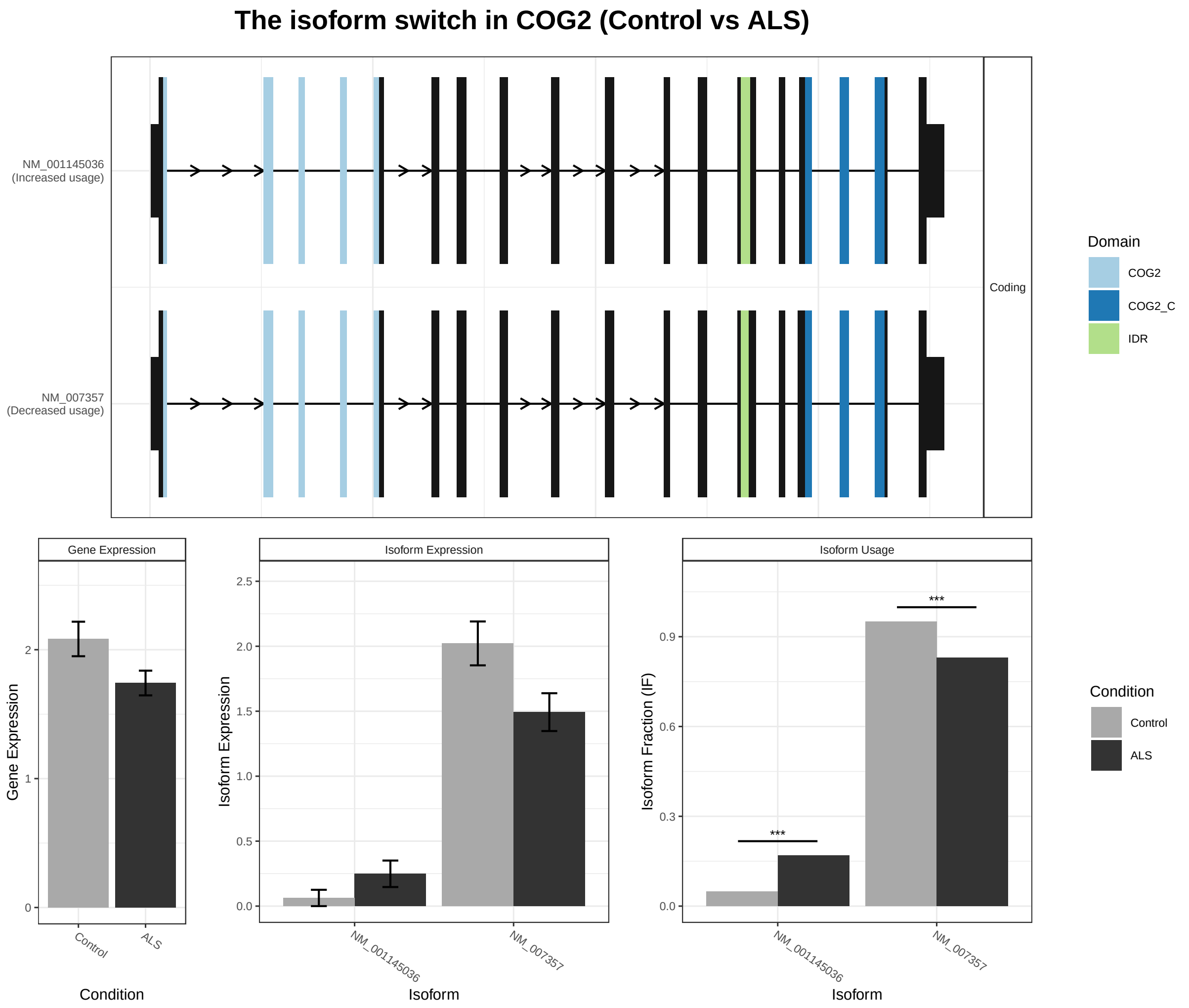
The isoform switch in COG2 (Control vs ALS)
NM_001145036
(Increased usage)
NM_007357
(Decreased usage)
Coding
Domain
COG2
COG2_C
IDR
Gene Expression
Isoform Expression
Isoform Usage
Gene Expression
Isoform Expression
Isoform Fraction (IF)
Control
ALS
NM_001145036
NM_007357
Condition
Isoform
Isoform
***
***
Condition
Control
ALS

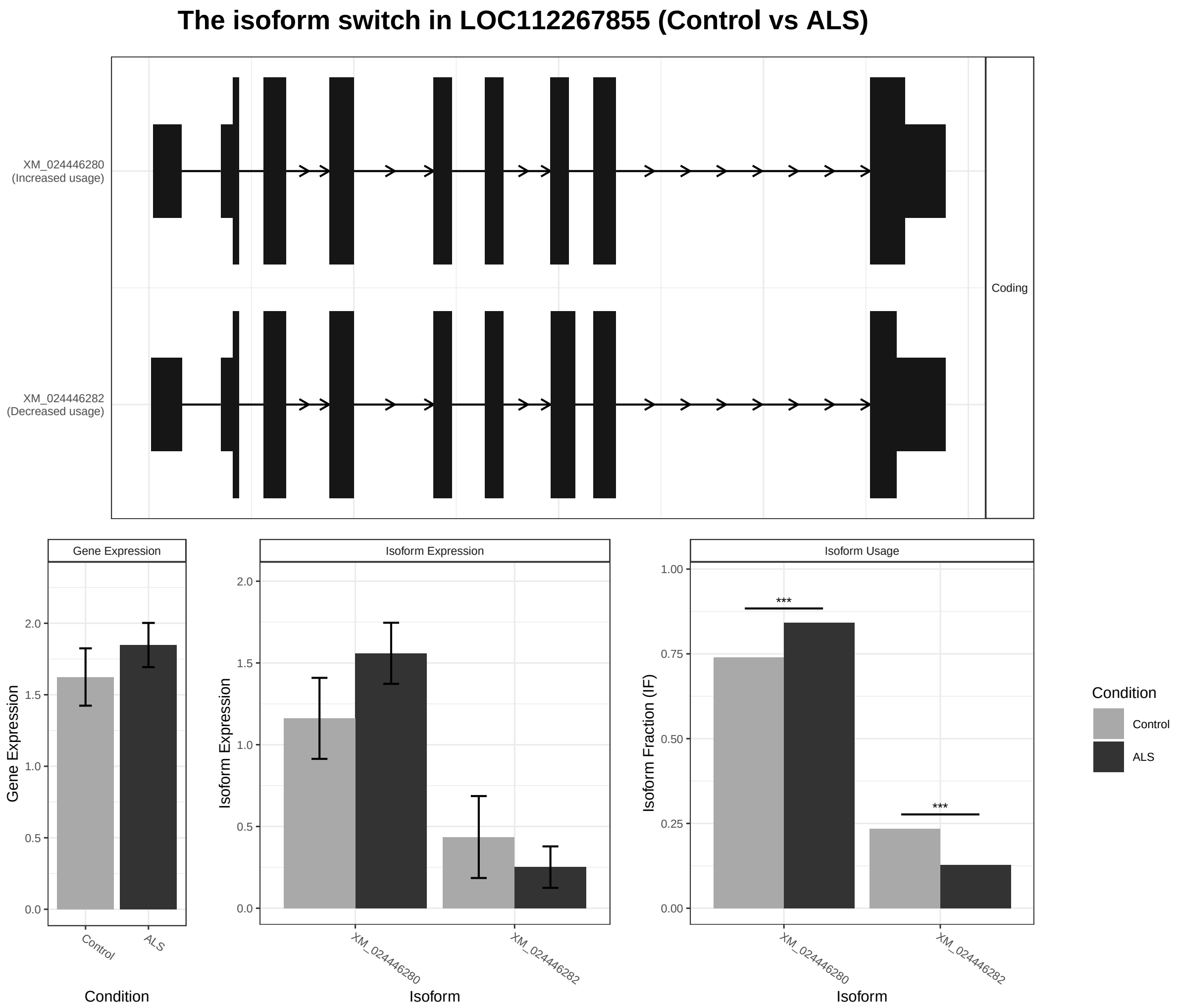
The isoform switch in LOC112267855 (Control vs ALS)
XM_024446280
(Increased usage)
XM_024446282
(Decreased usage)
Coding
Gene Expression
Isoform Expression
Isoform Usage
Gene Expression
Isoform Expression
Isoform Fraction (IF)
***
***
Control
ALS
XM_024446280
XM_024446282
Condition
Isoform
Isoform
Condition
Control
ALS

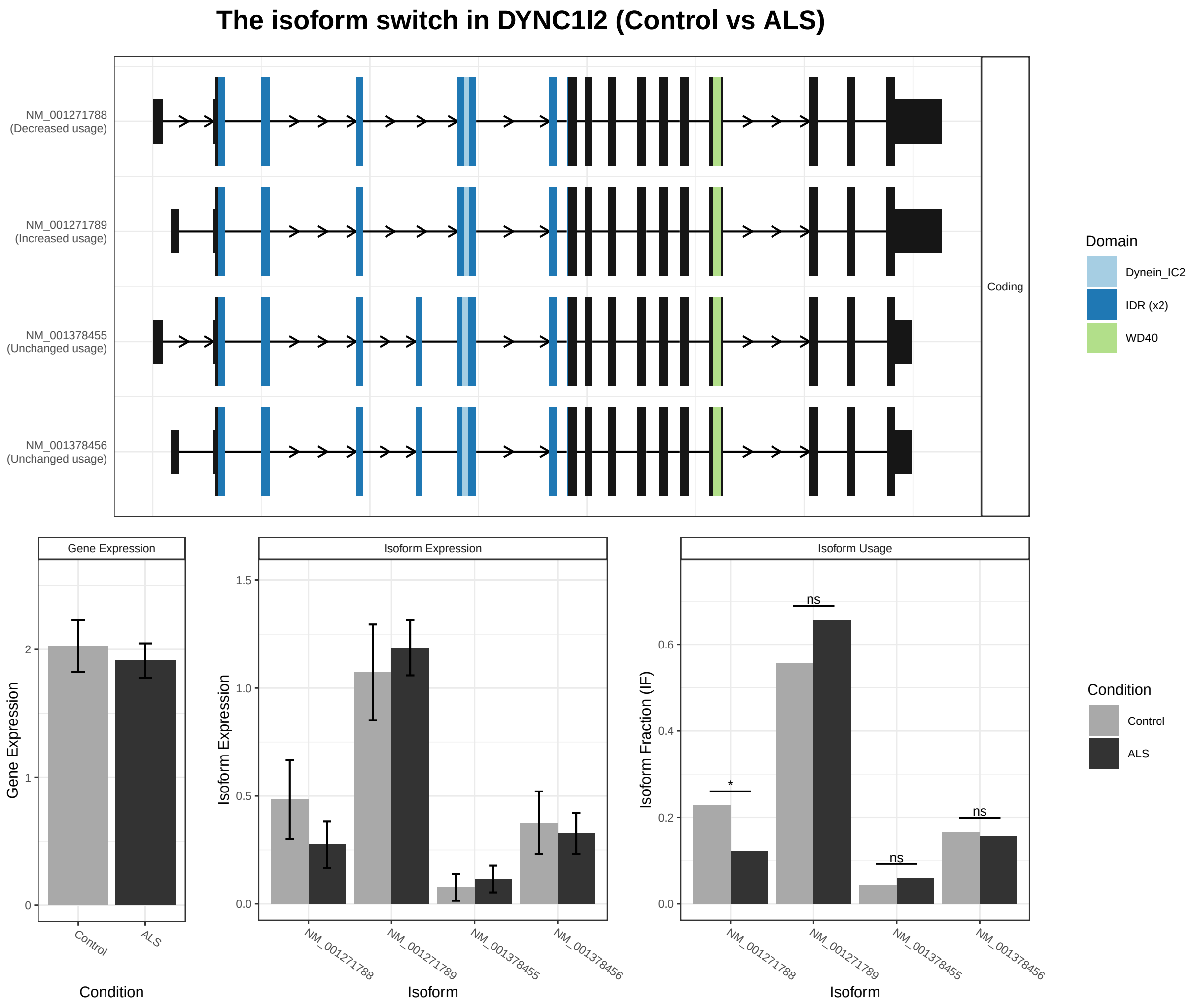
The isoform switch in DYNC1I2 (Control vs ALS)
NM_001271788
(Decreased usage)
NM_001271789
(Increased usage)
NM_001378455
(Unchanged usage)
NM_001378456
(Unchanged usage)
Coding
Domain
Dynein_IC2
IDR (x2)
WD40
Gene Expression
Isoform Expression
Isoform Usage
Gene Expression
Isoform Expression
Isoform Fraction (IF)
Condition
Control
ALS
Isoform
ns
*
NM_001271788
NM_001271789
NM_001378455
NM_001378456

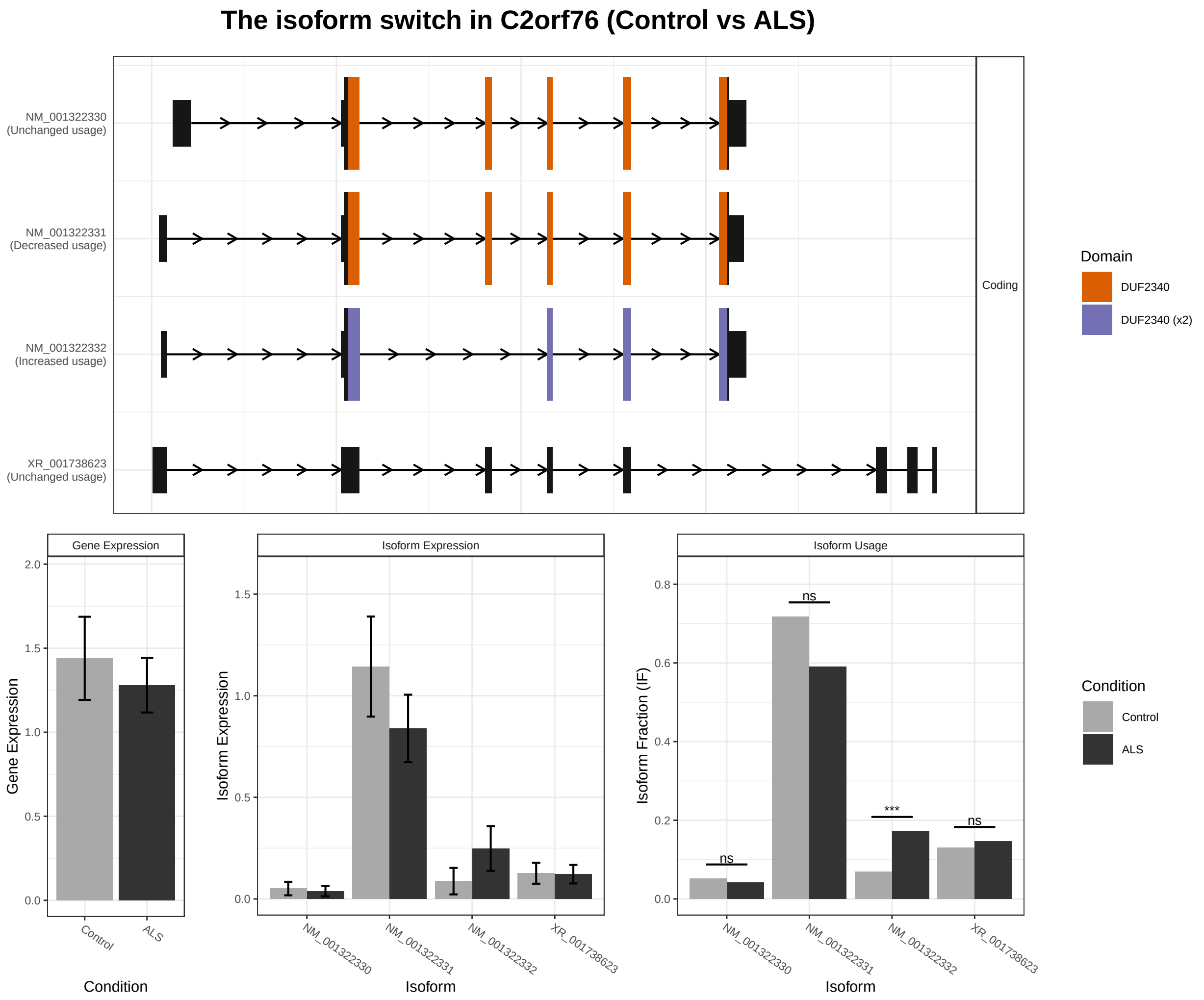

The isoform switch in C2orf76 (Control vs ALS)
NM_001322330 (Unchanged usage)
NM_001322331 (Decreased usage)
NM_001322332 (Increased usage)
XR_001738623 (Unchanged usage)
Coding
Domain
DUF2340
DUF2340 (x2)
Gene Expression
Isoform Expression
Isoform Usage
Gene Expression
Isoform Expression
Isoform Fraction (IF)
Control
ALS
Condition
Isoform
ns
***
Condition
Control
ALS

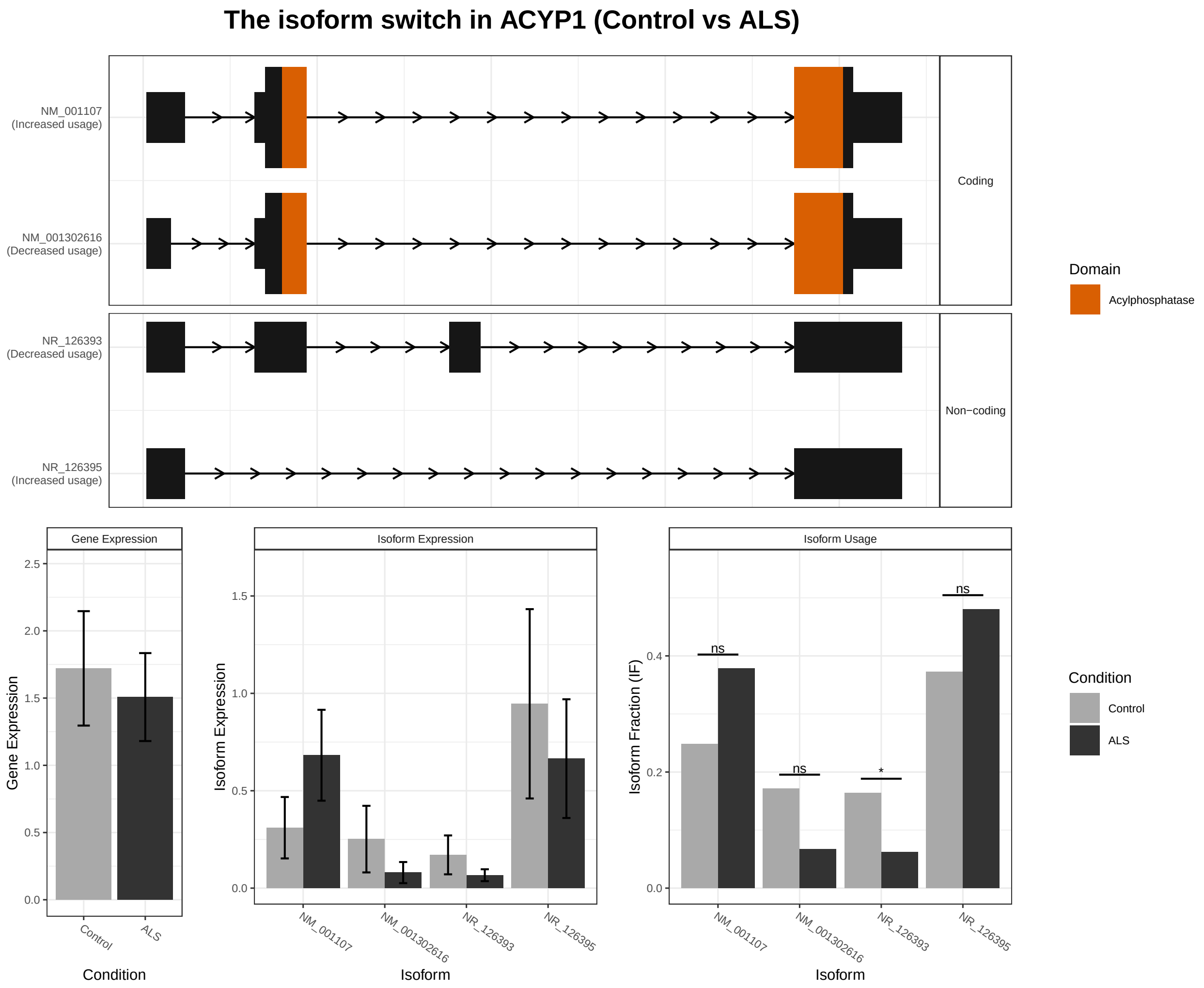

The isoform switch in ACYP1 (Control vs ALS)
NM_001107
(Increased usage)
NM_001302616
(Decreased usage)
NR_126393
(Decreased usage)
NR_126395
(Increased usage)
Coding
Non−coding
Domain
Acylphosphatase
Gene Expression
Isoform Expression
Isoform Usage
Gene Expression
Isoform Expression
Isoform Fraction (IF)
Control
ALS
Condition
Isoform
ns
*

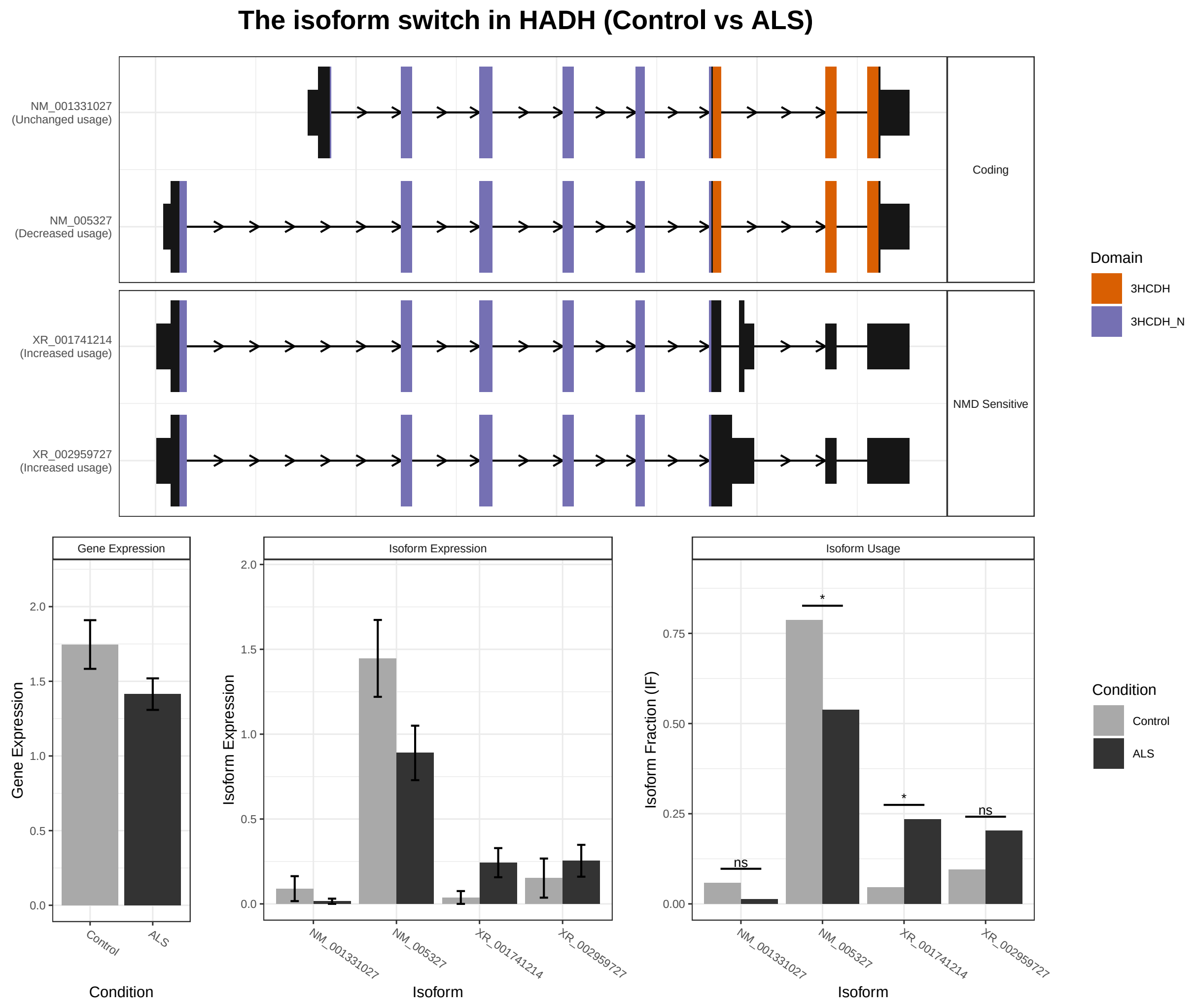
The isoform switch in HADH (Control vs ALS)
NM_001331027
(Unchanged usage)
NM_005327
(Decreased usage)
XR_001741214
(Increased usage)
XR_002959727
(Increased usage)
Coding
NMD Sensitive
Domain
3HCDH
3HCDH_N
Gene Expression
Isoform Expression
Isoform Usage
Gene Expression
Isoform Expression
Isoform Fraction (IF)
Condition
Isoform
Isoform
Control
ALS
NM_001331027
NM_005327
XR_001741214
XR_002959727
ns
*
*
ns
Condition
Control
ALS

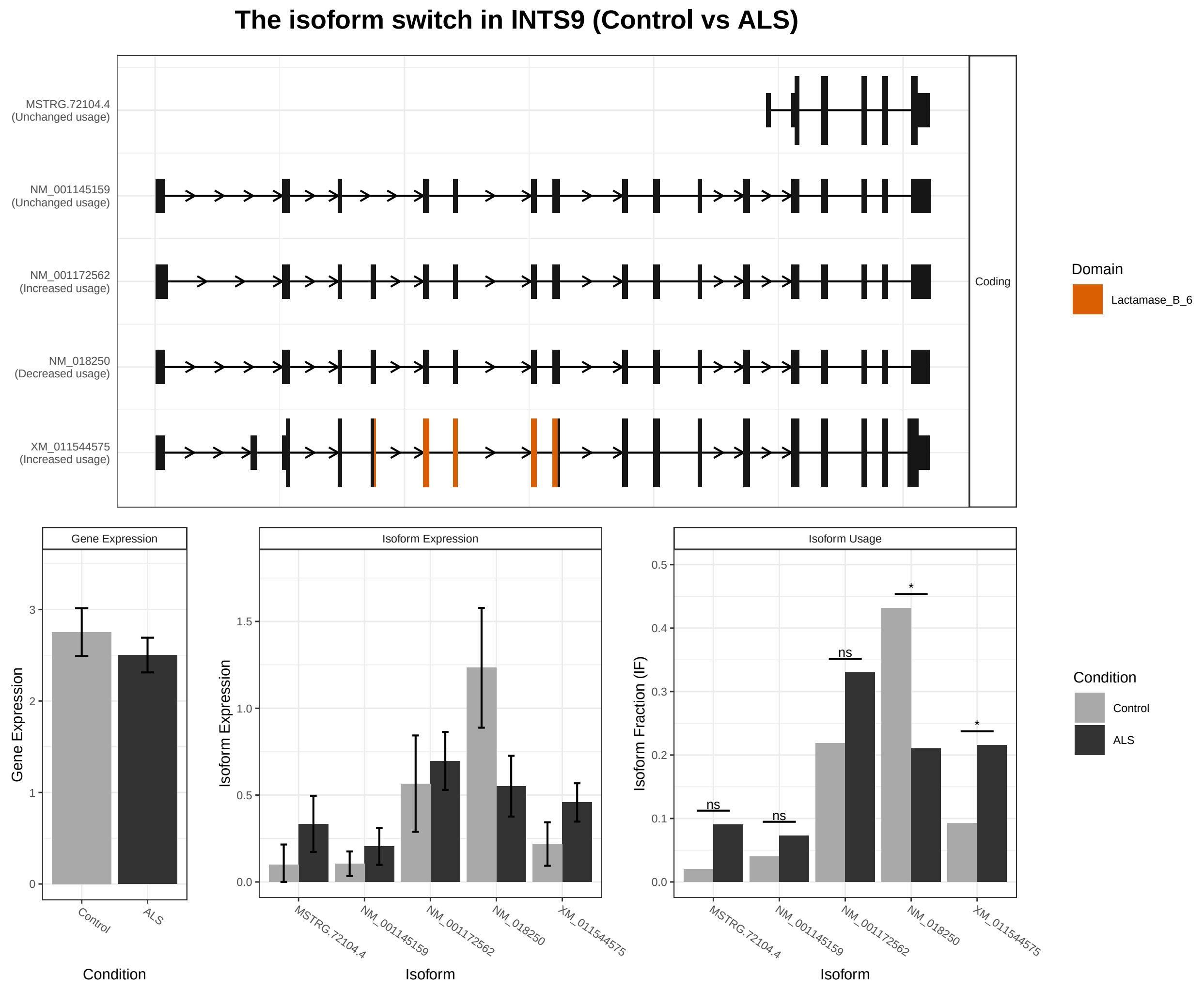

The isoform switch in INTS9 (Control vs ALS)
MSTRG.72104.4 (Unchanged usage)
NM_001145159 (Unchanged usage)
NM_001172562 (Increased usage)
NM_018250 (Decreased usage)
XM_011544575 (Increased usage)
Coding
Domain
Lactamase_B_6
Gene Expression
Isoform Expression
Isoform Usage
Condition
Control
ALS
Isoform
Isoform Fraction (IF)
ns
*

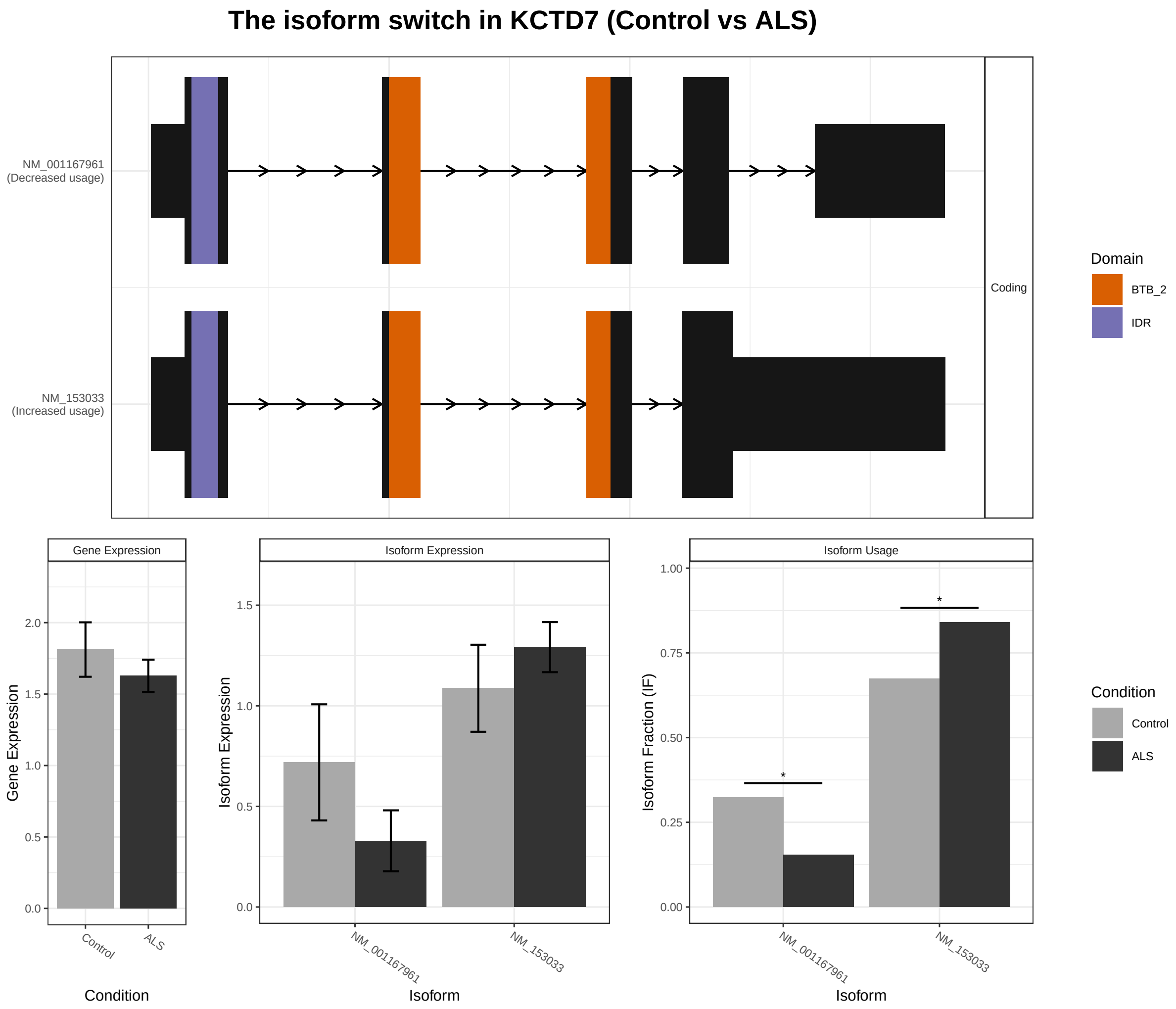
The isoform switch in KCTD7 (Control vs ALS)
NM_001167961 (Decreased usage)
NM_153033 (Increased usage)
Coding
Domain
BTB_2
IDR
Gene Expression
Isoform Expression
Isoform Usage
Gene Expression
Isoform Expression
Isoform Fraction (IF)
Control
ALS
Condition
NM_001167961
NM_153033
Isoform
Isoform
*
*
Condition
Control
ALS

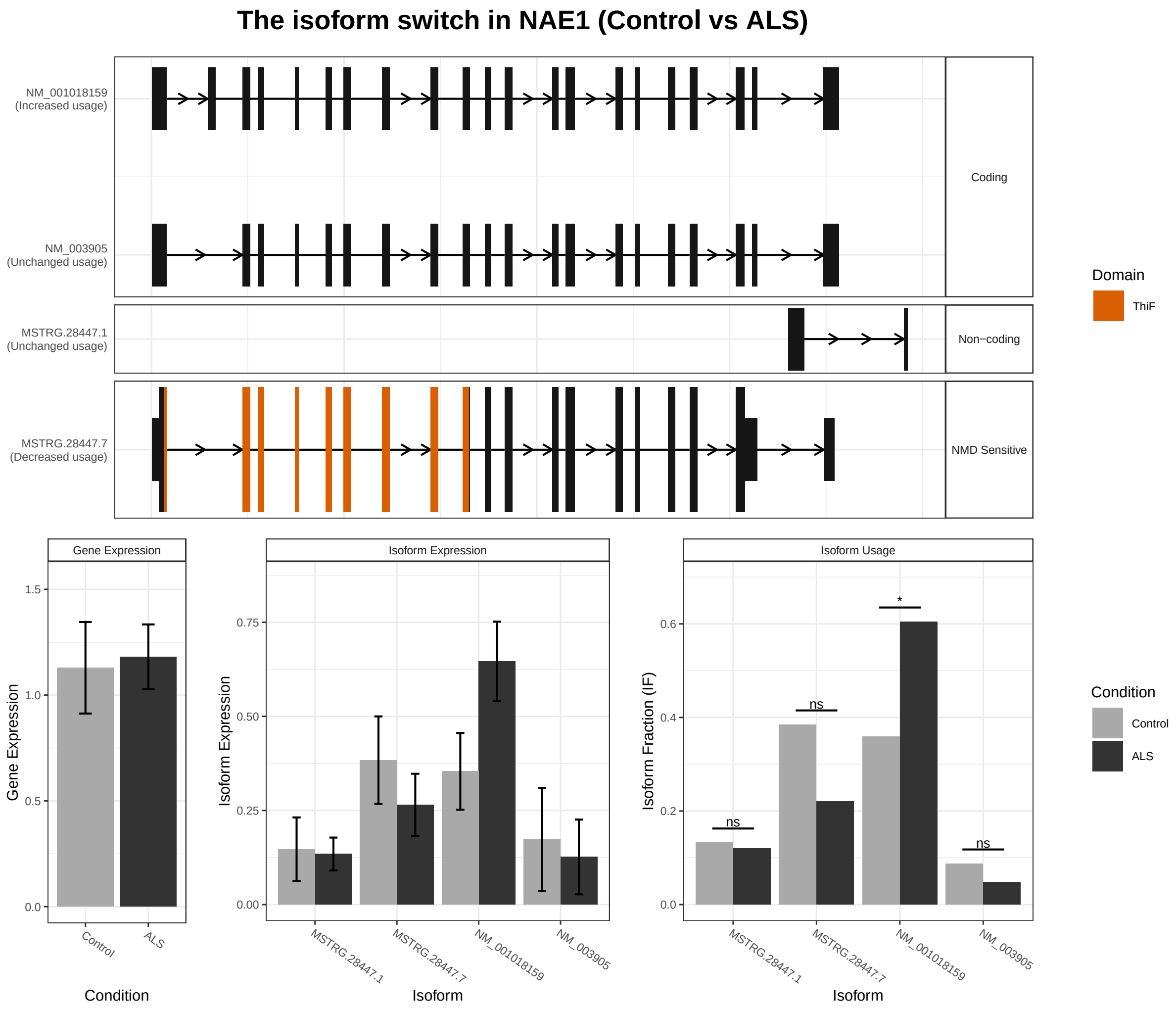

The isoform switch in NAE1 (Control vs ALS)
NM_001018159
(Increased usage)
NM_003905
(Unchanged usage)
Coding
MSTRG.28447.1
(Unchanged usage)
Non−coding
MSTRG.28447.7
(Decreased usage)
NMD Sensitive
Domain
ThiF
Gene Expression
Isoform Expression
Isoform Usage
Gene Expression
Isoform Expression
Isoform Fraction (IF)
Control
ALS
MSTRG.28447.1
MSTRG.28447.7
NM_001018159
NM_003905
ns
*
Condition
Isoform

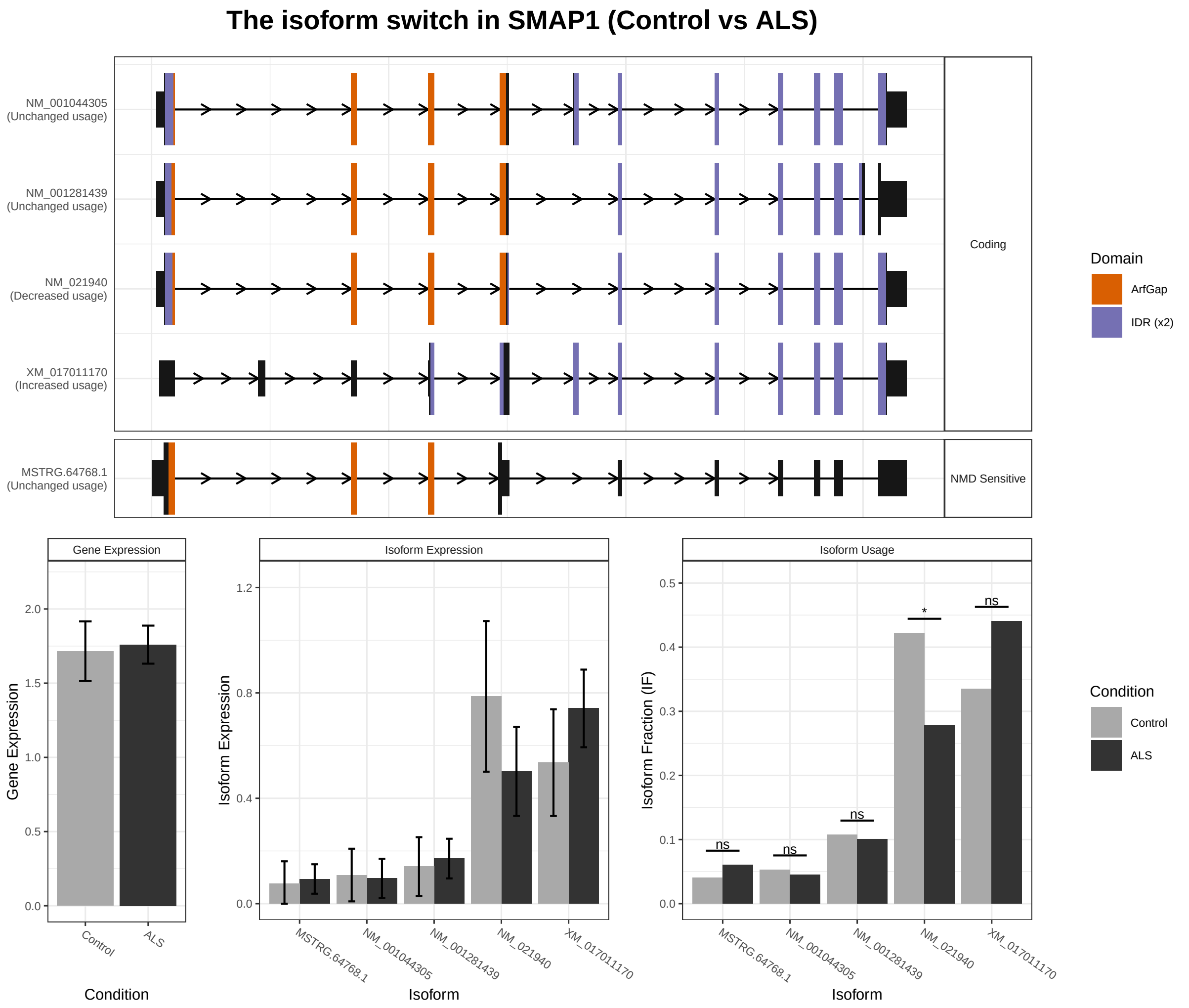
The isoform switch in SMAP1 (Control vs ALS)
NM_001044305 (Unchanged usage)
NM_001281439 (Unchanged usage)
NM_021940 (Decreased usage)
XM_017011170 (Increased usage)
MSTRG.64768.1 (Unchanged usage)
Coding
NMD Sensitive
Domain
ArfGap
IDR (x2)
Gene Expression
Isoform Expression
Isoform Usage
Gene Expression
Isoform Expression
Isoform Fraction (IF)
Condition
Isoform
Control
ALS
ns
*
MSTRG.64768.1
NM_001044305
NM_001281439
NM_021940
XM_017011170

The isoform switch in DVL1 (Control vs ALS)

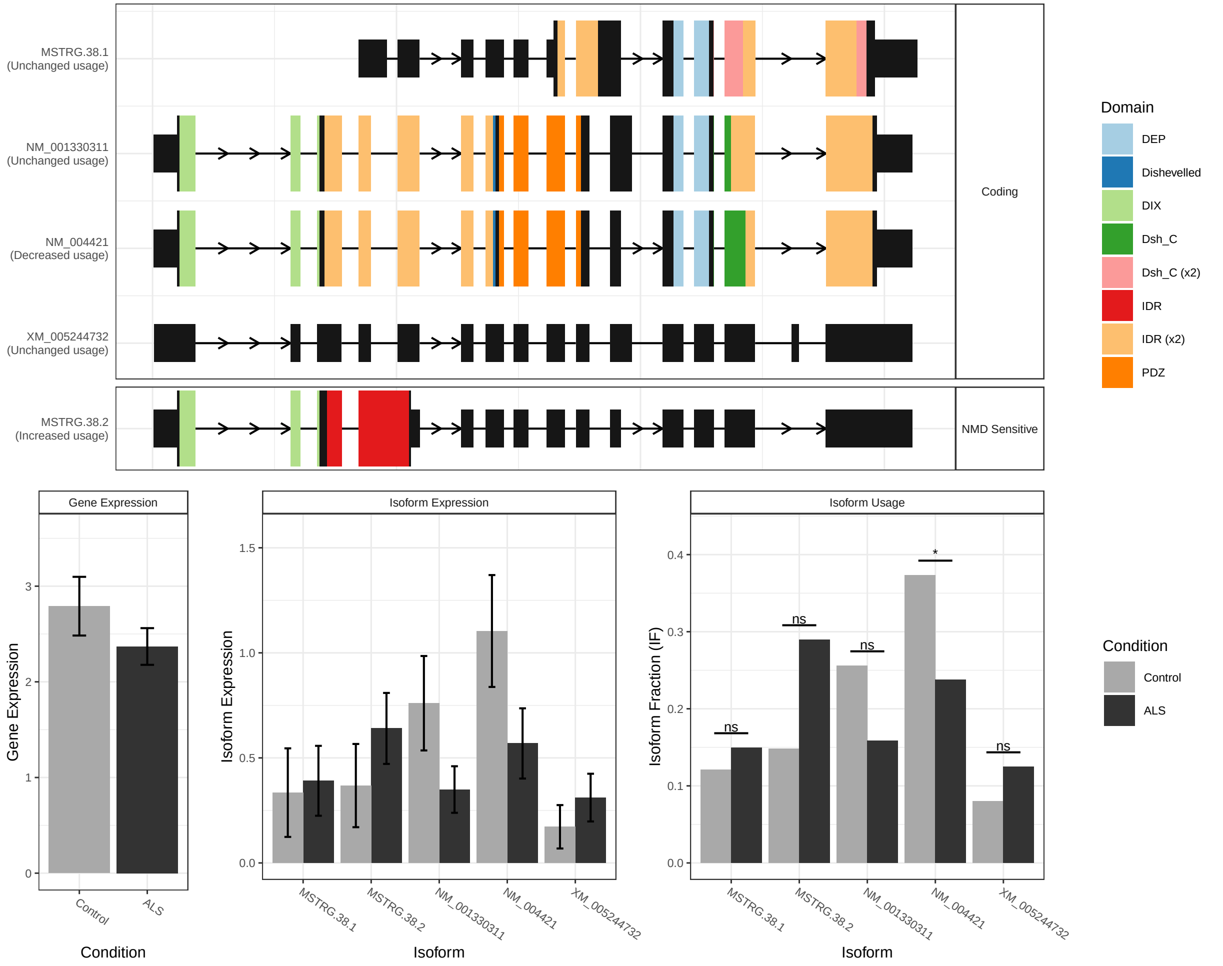

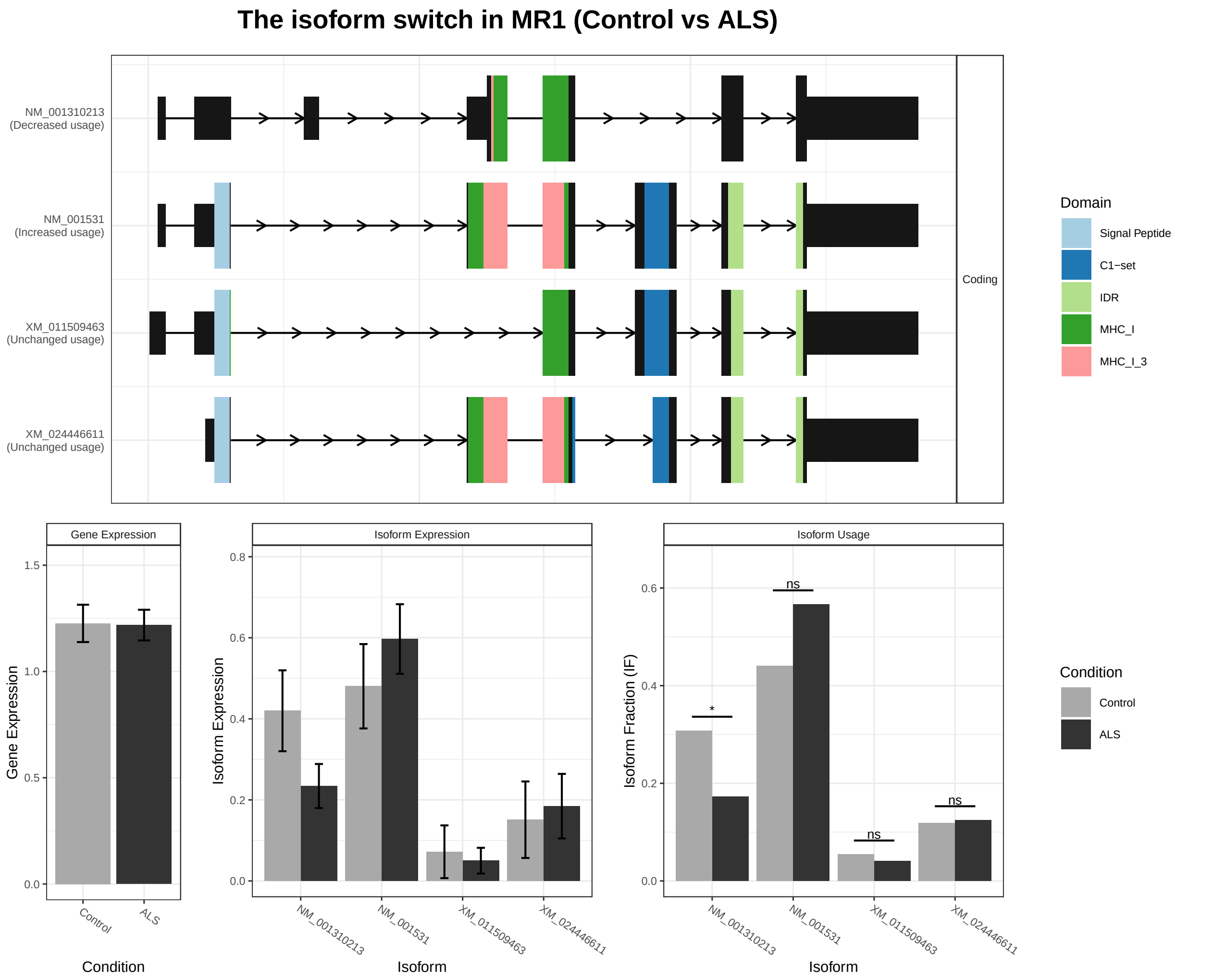
The isoform switch in MR1 (Control vs ALS)
NM_001310213 (Decreased usage)
NM_001531 (Increased usage)
XM_011509463 (Unchanged usage)
XM_024446611 (Unchanged usage)
Coding
Domain
Signal Peptide
C1−set
IDR
MHC_I
MHC_I_3
Gene Expression
Isoform Expression
Isoform Usage
Gene Expression
Isoform Expression
Isoform Fraction (IF)
0.0
0.5
1.0
1.5
0.2
0.4
0.6
0.8
Control
ALS
Condition
NM_001310213
NM_001531
XM_011509463
XM_024446611
Isoform
*
ns
Condition
Control
ALS

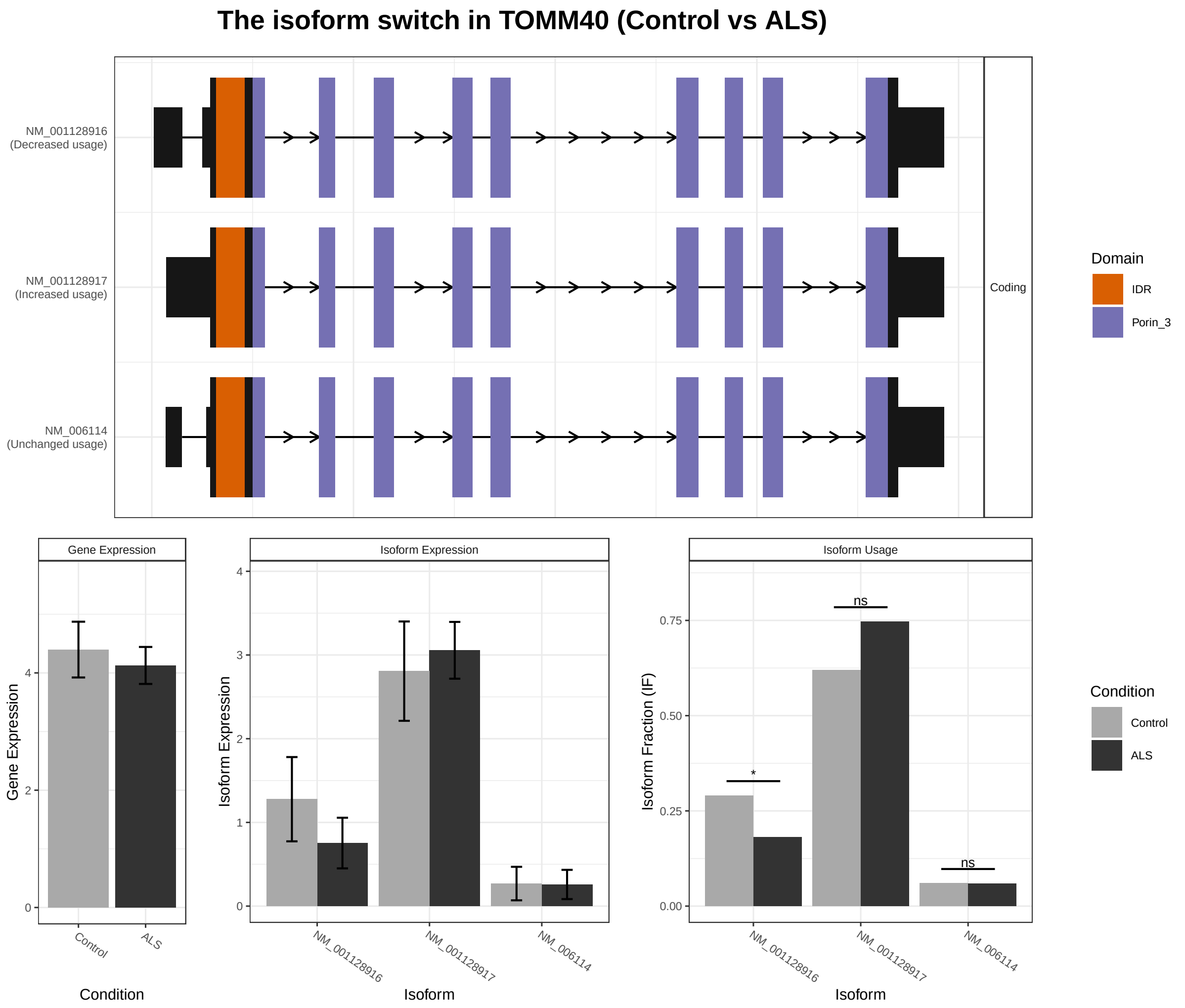

The isoform switch in TOMM40 (Control vs ALS)
NM_001128916
(Decreased usage)
NM_001128917
(Increased usage)
NM_006114
(Unchanged usage)
Coding
Domain
IDR
Porin_3
Gene Expression
Isoform Expression
Isoform Usage
Gene Expression
Control
ALS
Condition
Isoform Expression
NM_001128916
NM_001128917
NM_006114
Isoform
Isoform Fraction (IF)
ns
*
Condition
Control
ALS

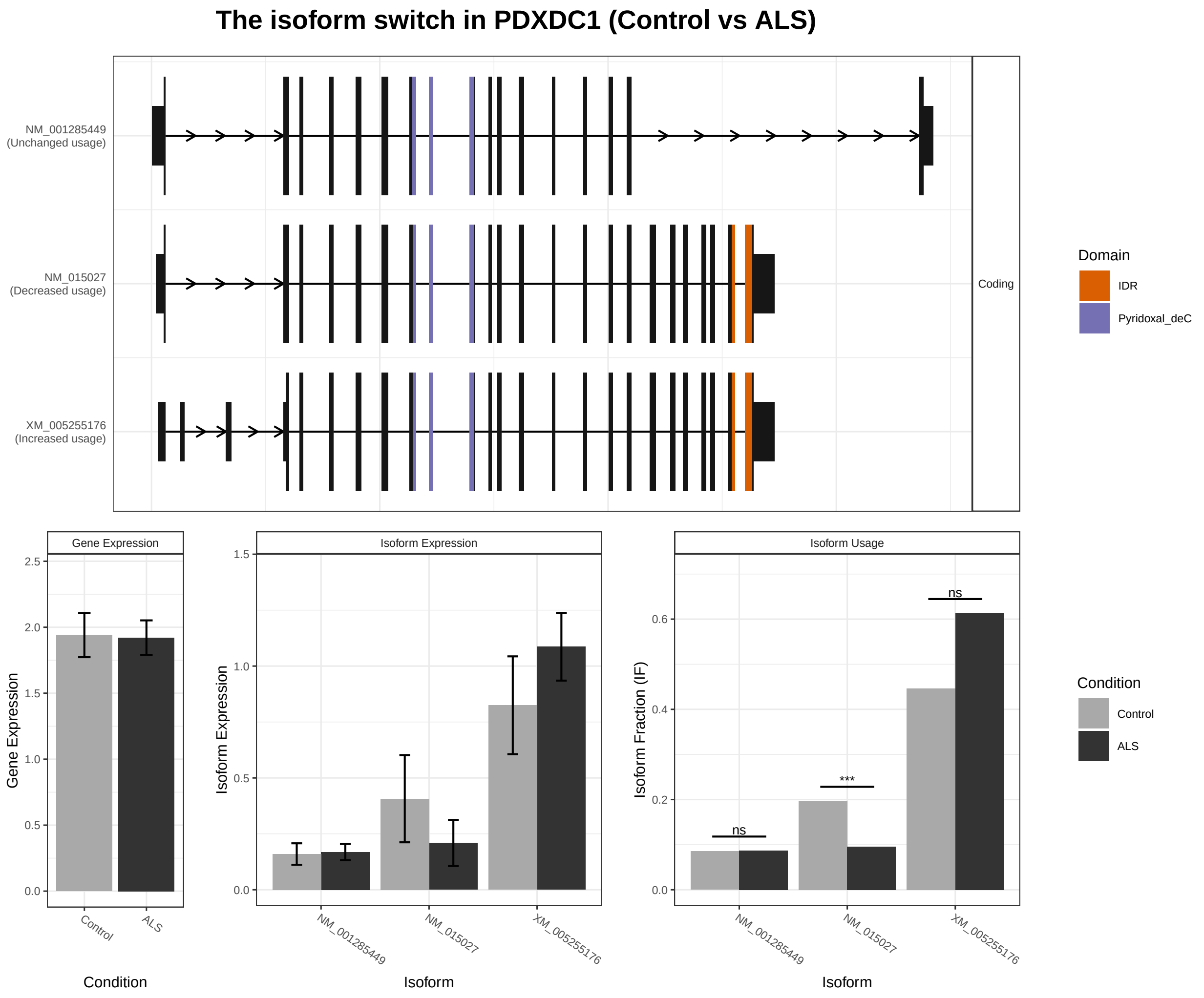

The isoform switch in PDXDC1 (Control vs ALS)
NM_001285449 (Unchanged usage)
NM_015027 (Decreased usage)
XM_005255176 (Increased usage)
Coding
Domain
IDR
Pyridoxal_deC
Gene Expression
Isoform Expression
Isoform Usage
Gene Expression
Isoform Expression
Isoform Fraction (IF)
Condition
Isoform
Isoform
Control
ALS
NM_001285449
NM_015027
XM_005255176
ns
***
ns
Condition
Control
ALS

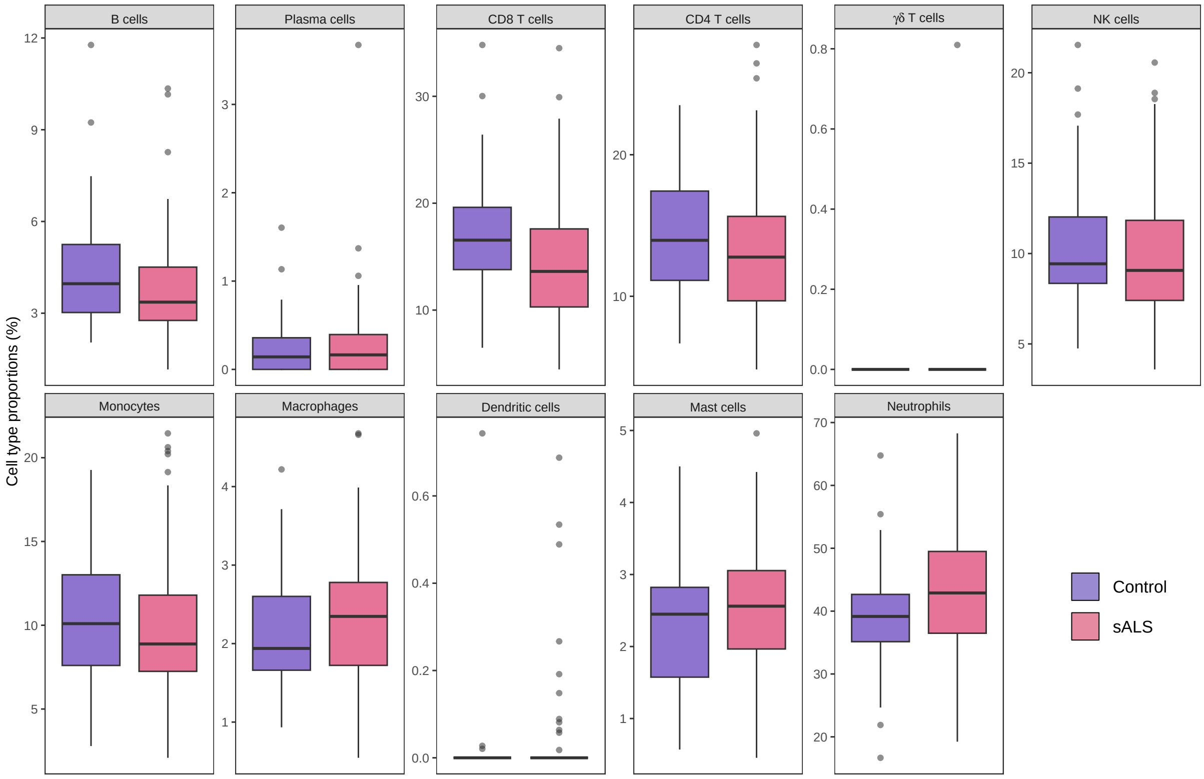

Fig. S8: Proportions of 12 major leukocytes in sALS versus control peripheral blood. No cell type proportions were significantly different between sALS and control groups (Welch Two Sample t-test with Bonferroni correction, FDR<0.05). Cell types were deconvolved from bulk blood RNA-seq using CIBERSORTx and the LM22 signature matrix. Eosinophils were not detected and are therefore excluded.

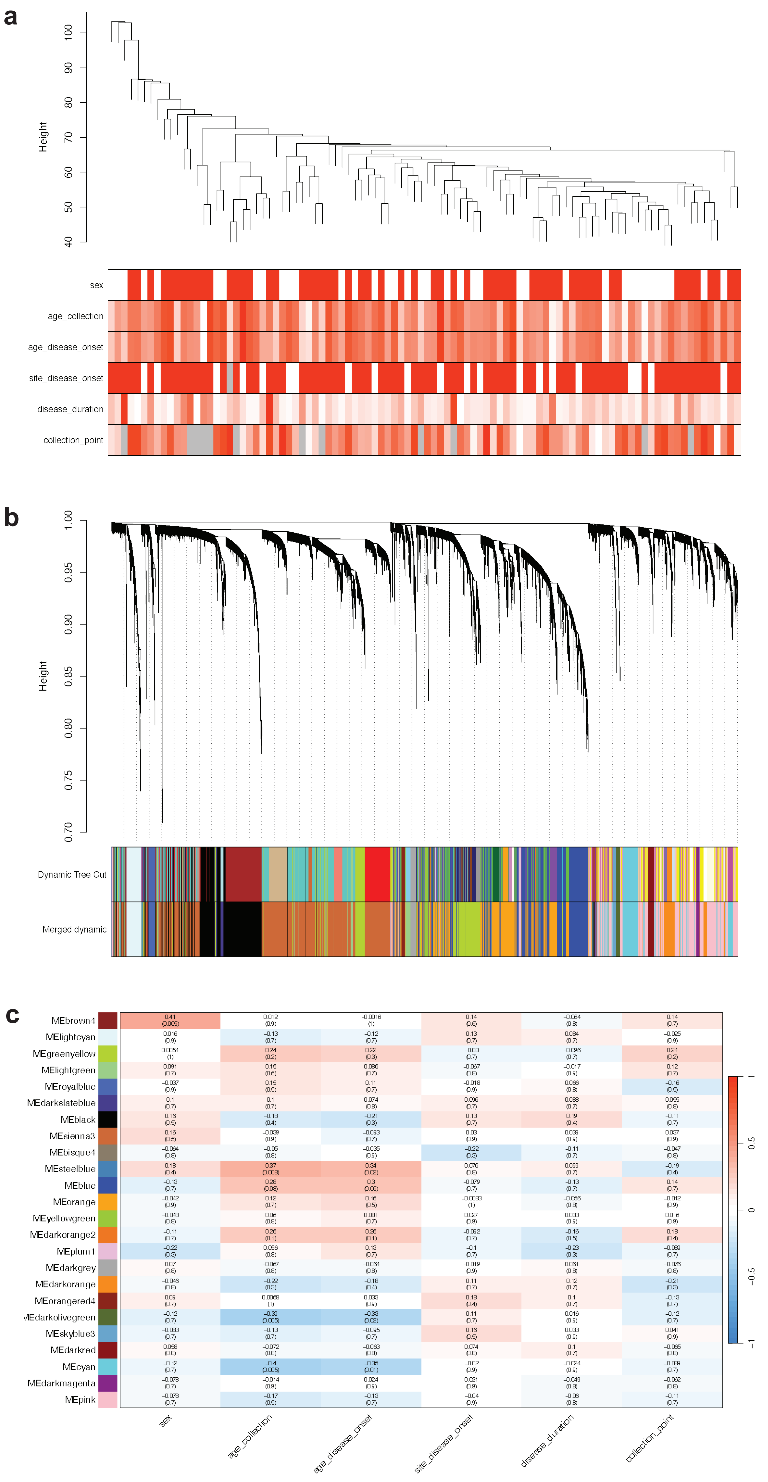

Fig. S9: sALS patient clustering, module identification and association with clinical traits for Weighted Gene Co-expression Network Analysis (WGCNA). a Sample dendrogram with clinical traits heatmap. Red is male in sex, spinal in site_disease_onset and the higher value in age_collection, age_disease_onset, disease_duration and collection_point. Grey indicates NA. **b** Clustering dendrogram of genes, where dissimilarity is based on topological overlap, with assigned module colours. Initial network construction yielded 48 putative modules. Merging modules with highly correlated eigengenes resulted in 24 modules. **c** Complete module-trait association heatmap where rows correspond to module eigengenes and columns correspond to a clinical trait. Each cell contains the Spearman correlation coefficient (also indicated by colour legend) and Benjamini-Hochberg adjusted p-value in brackets (FDR<0.05).

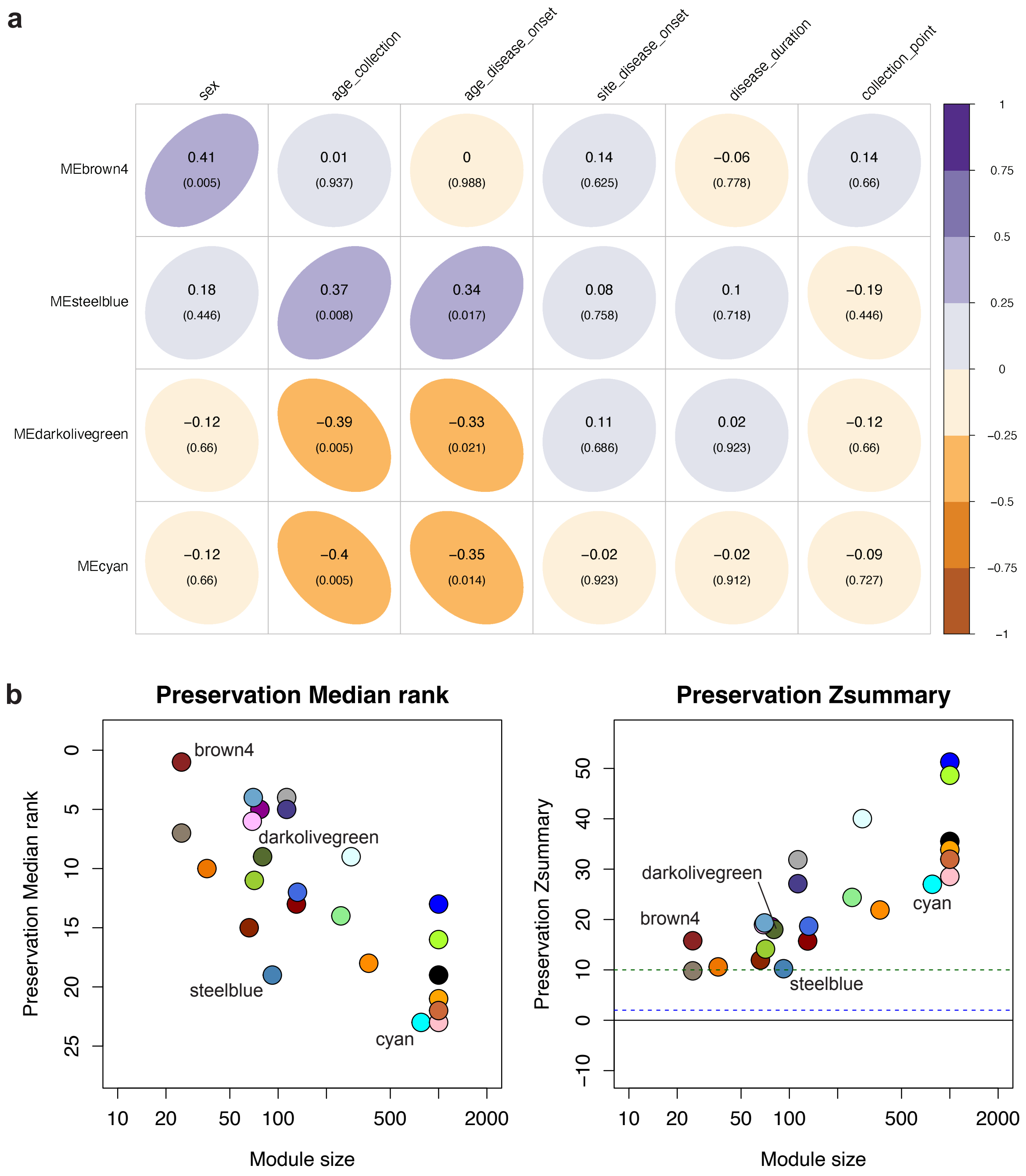

Fig. S10: Weighted Gene Co-expression Network Analysis (WGCNA) of sALS (n=96) identified four modules associated with sex, age at collection or age at disease onset which were preserved in control individuals. **a** The four module eigengenes significantly correlated with clinical traits are shown. Spearman correlation coefficient is indicated by the upper value and colour gradient and Benjamini-Hochberg adjusted p-value is in brackets. Correlations with FDR<0.05 were considered to be significant. **b** Module preservation analysis identified that sALS co-expression modules were preserved in controls (n=48). Two composite preservation statistics are provided. Preservation Median rank, ranks modules based on their observed preservation statistics where 1 is best. Preservation $Z_{summary}$ scores module preservation where $Z_{summary} > 2$ (blue line) indicates low to moderate evidence of conservation and $Z_{summary} > 10$ (green line) indicates strong evidence for conservation.

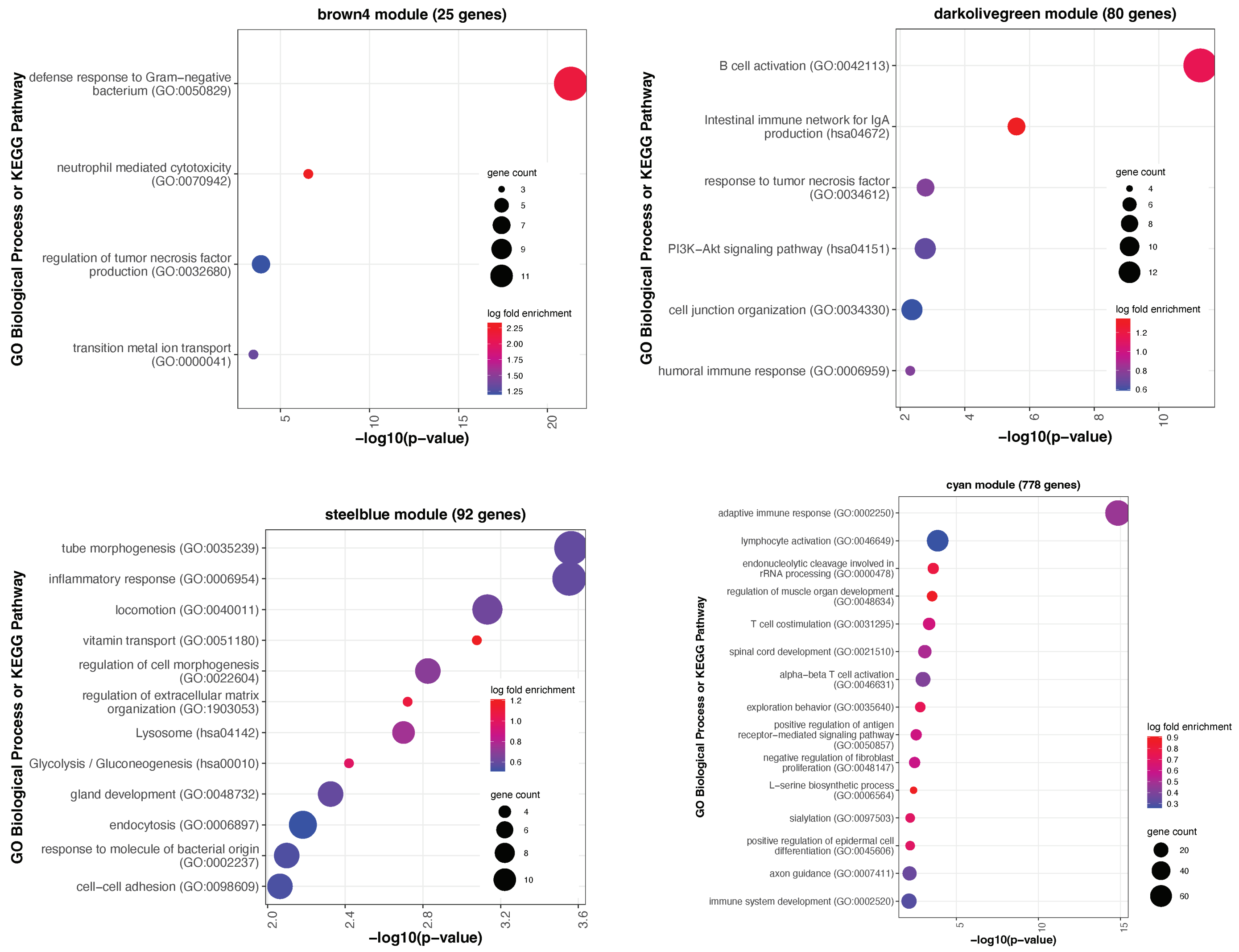

Fig. S11: GO biological processes and KEGG pathways enriched in the four co-expression modules associated with sex (brown4) or age at collection and age at disease onset (steelblue, darkolivegreen, cyan). Gene count indicates the number of module genes in each pathway. Log2 fold change indicates enrichment of member genes in module genes versus all detected genes.

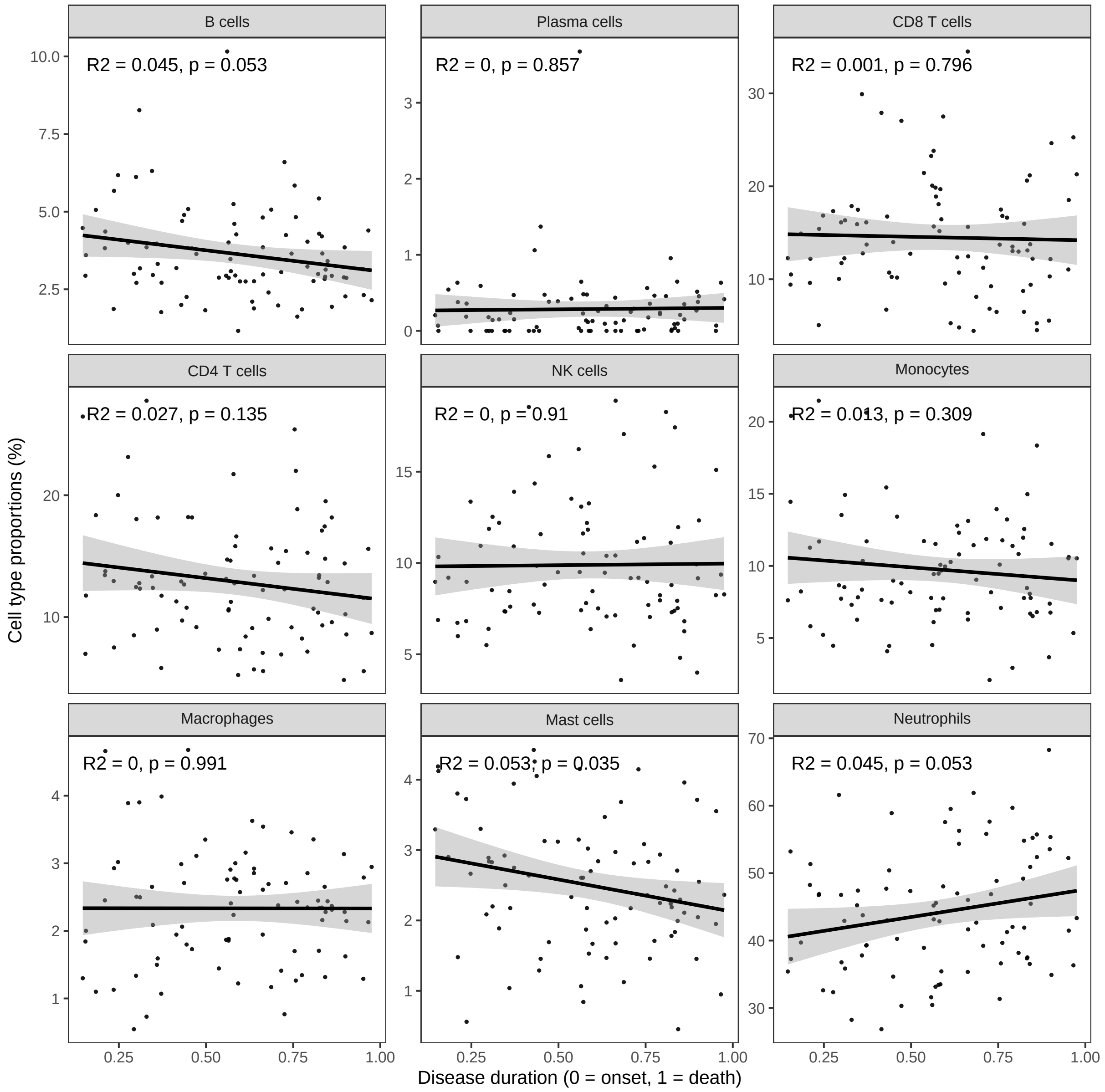

Fig. S12: The percentage of leukocytes in the blood of sALS patients against the stage in disease duration at which blood was collected. Cell type proportions were estimated from peripheral blood RNA-seq using CIBERSORTx and the LM22 signature matrix. Data is displayed with line of best-fit and 95% confidence intervals. Multiple R-squared (R2) and p-value (p) from linear regression are shown. Dendritic cells, eosinophils and $\gamma\delta$ T cells were excluded as they were not detected in $\geq$ 50% of cases.

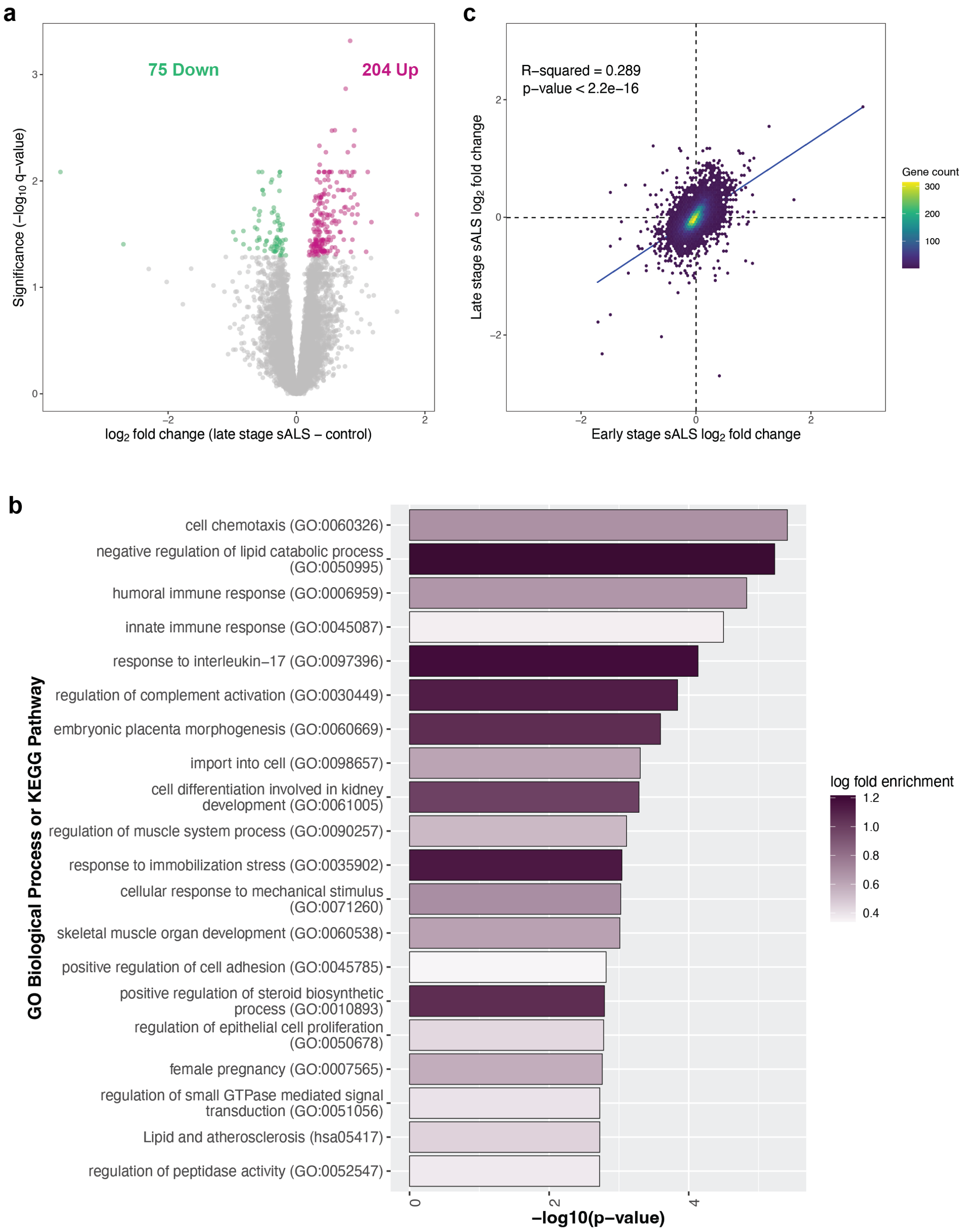

Fig. S13: Differentially expressed genes were identified for late but not early-stage sALS patients relative to controls. sALS were defined as early- or late-stage (n=28 each) based on when in disease duration sample collection occurred. **a** Volcano plot comparing late-stage sALS with controls. Pink and green dots represent genes that are upregulated and downregulated, respectively (FDR <0.05) while grey dots are genes that are not differentially expressed. **b** GO biological processes and KEGG pathways enriched in the late-stage sALS-control differentially expressed genes. The most representative member (lowest p-value) of each GO term cluster is displayed. Log2 fold change indicates enrichment of member genes in differentially expressed genes versus all detected genes. **c** Scatter plots comparing $\log_2$ fold changes of all genes tested between early- and late-stage sALS relative to controls.

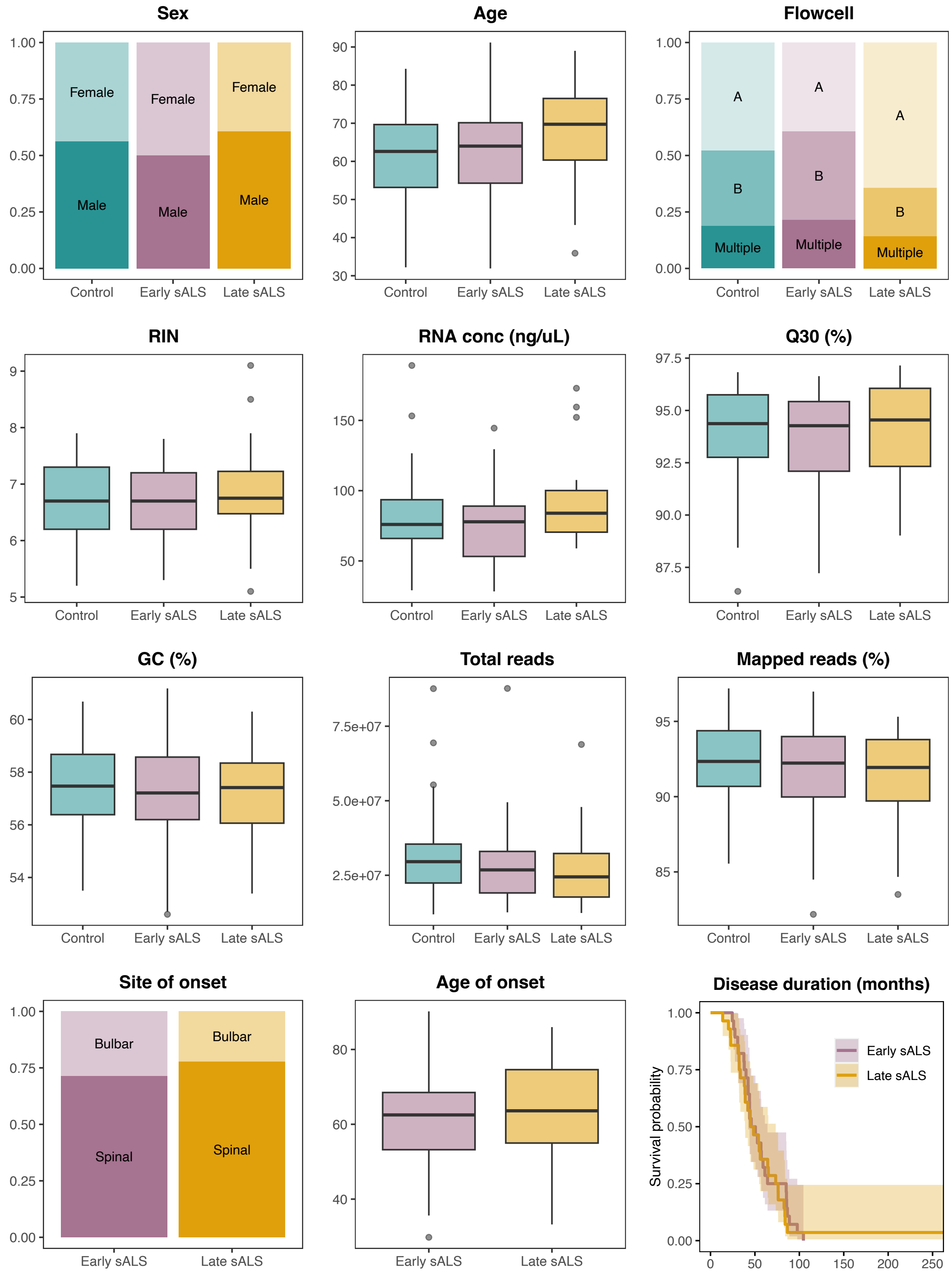

Fig. S14: Comparison of sample features between controls (n=48) and early-stage (n=28) or late-stage (n=28) sALS patients. No features were significantly different between groups. Results of statistical comparisons can be found in Table S10. Sequencing metrics are all post-trimming. 95% confidence intervals are shown for Kaplan-Meier curve. RIN, RNA integrity number; Q30%, ratio of bases that have phred quality score ≥ 30.

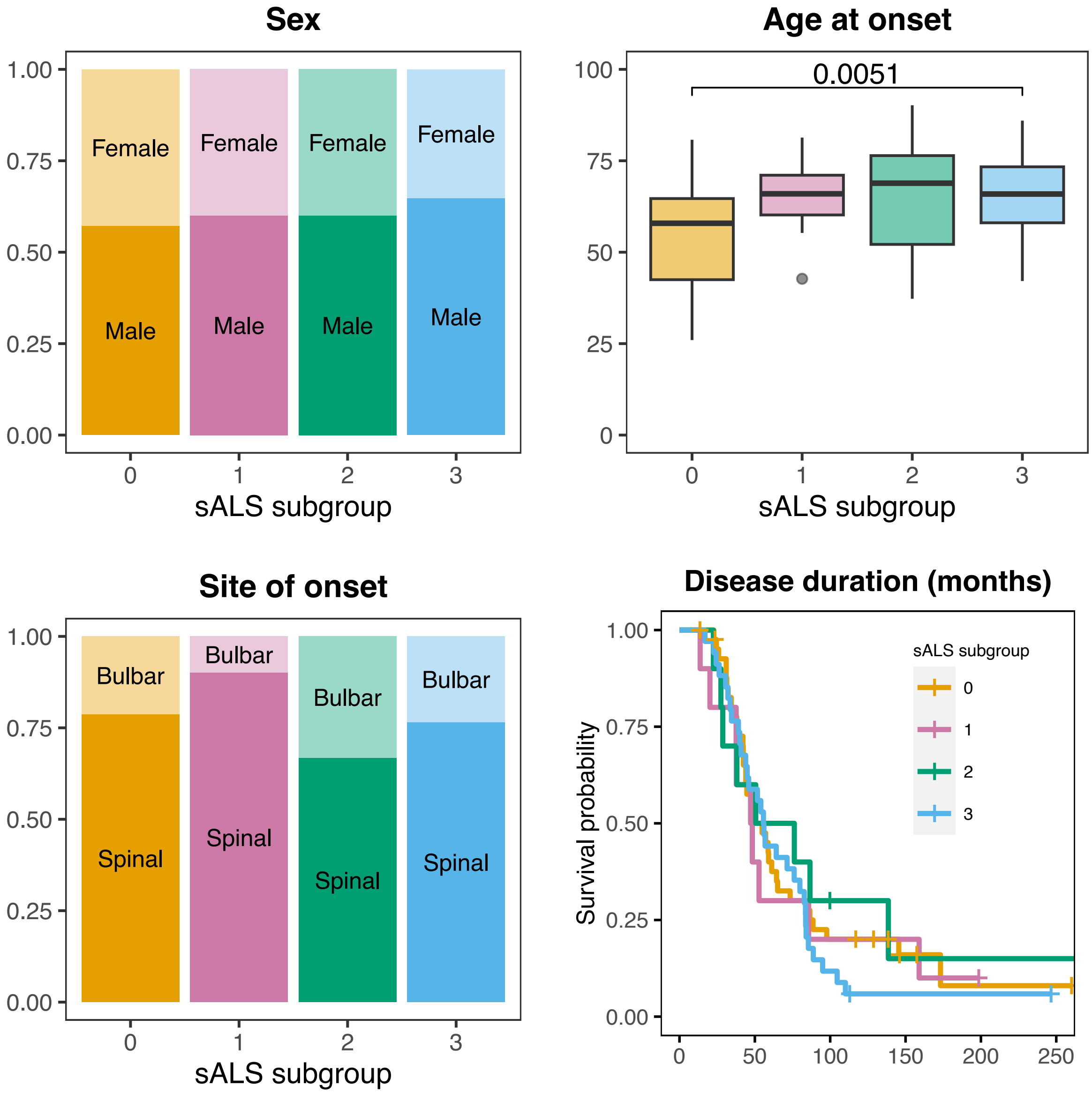

Fig. S15: Comparison of clinical features between sALS patient subgroups. Age at collection and age at onset were significantly different between select sALS patient subgroups. Results of statistical comparisons, including technical sample features, can be found in Table S11.

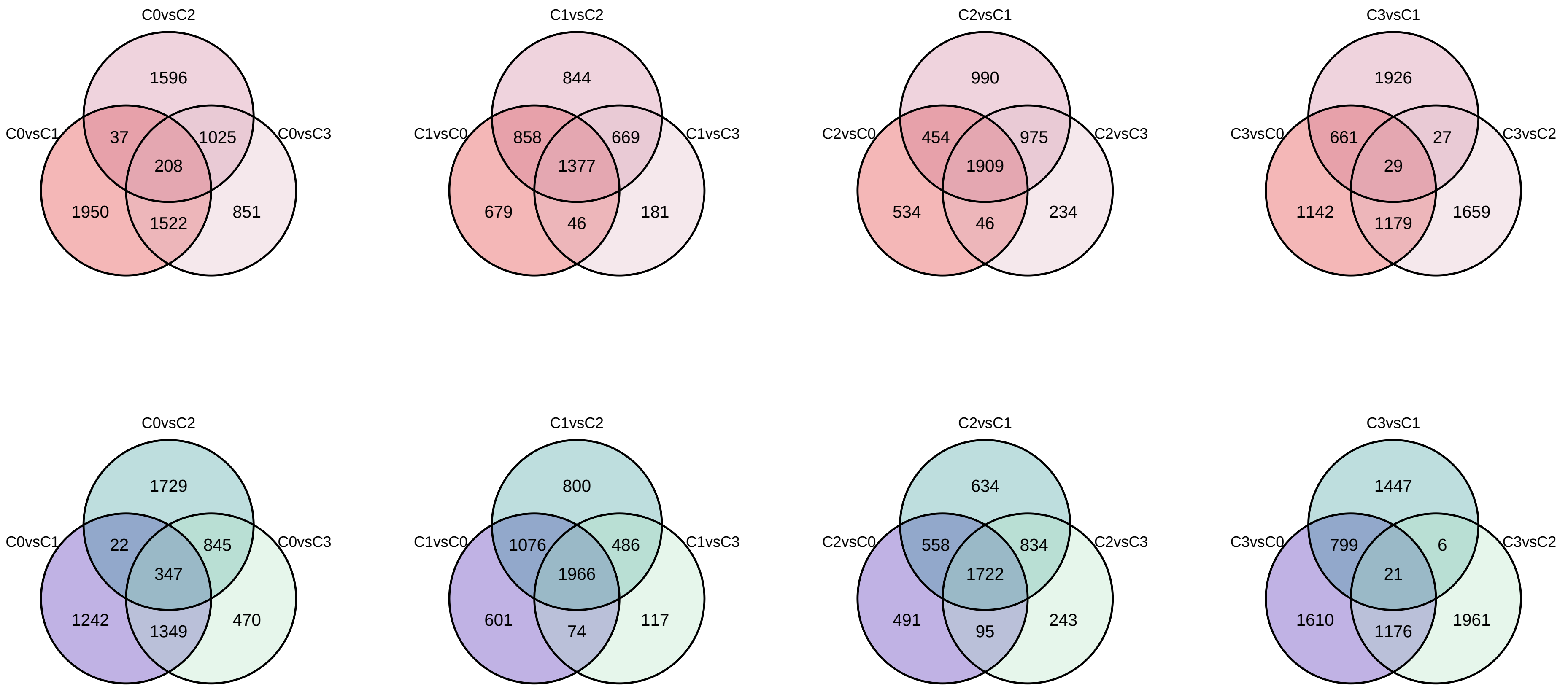

Fig. S16: Venn diagrams highlighting the overlap in differentially expressed genes between each sALS subgroup comparison. Direct comparisons were made between each subgroup identified by unsupervised clustering analysis. The overlap of genes identified as significantly up- or down-regulated from all comparisons (FDR<0.05) were classified as subgroup defining genes (centre figure from each Venn diagram). Upregulated and downregulated genes are presented in the top (red) and bottom (blue) rows, respectively. C0, cluster 0; C1, cluster 1; C2, cluster 2; C3, cluster 3.

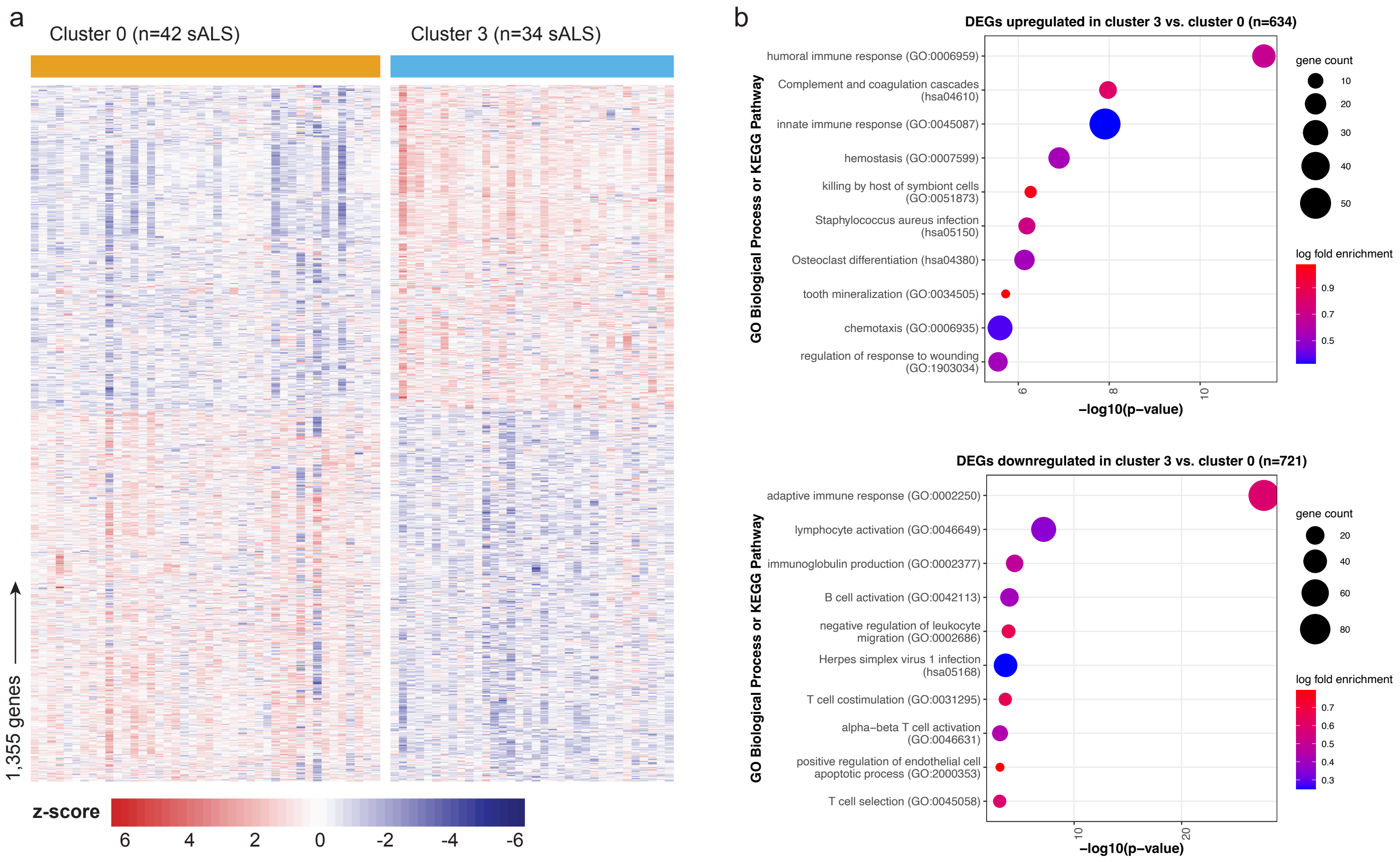

Fig. S17: Differentially expressed genes identified between sALS cluster 0 and cluster 3 are enriched for immune related pathways. a Heatmap of 1,355 genes identified to be differentially expressed between cluster 0 and cluster 3 (FDR<0.05, FC>1.5 or FC<0.67). Gene counts are z-score normalised. **b** GO biological processes and KEGG pathways enriched in genes significantly upregulated or downregulated in cluster 3 relative to cluster 0. The most representative member (lowest p-value) of the top ten GO term clusters is displayed. Gene count indicates the number of differentially expressed genes in each pathway. Log2 fold change indicates enrichment of member genes in differentially expressed genes versus all detected genes. Complete list of enriched pathways can be found in Tables S13g-h.

Supplementary Tables

Table S2: Statistical results from comparison of sample features between sALS (n=96) and control (n=48) groups.

Sample variable	p-value	Statistical test
Sex	0.7645197	Pearson's Chi-squared test with Yates' continuity correction
Age	0.1900126	Welch Two Sample t-test
Flowcell	0.971398	Pearson's Chi-squared test
RIN	0.6874792	Welch Two Sample t-test
RNA conc	0.7558127	Welch Two Sample t-test
Q30	0.7367614	Welch Two Sample t-test
GC content	0.2603974	Welch Two Sample t-test
Total reads	0.144702	Welch Two Sample t-test
Mapped reads	0.4158236	Welch Two Sample t-test

Table S6: The top 20 genes identified for the classification (sALS and controls) model using the Leave-One-Out (LOO) strategy. Four different feature sets were used as input: variance stabilised transformation counts for all 12,569 detected genes (ALL_{VST}) and 245 sALS-control differentially expressed genes (FDR_{VST}) or log counts per million for all 12,569 detected genes (ALL_{LCPM}) and 245 sALS-control differentially expressed genes (FDR_{LCPM}). Classification performance of each feature set was determined by four metrics (sensitivity, specificity, accuracy, area under the curve (AUC)) provided at the bottom of the table. d represents the number of genes in each feature set.

**ALL_{VST} (d = 12,569)**		**FDR_{VST} (d = 245)**		**ALL_{LCPM} (d = 12,569)**		**FDR_{LCPM} (d = 245)**	
**Gene**	**Count**	**Gene**	**Count**	**Gene**	**Count**	**Gene**	**Count**
LOC107984421	144	*LOC107984421*	144	*RPL7L1*	144	*RPL7L1*	144
RPL7L1	136	*MED13L*	140	*LOC107984421*	143	*LOC107984421*	144
MED13L	134	*RPL7L1*	137	*PLEKHG4*	124	*PLEKHG4*	134
PLEKHG4	131	*PLEKHG4*	135	*MED13L*	102	*MED13L*	105
STAG3L4	86	*STAG3L4*	90	*LETMD1*	76	*LETMD1*	84
ZNF544	65	*ZNF544*	69	*ZNF544*	75	*ZNF544*	78
IFITM1	59	*JDP2*	67	*ELAC2*	71	*ELAC2*	77
JDP2	56	*IFITM1*	62	*STAG3L4*	70	*STAG3L4*	75
FAM30A	55	*RGS6*	61	*FAM30A*	57	*RGS6*	61
RGS6	49	*FAM30A*	60	*NFYC-AS1*	54	*NFYC-AS1*	60
PLEKHG5	45	*PLEKHG5*	46	*RGS6*	47	*FAM30A*	59
LINC02210	38	*LETMD1*	43	*SREBF2*	44	*SREBF2*	49
BICDL1	36	*LINC02210*	41	*BICDL1*	40	*BICDL1*	43
ELAC2	35	*BICDL1*	36	*RPS10-NUDT3*	38	*COG2*	36
LETMD1	34	*ELAC2*	35	*COG2*	36	*PLEKHG5*	32
COG2	32	*CA4*	34	*PLEKHG5*	30	*ERG28*	30
CA4	29	*COG2*	32	*GPN2*	27	*GPN2*	27
LOC101928595	27	*NFYC-AS1*	27	*ERG28*	26	*LINC02210*	22
PIK3IP1	26	*LOC101928595*	26	*PIK3IP1*	26	*ZNF783*	21
IL1R2	23	*ZNF783*	19	*IL1R2*	25	*LOC101928595*	18
Sensitivity	0.781	Sensitivity	0.792	Sensitivity	0.792	Sensitivity	0.781
Specificity	0.667	Specificity	0.688	Specificity	0.75	Specificity	0.729
Accuracy	0.743	Accuracy	0.757	Accuracy	0.778	Accuracy	0.764
AUC	0.814	AUC	0.825	AUC	0.829	AUC	0.826

Table S8: The top 20 genes identified for prediction of disease duration regression model using the Leave-One-Out (LOO) strategy and gene expression data as input. Four different feature sets were used as input: variance stabilised transformation counts for all 12,569 detected genes (ALL_{VST}) and 245 sALS-control differentially expressed genes (FDR_{VST}) or log counts per million for all 12,569 detected genes (ALL_{LCPM}) and 245 sALS-control differentially expressed genes (FDR_{LCPM}). All 96 sALS were subjected to analysis. Regression model performance of each feature set was determined by four metrics (R2 score, Pearson's correlation, mean absolute error (MAE), mean absolute percentage error (MAPE)) provided at the bottom of the table. d represents the number of genes in each feature set.

**ALL$_{VST}$ (d = 12,569)**		**FDR$_{VST}$ (d = 245)**		**ALL$_{LCPM}$ (d = 12,569)**		**FDR$_{LCPM}$ (d = 245)**	
**Gene**	**Count**	**Gene**	**Count**	**Gene**	**Count**	**Gene**	**Count**
SMPDL3B	95	*SEPTIN11*	96	*MSANTD3*	96	*PCSK6*	90
THTPA	90	*PCSK6*	75	*SMPDL3B*	95	*SEPTIN11*	85
MSANTD3	84	*CKB*	73	*THTPA*	70	*CKB*	81
ATP5F1C	68	*ZNF544*	67	*LOC105376995*	57	*HNRNPA1*	74
PABPC1L	51	*HNRNPA1*	67	*ATP5F1C*	54	*CYB561A3*	62
PLXNB1	45	*CYB561A3*	66	*ANKLE2*	48	*ZNF544*	56
SH3KBP1	40	*LYPD2*	50	*PLXNB1*	45	*P2RY13*	45
ACTG1	39	*PHC1*	49	*PNMA6A*	35	*PHC1*	44
MRPL18	37	*HNRNPM*	42	*PABPC1L*	35	*FBXO10*	44
ANKLE2	34	*ZNF530*	42	*MRPL18*	34	*ZNF530*	41
HDC	27	*P2RY13*	41	*HDC*	29	*HNRNPM*	37
PTCD1	25	*FBXO10*	39	*ZNF699*	25	*MKNK1*	33
TPM4	25	*MKNK1*	27	*PTCD1*	24	*SNX22*	29
GMEB2	20	*FOS*	24	*TPM4*	24	*PNPLA1*	28
PCSK1N	18	*LOC101928595*	22	*BTN2A2*	22	*FOS*	23
ZNF699	18	*SNPH*	20	*PCYT1B*	19	*C8orf33*	18
LOC100507006	15	*PNPLA1*	17	*LOC100507006*	19	*LYPD2*	17
PNMA6A	14	*SNX22*	16	*SH3KBP1*	15	*AQP10*	17
WASHC3	14	*LETMD1*	13	*GMEB2*	12	*LETMD1*	14
SRSF3	11	*C8orf33*	13	*ESAM*	11	*LOC101928595*	14
R^2	0.7258	R^2	0.5571*	R^2	0.7393	R^2	0.5236*
Pearson's corr.	0.4999	Pearson's corr.	0.1608*	Pearson's corr.	0.567	Pearson's corr.	0.1889*
MAE	31.16	MAE	36.84	MAE	30.94	MAE	37.49
MAPE (%)	56.39	MAPE (%)	77.54	MAPE (%)	56.53	MAPE (%)	77.79

* One extreme outlier (Case61), based off predicted disease duration, was removed when calculating these values.

Table S9: The top 20 genes identified for prediction of disease duration regression model using the Leave-One-Out (LOO) strategy, and gene expression and clinical data as input. Four different feature sets, each accompanied by clinical data (sex, age at collection, site of onset) were used as input: variance stabilised transformation counts for all 12,569 detected genes (ALL_{VST}) and 245 sALS-control differentially expressed genes (FDR_{VST}) or log counts per million for all 12,569 detected genes (ALL_{LCPM}) and 245 sALS-control differentially expressed genes (FDR_{LCPM}). The 84 sALS known to be deceased were subjected to analysis. Regression model performance of each feature set was determined by four metrics (R2 score, Pearson's correlation, mean absolute error (MAE), mean absolute percentage error (MAPE)) provided at the bottom of the table. d represents the number of genes in each feature set.

**ALL_{VST} (d = 12,569)**		**FDR_{VST} (d = 245)**		**ALL_{LCPM} (d = 12,569)**		**FDR_{LCPM} (d = 245)**	
**Gene**	**Count**	**Gene**	**Count**	**Gene**	**Count**	**Gene**	**Count**
THTPA	84	*ZNF544*	67	*THTPA*	84	*ZNF544*	79
FGFR1	78	*BICDL1*	50	*FGFR1*	84	*BICDL1*	50
ATP5F1C	74	*ITGB7*	48	*ATP5F1C*	63	*P2RY13*	49
PLEKHA3	55	*HNRNPA1*	46	*MSANTD3*	58	*ITGB7*	49
NIPSNAP3B	48	*LYPD2*	43	*DIMT1*	50	*HNRNPA1*	43
MLLT6	44	*ZNF512B*	38	*NIPSNAP3B*	43	*LYPD2*	42
PMM1	42	*P2RY13*	36	*LOC100507006*	37	*RERE-AS1*	39
DIMT1	36	*SLC25A42*	28	*LOC107985115*	36	*SEPTIN11*	35
LOC107985115	32	*SEPTIN11*	25	*PMM1*	35	*C8orf33*	34
KRBA1	30	*NSUN7*	25	*DHRS7B*	28	*ANO9*	33
MSANTD3	30	*C8orf33*	25	*TSPOAP1*	27	*PHC1*	30
TSPOAP1	27	*ANO9*	25	*LAPTM4A*	25	*NFATC1*	28
DHRS7B	26	*TNFRSF13C*	24	*NDUFA6*	24	*NSUN7*	24
NDUFA6	25	*RERE-AS1*	22	*MLLT6*	22	*TNFRSF13C*	23
EIF1AY	19	*OSBPL3*	21	*SPOCK2*	21	*PKD1P5-LOC105376752*	21
SPOCK2	16	*PKD1P5-LOC105376752*	18	*USP9Y*	20	*ZNF512B*	21
LOC100507006	15	*GABPB2*	18	*NUTM2D*	19	*NELFCD*	19
ZDHHC9	13	*NFATC1*	17	*ZDHHC9*	13	*SLC25A42*	18
CYB561	12	*NELFCD*	16	*PLD6*	12	*ILRUN*	17
TSNARE1	12	*CAD*	16	*PNMA6A*	12	*OSBPL3*	14
R^2	0.7433	R^2	0.7101	R^2	0.7518	R^2	0.7047
Pearson's corr.	0.3666	Pearson's corr.	0.2097	Pearson's corr.	0.4061	Pearson's corr.	0.1924
MAE	22.32	MAE	25.21	MAE	20.74	MAE	25.11
MAPE (%)	44.91	MAPE (%)	51.36	MAPE (%)	42.56	MAPE (%)	50.94

Table S10: Statistical results from comparison of sample features between controls (n=48) and early-stage (n=28) or late-stage (n=28) sALS patients. Significant values ($p < 0.05$) are shown in bold. Site of onset, age at onset and disease duration were compared between early and late sALS collection groups only.

Sample variable	p-value	Statistical test
Sex	0.719	Pearson's Chi-squared test
Age	0.08163	Kruskal-Wallis rank sum test
Flowcell	0.4581	Fisher's Exact Test
RIN	0.688	One-way ANOVA
RNA conc	0.1896	Kruskal-Wallis rank sum test
Q30	0.8172	Kruskal-Wallis rank sum test
GC content	0.625	One-way ANOVA
Total reads	0.1992	Kruskal-Wallis rank sum test
Mapped reads	0.5497	Kruskal-Wallis rank sum test
Site of onset	0.8175	Pearson's Chi-squared test
Age at onset	0.5735	Welch Two Sample t-test
Disease duration	0.584	Kaplan-Meier estimate

Table S11: Statistical results from comparison of sample features between four sALS subgroups identified by clustering analysis. Significant values ($p < 0.05$) are shown in bold. Post-hoc tests are shown in italics.

Sample variable	p-value	Statistical test
Sex	0.9523	Fisher's Exact Test
Age	**0.002941**	Kruskal-Wallis rank sum test
0 – 1	*0.053*	*Post-hoc pairwise comparisons using Wilcoxon rank*
0 – 2	***0.038***	*sum exact test with Benjamini-Hochberg correction*
0 – 3	***0.009***	
Flowcell	0.1346	Fisher's Exact Test
RIN	0.145	One-way ANOVA
RNA conc	0.08718	Kruskal-Wallis rank sum test
Q30	0.1368	Kruskal-Wallis rank sum test
GC content	0.7875	Kruskal-Wallis rank sum test
Total reads	0.4246	Kruskal-Wallis rank sum test
Mapped reads	0.3096	Kruskal-Wallis rank sum test
Site of onset	0.6969	Fisher's Exact Test
Age at onset	**0.00337**	One-way ANOVA
0 – 1	*0.1455*	*Post-hoc comparison using Tukey multiple*
0 – 2	*0.1027*	*comparisons of means*
0 – 3	***0.0051***	
Disease duration	0.826	Kaplan-Meier estimate

Supplementary Methods

Confounder identification and adjustment

Available biological (sex, age) and technical (flowcell, RIN, RNA concentration, Q30 percentage, GC content, total reads post-trimming, Salmon mapped reads percentage) variables were considered as potential confounders of gene expression. Examination of the top 500 variable genes highlighted separation by sex across PC1 (Figure S1a) while PC2 and PC3 were associated with sample GC content. GC content was highly correlated with PC1 when examining all expressed genes (ρ = 0.72; Figure S1b,c) and demonstrated high correlation with most technical covariates (Figure S1d). Furthermore, GC content best improved the base model, reducing BIC for 67.8% of expressed genes. Correction for GC content using the limma removeBatchEffect function [20] removed correlations between technical covariates and the top 10 PCs (Figure S1e,f). Consequently, sex and sample GC content were included as confounders in all linear models ($\sim$ Sex + GC content + disease status). Age was not included in linear models as its inclusion as a covariate would incorrectly assume a constant fold change in expression across genes with age and when converted to a factor, it did not clearly associate with the top 10 principal components. Other examined variables were not included in linear models as they demonstrated correlation with GC content (multicollinearity problem), did not substantially increase the number of genes with improved BIC when added to the base model and/or did not show separation across or correlation with the top 10 PCs following correction for GC content.

Weighted Gene Correlation Network Analysis

Network construction and module detection

Weighted gene co-expression network analysis was performed using the WGCNA package in R v1.72.1 [34] using the 96 sALS cases only. The gene co-expression network was calculated as a signed adjacency matrix, using a pairwise bi-weight midcorrelation metric with a maximum percentile outlier threshold of 0.10, and a soft thresholding power of 12 to approximate a scale-free topology. The topological overlap matrix (TOM) of the network was calculated, and the TOM dissimilarity (that is, 1 - TOM) was used as a distance metric for clustering. Hierarchical clustering was conducted using the hclust, and modules of genes were constructed using dynamic tree cutting, using a deep split sensitivity of 2, and a minimum cluster size of 20. Eigengenes were determined for each module, and the pairwise Pearson correlation of eigengenes from all modules was calculated. Modules with highly correlated eigengenes (that is, a Pearson correlation coefficient of $>$0.75) were merged.

Associating modules with clinical traits

Association of modules eigengenes with clinical traits (sex, age at collection, age at disease onset, site of disease onset, disease duration, and collection point) were calculated using Spearman correlation, and relationships with FDR$<$0.05 were considered significant. Gene significance for a given gene and trait was calculated as the Pearson correlation of the gene and trait. Module membership for a given member gene was calculated as the Pearson correlation of the member gene and module eigengene. For modules identified to be significantly associated with clinical traits, GO biological processes and KEGG pathway enrichment analysis was performed using Metascape 3.5 with default parameters.

Module preservation between sALS and controls

To determine whether co-expression modules identified in sALS WGCNA were preserved in

control expression data, the modulePreservation function in the WGCNA package was used. Normalised gene counts for both sALS (n=96, reference network) and controls (n=48, test network) was input alongside sALS module definitions. Default parameters were retained for all arguments except nPermutations, where 200 was used.

Machine learning

Components

A machine learning pipeline consisting of three major components was implemented: 1) a feature selection model to identify genes that are predictive of disease status and duration, 2) a classification model to differentiate sALS cases and controls, and 3) a regression model to predict disease duration. All machine learning was conducted in Python v3.8.2 [40].

Feature selection

The goal of feature selection is to identify a subset of input variables (genes) that are predictive of output values (i.e., disease status and duration). We implemented the Hilbert Schmidt Independence Criterion Lasso (HSIC Lasso) [35], an advanced feature selection method which takes into consideration the joint contribution of different features and the non-linear input-input and input-output relationships. By default, the Gaussian kernels were used to model feature distributions and the top 20 features were selected based on their features relatedness score. The feature selection algorithm was implemented in Python based on the pyHISCLasso library v1.4.2 (https://pypi.org/project/pyHSICLasso/).

Classification model

To identify genes that can differentiate sALS cases from controls, we followed a previous study [36] and used the selected features to train a Random Forest model [37] for sALS and control classification. We used the default parameters (number of trees: 100; max depth of the tree: 5) to initialize the algorithm. The trained classifiers not only predict a class label, but also outputs a probability that a sample belongs to a particular class (i.e., sALS or control). By default, these classifiers classify a case with $\geq$50% probability as sALS. We tuned this threshold to 60% to remedy the unbalanced sample distribution and to make it easier to predict the minority class. The Random Forest classification model was implemented in Python based on the scikit-learn library v0.23.2 (https://scikit-learn.org/).

Regression model

A regression model was implemented to predict disease duration in sALS. Disease duration distribution was heavily skewed, with the majority (60%) of cases having disease durations $<$5 years (Figure 3a). To model the tailed distribution of the disease duration values, we used a Tweedie distribution [38], a special case of exponential dispersion models whose skewness can be controlled by a power parameter (r=1.6 in this study). A generalised linear model based on the Tweedie distribution was then built to predict sALS patients' disease duration. The Tweedie regression models was implemented in Python based on the scikit-learn library.

Performance evaluation

Leave-One-Out (LOO) cross-validation was used to evaluate the effectiveness of selected features in classification and regression models. In LOO cross-validation, one model will be created and evaluated for each sample in the data set, whereas the other samples are used to train the model. For the classification task, feature selection (using HSIC Lasso) and classification (using Random Forest) were run 144 times (96 sALS, 48 controls). 20 features were selected in each run and the counts of occurrence of the selected features were captured. In LOO cross-validation,

using the selected features, the classification models were trained with all samples except for one and then evaluated on the holdout sample. The classification model's performance was evaluated using sensitivity, specificity, accuracy and area under the receiver operating characteristic curve (AUC). For the regression task, feature selection and regression model training were run 96 times (96 sALS). The 20 features with the highest frequencies were selected. The regression model's performance was evaluated using coefficient of determination (R2 score), Pearson's correlation, Mean Absolute Error (MAE) and Mean Absolute Percentage Error (MAPE).

Validation of the classification model

RNA-seq data processing for the independent data set

The performance of the sALS-control classification model was tested in an independent data set consisting of whole blood RNA-seq from 30 *C9orf72* expansion negative ALS patients and 30 healthy controls [39]. FASTQ files were downloaded from the NCBI Sequence Read Archive (accession number PRJNA715316) and were processed in the same manner as the FASTQ files from the present study. Of note, a mean of 47.4% of reads (minimum: 38.0%, maximum: 61.6%) from the independent data set mapped to the GRCh38 transcriptome reference using Salmon. In contrast, samples generated in the present study demonstrated a higher mapping rate mean of 91.9% (minimum: 81.2%, maximum 97.9%) using the same strategy. Nevertheless, given the high sequencing depth (mean 79 million reads per sample) and the absence of other appropriate ALS-control whole blood RNA-seq data sets, we proceeded with this data set for validation of the classification model.

Confounder identification and adjustment for the independent data set

Available biological (sex, age) and technical (RIN before shipping, RIN, RNA concentration, RNA yield, GC content, total reads, Salmon mapped reads percentage) were considered as potential confounders of gene expression. When considering the top 500 variable genes, 2D visualisation of principal components (PCs) highlighted clear separation by sex across PC1-PC3. When considering all genes, age, RIN, GC content and Salmon mapped reads percentage each demonstrated some degree of correlation with the top 10 PCs (Figure S4a). Of all potential confounders, Salmon mapped reads percentage best improved the base $\sim$ disease status model, reducing BIC for 40.4% of genes. Salmon mapped reads percentage was also found to be highly correlated with GC content (Pearson correlation coefficient = 0.74) and moderately correlated with RIN (Pearson correlation coefficient = 0.42)(Figure S4b). Both age (p=0.0184885) and RIN (p=0.0002466) were identified to be significantly different between ALS and control groups (Figure S4c) and were therefore, excluded from confounder adjustment to prevent potential removal of true ALS-control differences. Following filtering of low count genes using edgeR filterByExpr function (17,857 genes remaining), gene counts from all 60 samples underwent transformation to log counts per million (CPM) using edgeR cpm function [18]. The limma removeBatchEffect function [19] was then applied to transformed counts to correct for Salmon mapped reads percentage (Figure S4d).

Testing the classification model on the independent data set

We tested our best performing sALS-control classification model (ALL_{LCPM}, trained using feature set 3) on the prepared gene expression data from the independent data set. The classification model achieved an accuracy of 45.0% (sensitivity: 43.3%, specificity: 46.7%, AUC: 42.4%). The distributions of the 20 genes composing the classification model were identified to be markedly different between the original training data set (n=144) and the test data set (n=60) (Figure S4e). Consequently, we attempted to normalise the distribution of the test set to align

it with the training data set. This normalisation process involved: 1) transforming the test data set's distribution into a standard normal distribution and 2) subsequently adjusting the distribution mean and standard deviation to match that of the training data set. Using the normalised test data set, the classification model achieved an accuracy of 63.3% (sensitivity: 60.0%, specificity: 66.7%, AUC: 64.7%), falling short of the LOO cross-validation performance achieved by the original training data set.
